# Global Metabolomic Profiling of Host Red Blood Cells Infected with Babesia divergens Reveals Novel Antiparasitic Target Pathways

**DOI:** 10.1128/spectrum.04688-22

**Published:** 2023-02-14

**Authors:** Divya Beri, Manpreet Singh, Marilis Rodriguez, Naman Goyal, Giselle Rasquinha, Yunfeng Liu, Xiuli An, Karina Yazdanbakhsh, Cheryl A. Lobo

**Affiliations:** a Department of Blood-Borne Parasites, Lindsley F. Kimball Research Institute, New York Blood Center, New York, New York, USA; b Department of Membrane Biology, Lindsley F. Kimball Research Institute, New York Blood Center, New York, New York, USA; c Department of Complement Biology, Lindsley F. Kimball Research Institute, New York Blood Center, New York, New York, USA; d Georgetown University, Washington, DC, USA; Weill Cornell Medicine

**Keywords:** *Babesia*, *Babesia divergens*, global metabolomics, RBC infection, babesiosis, lipids, Bd37, cholesterol, DAG, TAG, orlistat, invasion, egress, STED, infected RBC, lipid profile, *Plasmodium*

## Abstract

Babesia divergens is an apicomplexan parasite that infects human red blood cells (RBCs), initiating cycles of invasion, replication, and egress, resulting in extensive metabolic modification of the host cells. Babesia is an auxotroph for most of the nutrients required to sustain these cycles. There are currently limited studies on the biochemical pathways that support these critical processes, necessitating the high-resolution global metabolomics approach described here to uncover the metabolic interactions between parasite and host RBC. Our results reveal an extensive parasite-mediated modulation of RBC metabolite levels of all classes, including lipids, amino acids, carbohydrates, and nucleotides, with numerous metabolic species varying in proportion to the level of infection. Many of these molecules are scavenged from the host RBCs. This is in accord with the needs of a rapidly proliferating parasite with limited biosynthetic capabilities. Probing these pathways in depth, we used growth inhibition assays to quantitate parasite susceptibility to drugs targeting these pathways and stimulated emission depletion (STED) microscopy to obtain high-resolution images of drug-treated parasites to correlate changes in morphology with specific metabolic blocks in order to validate the data generated by the untargeted metabolomics platform. Thus, interruption of cholesterol scavenging from the host cell led to premature parasite egress, while chemical targeting of the hydrolysis of acyl glycerides led to the buildup of malformed parasites that could not successfully egress. This is the first report detailing the global metabolomic profile of the B. divergens-infected RBC. Besides deciphering diverse aspects of the host-parasite relationship, our results can be exploited by others to uncover further drug targets in the host-parasite biochemical network.

**IMPORTANCE** Human babesiosis is caused by apicomplexan parasites of the Babesia genus and is associated with transfusion-transmitted illness and relapsing disease in immunosuppressed populations. Through its continuous cycles of invasion, proliferation, and egress, B. divergens radically changes the metabolic environment of the host red blood cell, allowing us opportunities to study potential chemical vulnerabilities that can be targeted by drugs. This is the first global metabolomic profiling of Babesia-infected human red blood cells, and our analysis revealed perturbation in all biomolecular classes at levels proportional to the level of infection. In particular, lipids and energy flux pathways in the host cell were altered by infection. We validated the changes in key metabolic pathways by performing inhibition assays accompanied by high-resolution microscopy. Overall, this global metabolomics analysis of Babesia-infected red blood cells has helped to uncover novel aspects of parasite biology and identified potential biochemical pathways that can be targeted for chemotherapeutic intervention.

## INTRODUCTION

Intracellular erythrocytic parasites have established mechanisms to exploit their host red blood cells (RBCs) to ensure successful propagation. Babesia spp. are apicomplexan parasites that invade and infect the RBCs of various animal species, and a few of them have become zoonotic (1, 2). Among these are Babesia divergens, a cattle parasite seen primarily in Europe, although B. divergens-like variants have been found in the United States, Babesia microti, a rodent parasite that is responsible for most of the infections in the United States, and Babesia duncani, a few cases of which have been identified in California (3).

The pathology of babesiosis is a consequence of parasitemia, which develops through the cyclical asexual replication of Babesia parasites in host RBCs (4, 5). As is typical in such host-pathogen infections, the parasite must divert host nutrients to facilitate its continued proliferation, resulting in a metabolically impacted host red cell that struggles to maintain homeostasis. These infected host cells handle the impact of infection in the form of altered metabolic profiles that encompass by-products of parasite metabolism, including waste products and toxins (6). Hemolysis, hemoglobinuria resulting in low hematocrit, low hemoglobin level, low haptoglobin level, elevated reticulocyte count, and elevated lactate dehydrogenase level are some of the measurable effects of host pathogenesis caused by the metabolic intertwining of Babesia and host (7, 8). The metabolites required by Babesia to maintain successful parasitism can be scavenged whole from the host cell and/or environment or synthesized using building blocks available in the host cell.

However, little is known of Babesia species metabolic pathways, and this report is the first to characterize the global metabolic modulation of the host RBC by Babesia using high-resolution, global, untargeted metabolomics. This area is critical to dissect because the pathology of human babesiosis is linked to dysregulation of the human host metabolism (9, 10). Earlier reports have shown that intracellular parasites like Toxoplasma and Plasmodium have evolved to thrive with minimal metabolic pathways because of successful scavenging of their most essential nutrients from the host (11–14). Interestingly, human Babesia spp. have much smaller genomes than other apicomplexans, further pointing toward adaptation through evolution (15–20). Despite this, the parasite possesses crucial metabolic pathways that define its survival in the host. Therefore, global metabolomics studies have the power to unravel some of the more poorly understood aspects of Babesia biology, and these studies can have far-reaching clinical relevance in defining better therapeutic targets, as well as biomarkers of infection. Babesia’s metabolic network has so far been indirectly approached via inferences from genomic data or *in vitro* biochemical assays (15, 21–23). However, metabolomic profiling provides one of the most integrated functional profiles of biologic status, as it accurately measures functional phenotypes that are the net result of genomic, transcriptomic, and proteomic changes (24).

In this study, we present results from globally targeted mass spectrometry that quantified metabolic changes in parasite-infected erythrocytes in comparison to uninfected erythrocytes during the asexual life cycle of B. divergens in the human RBC. As synchronized parasite populations exist only for a fleeting few hours after invasion (25), analyses were conducted on asynchronous parasite cultures under both low and high infection conditions, comparing their metabolic profiles to that of uninfected host RBCs (uRBCs). To comprehensively analyze the parasite metabolism using metabolomic data collected during its asexual erythrocytic cycle, a series of computational and statistical methods were used and key global, pathway-level, and stage-specific metabolites were identified. Our study has revealed important changes in nucleotide, lipid, amino acid, and carbohydrate metabolites, most of which were associated with Babesia proliferation. We identified 650 metabolites, of which approximately 25% and 50% were differentially present in low and high infection, respectively, compared to their presence in uRBCs. To corroborate the metabolomics profiles, we investigated in detail specific pathways that the global metabolomic analysis identified as critical to the rapidly proliferating parasite, specifically those that yield building blocks of the parasite biomembrane, focusing on cholesterol and free fatty acids. Our growth inhibition assays (GIAs) with drugs targeting major enzymes in these pathways, supported by stimulated emission depletion (STED) microscopy of parasites, confirm our global metabolomics findings and emphasize the importance of global metabolite analysis as a tool to study Babesia-RBC interactions and parasite biology, as chemical interruption of these pathways resulted in severe consequences for the proliferating parasites.

This paper provides the first overview of metabolic modulation of human red cells by B. divergens under both low and high infection conditions, highlighting the interplay between host and parasite metabolism. Importantly, these results also identify critical vulnerabilities of the parasite that can be exploited in the future for drug discovery efforts.

## RESULTS

### Global metabolic profiling of B. divergens-infected RBCs reveals major changes in the RBC metabolome upon infection.

Babesia spp. possess the smallest genomes sequenced among the apicomplexans. Genome-wide sequences of B. microti, B. divergens (Rouen 1987), Babesia bovis, and Babesia canis reveal their genome sizes to be 6.5 Mb, 10.7 Mb, 8.2 Mb, and 7.02 Mb (17, 26). The highly reduced genomes of these parasites are reflected in minimal metabolic pathways and auxotrophic lifestyles with high dependence on hosts for nutrients required for growth. To investigate the metabolic pathways critical to the asexual growth of B. divergens in human RBCs, we performed global metabolomics on uninfected red blood cells (uRBCs) and infected red blood cells (iRBCs), at low and high parasitemia (*n* = 3 for each set). Two infection levels were chosen to enable the identification of potential biomarkers for early and low infections, while data from the higher parasite load could potentially indicate vulnerabilities that can be targeted by therapeutic interruptions.

**FIG 1:**
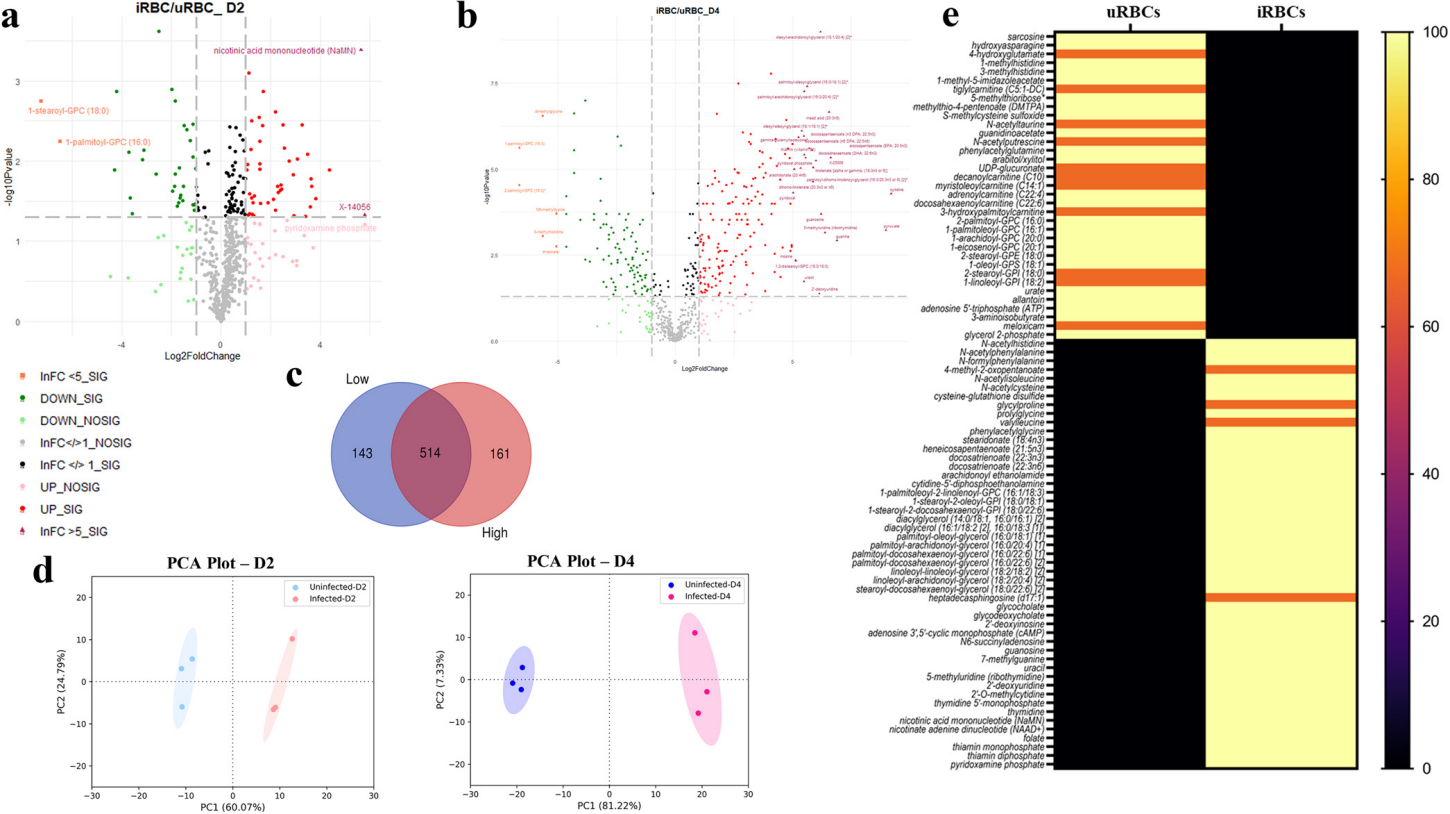
Global metabolic profiling of B. divergens-infected RBCs reveals major changes in the RBC metabolome on infection. (a and b) Volcano plots show the differential abundance of metabolites present in uRBCs and iRBCs on D2 (low parasitemia) and D4 (high parasitemia) postinvasion (*n* = 3 for each set at both time points). The *x* axis displays log_2_ fold change (log_2_FC; threshold = 2-fold equivalent to log_2_FC = 1) values, and the *y* axis displays −log_10_ *P* values (threshold significance cutoff, *P* < 0.05, equivalent to a −log_10_ *P* value of 1.301). Each dot represents a metabolite. Gray dots represent metabolites with log_2_FC values between −1 and 1 and nonsignificant *P* values. Black dots represent metabolites that passed the statistical threshold but not the log_2_FC threshold. Light green and light red circles show metabolites that passed the log_2_FC threshold but were not statistically significant and are labeled “UP_NOSIG” and “DOWN_NOSIG,” respectively. Red circles depict metabolites with 2 < log_2_FC < 5 and statistical significance (UP_SIG), and green circles depict metabolites with log_2_FC values between −1 and −5 (DOWN_SIG). Purple triangles show metabolites with log_2_FC values of >5 and statistical significance, which are identified by labels, while orange squares show metabolites with log_2_FC values of less than −5 and statistical significance, also labeled. Approximately 23% of metabolites were differentially present on D2, and ~50% on D4. (c) Venn diagram shows the numbers of common and exclusive metabolites measured on D2 and D4. Approximately 75% of metabolites were common between the two time points. (d) Principal component analysis (PCA) plot shows analysis for both sets (uRBCs at D2 in light blue, iRBCs at D2 in light pink, uRBCs at D4 in dark blue, and iRBCs at D4 in dark pink), separating along PC1 and PC2. As is evident, in both sets, the uRBCs are separated from the iRBCs along PC1, with a greater separation for D4 sample sets than for D2 sample sets. (e) The heat map depicts the metabolites obtained in our analysis that are unique to uRBCs and to iRBCs on D4. Black indicates the absence of detection of the particular metabolite in our analysis, orange represents detection of the metabolite in 2/3 replicates in the set, and light yellow refers to detection of the metabolite in all samples of the data set. As shown, several metabolites were uniquely found in uRBCs (top left), while several metabolites were uniquely found in iRBCs (bottom right) at D4.

Under low parasitemia conditions in infected cells collected on day 2 postinvasion (D2; 10% parasitemia), a total of 657 metabolites were detected, among which differences in the levels of 145 metabolites between uRBCs and iRBCs were statistically significant (*P* < 0.05). Of these, 101 were upregulated and 44 were reduced in abundance. This is represented as a volcano plot in Fig. 1a. A volcano plot is a scatterplot; the log_2_ fold change (log_2_FC) values are on the *x* axis, and the −log_10_ *P* values on the *y* axis. The volcano plots were constructed in RStudio using the package ggplot. The codes used to construct the plot have been provided in file 2 in the supplemental material. The plot characterizes metabolites based on differences between the two conditions, uninfected and infected, together with the associated statistical significance values. The log_2_FC threshold was set at more than or less than 1, and the *P* value threshold was set at a −log_10_ *P* value of >1.3010. Thus, only metabolites with an absolute FC of >2 or <2 and an absolute *P* value of <0.05 were considered for further analysis. In Fig. 1a and b, gray dots represent metabolites that had an FC or *P* value that was less than the threshold. The pale red and green dots represent metabolites with log_2_FC values of >1 and <1, respectively, but with insignificant *P* values. The red and green dots represent metabolites with log_2_FC values of >1 and <1, respectively, and significant *P* values. The purple triangles and orange squares represent metabolites with log_2_FC values of >5 and <5, respectively. In terms of log conversion, a log_2_FC of 1 corresponds to an absolute FC of 2, while a log_2_FC of 5 corresponds to an absolute FC of 32. As is evident in Fig. 1a, most metabolites detected were “gray,” indicating that at D2, the bulk of the metabolites detected did not differ substantially between uninfected and infected RBCs. However, some critical metabolites were differentially abundant at this time, including cytidine, pyruvate, CMP, pyridoxamine phosphate, and NaMN, each of which had an FC value of >10 between uRBCs and iRBCs. Thus, these molecules could be exploited as potential diagnostic tools to detect early infection.

In cells analyzed at the time of high parasitemia (50%) conditions on day 4 postinvasion (D4), a total of 673 metabolites were detected, among which the levels of expression of 325 metabolites were significantly different (*P* < 0.05) in uRBCs and iRBCs and an additional 34 metabolites with differential expression had *P* values between 0.05 and 0.1. Among these, totals of 233 metabolites were more abundant and 126 metabolites were less abundant in iRBCs than in uRBCs. Thus, more than twice the number of metabolites differed significantly in quantity as the level of infection increased from 10% of cells parasitized to 50% parasitemia. A volcano plot highlighting these differences was constructed (Fig. 1b). As can be seen from the plot, the maximum numbers of metabolites that were upregulated and downregulated with log_2_FC values greater than 5 belonged to the lipid class of biomolecules, followed by those involved in nucleotide metabolism (UMP, cytidine, 7-methyguanine, 2-deoxycytidine, guanine, ribothymidine, NAD, and inosine) and energy metabolism (pyruvate, isocitrate, and malate). These molecules are critical for the rapidly proliferating parasite, providing both the DNA building blocks and the energy required for parasite multiplication, and hence, they can be expected to be found in significantly greater amounts than in uninfected cells or early infection events. Overall, several key metabolites were differentially abundant in Babesia-infected red cells on both D2 and D4, corresponding to low and high parasitemia, with elevated FC values at the later time point. As shown in the Venn diagram in Fig. 1c, most of the metabolites detected were common between D2 and D4 (~75%); however, as described above, most of these metabolites were both at higher levels and showed a statistically significant FC at D4.

We next wanted to analyze the relationship between our data sets (uninfected D2, uninfected D4, infected D2, and infected D4) to identify the major differences. We used Python programming language to construct principal component analysis (PCA) plots, and the code used for constructing these plots can be found in file 2 in the supplemental material. Figure 1d shows a PCA plot with each point representing one data set. Light blue and dark blue dots are data sets from uRBCs at D2 and D4, respectively, while light red and dark red dots are data sets from iRBCs at D2 and D4, respectively. As is evident from the results in Fig. 1d, uRBCs separate from iRBCs along principal component 1 (PC1) in the PCA plot. The uninfected and infected data sets (shown in blue and red dotted circles) show significant separation along PC1. Interestingly, even though the RBC is technically metabolically quiescent *in vitro*, uRBCs cultured for different lengths of time at 37°C yielded slightly different metabolic profiles. This has also been shown extensively by related work in malaria, where the metabolic profiles of uninfected red cells cluster based on time spent in culture (12, 27).

One of the key objectives of this study was to identify molecules that were present exclusively in uRBCs or exclusively in iRBCs at high parasitemia (D4). This analysis of unique metabolites could provide insights into metabolic pathways that are specifically impacted by high Babesia infection. Figure 1e shows a heatmap with unique metabolites in uRBCs in the top of the graph and iRBCs in the bottom. Pale yellow indicates the presence of the metabolite in all samples of that group, orange indicates the presence of the metabolite in 66% of the samples in the group, and black indicates the complete absence of the metabolite in all samples of the group. As seen from the results in Fig. 1f, several of the lysophospholipids were seen exclusively in uRBCs, while several diacylglycerols (DAGs) were present only in iRBCs. Therefore, there appears to be an extensive lipid remodeling of host cells upon Babesia infection, which provides a unique lipid signature specific to iRBCs as opposed to uRBCs. Other interesting metabolites uniquely present in iRBCs were those belonging to glutathione metabolism and redox metabolism (*N*-acetylcysteine and cysteine-glutathione disulfide), cAMP, folate, and derivatives of thiamine. Redox metabolism has been implicated as playing a major role in protozoan parasites like Plasmodium (28–31) and, more recently by us, in Babesia (32). cAMP has also been implicated as playing an essential role in signaling in other parasites (33).

### Pathway-specific comparison of uRBCs and B. divergens-infected RBCs identifies specific metabolic pathways impacted by infection.

**FIG 2:**
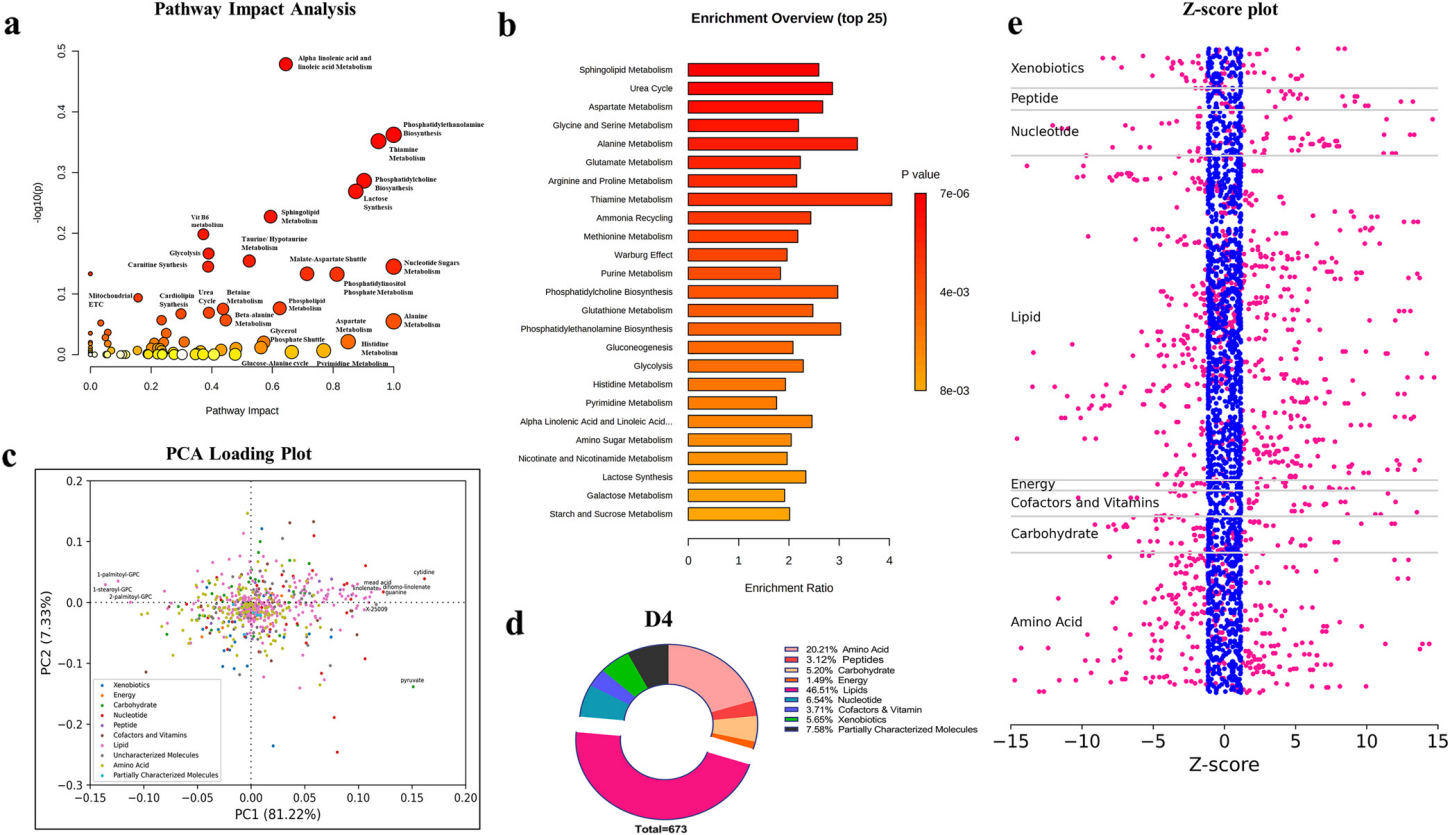
Pathway-specific comparison of uRBCs and B. divergens-infected RBCs identifies specific metabolic pathways impacted by infection. (a) The Pathway Analysis module of MetaboAnalyst 5.0 was used to generate the plot, which combines results from pathway enrichment analysis and pathway topology to depict the most relevant pathways in the current set. The *x* axis defines the *P* value, while the *y* axis calculates the impact of the pathway in the metabolome analysis. The white to yellow to orange color gradation defines the significance of the pathway in the analysis, with red being the most significant. The radius of the circle defines the number of metabolites obtained in the pathway. (b) An overrepresentation analysis (ORA) plot was generated in MetaboAnalyst 5.0. The plot shows the top 25 metabolic pathways that were enriched in our analysis on D4 postinvasion (high parasitemia). (c) The PCA loading plot constructed from data from D4 postinvasion shows the different metabolites belonging to color-coded superpathways and labels the top 10 metabolites that contributed to differences between the uRBCs and iRBCs on D4. The farther away from the axis the metabolite is on the PC1 and PC2 axes, the higher its impact on the PCA. Lipids (pink) are the highest contributors to the differences observed between uRBCs and iRBCs. (d) The pie chart shows the percentage of metabolites belonging to each of the superpathways in the D4 metabolomic analysis. Lipids formed the largest class of metabolites observed in this study. (e) A z-score plot is shown where metabolites are segregated according to the superpathways. Each dot is a metabolite in each of the samples in the D4 sample set. Pink dots represent metabolites in the uRBCs, while blue dots represent metabolites in the iRBCs. The positions of the dots (z-scores) represent the distribution of the values for the metabolite, and the number of standard deviations is represented by the distance from the mean value. As is evident, metabolites belonging to the iRBCs (pink) deviate along both sides of the *x* axis, while uRBCs are concentrated in the center of the plot.

As shown by our in-depth metabolomic analysis, several metabolites across all major classes were differentially abundant in iRBCs versus uRBCs at both low and high parasitemia (Fig. 1 and b). In this section, we analyze different aspects of the modification of metabolites in the infected sample set at high infection (D4) compared to uRBCs. We chose this data set for further analysis as it contains the maximal infection-related changes when compared to the set at low parasitemia, which provides a glimpse of early modifications in the metabolomics of host cell. To further discern the metabolic pathways with maximal impact, we constructed a pathway impact analysis plot with MetaboAnalyst 5.0 (Fig. 2a) using the data from the high parasitemia culture set (D4), and the report generated is available in file 3 in the supplemental material. This pathway impact plot (Fig. 2a) shows the pathway impacts on the *x* axis and −log_10_ *P* values on the *y* axis. Each circle represents a metabolic pathway; the higher its value on the *x* axis, the higher is the impact of the pathways, and the impact is color coded along a gradient of white to yellow to orange to red in Fig. 2a. The radius of the circle is determined by the number of metabolites identified in our analysis that belong to the specific metabolic pathway. Overall, a variety of metabolic pathways are impacted, prominent among which are phosphatidylcholine (PC) synthesis, phosphoinositide (PIP) metabolism, sphingolipid metabolism, aspartate metabolism, glycolysis and mitochondrial, etc.

Furthermore, we used MetaboAnalyst 5.0 to create a metabolite set enrichment analysis (MSEA), which provides a way to identify biologically meaningful patterns that are significantly enriched in the global metabolomic data. This analysis is analogous to gene set enrichment analysis (GSEA), typically performed for genomics data sets. It has the potential to identify subtle but consistent changes among a group of related compounds that may go undetected using conventional approaches. Within the MSEA, an overrepresentation analysis (ORA) was performed to identify pathways that may have been overrepresented in the data set, and the respective one-tailed *P* values are shown as a color gradient from yellow to red, with the latter being more significant. As seen in Fig. 2b, the top 25 metabolic pathways are listed, and the metabolic enrichment ratio is plotted on the *x* axis. The major pathways identified in this analysis were those belonging to sphingolipid metabolism, PC and phosphatidylethanolamine (PE) metabolism, glycolysis, gluconeogenesis, amino acid metabolism, and glutathione metabolism. The results from this analysis are available in file 5 in the supplemental material.

Next, we constructed a PCA loading plot in Python matplotlib to identify the key metabolite contributors to differences arising between uRBCs and iRBCs on D4 (Fig. 2c). A PCA loading plot provides information about the variables that make the largest contributions to the two components: PC1 and PC2. The loading plot values range from −1 to +1, with values close to 0 indicating a weak influence on the component. The greater the absolute value is, the greater is the contribution of the variable to the respective component. The sign of a loading mark (+ or −) indicates whether a variable is positively or negatively correlated to the PC. As shown by results in Fig. 2d, the biomolecular class of lipids and then amino acids are strong contributors to variations seen between uRBCs and iRBCs on D4. The top 10 absolute contributors to the variation along PC1 are labeled. Among them, three lysophospholipids are negatively correlated to PC1, while the remaining 7 metabolites are positively correlated to PC1. Among the latter, three metabolites are lipids (mead acid, linolenate, and dihomo-linolenate), one is an uncharacterized molecule, two are nucleotides (guanine and cytidine), and one is a key energy molecule, pyruvate. To obtain a strict quantification of significantly altered metabolites among major metabolic pathways, we constructed a pie chart for the metabolites obtained in uRBCs and iRBCs on D4 (high parasitemia). As evident from the chart, in both cases, lipids contributed >40% of the differentially present metabolites, followed by amino acids (~20%) and then nucleotides and carbohydrates (Fig. 2d).

To further analyze the pathways contributing to maximal changes upon infection of the RBCs, we constructed a z-score plot (Fig. 2e) using the values of metabolites obtained in uRBCs and iRBCs on D4 and segregating them based on the superpathways (as defined in file 5 in the supplemental material). Each dot corresponds to a metabolite from each sample (*n* = 3) of each group (uRBCs in blue and iRBCs in pink). We used the z-score, which calculates the number of standard deviations by which the data point is distant from the average of the group and is thus an ideal way to standardize values, for easier interpretation of data (Fig. 2e). If the z-score is positive, it means that the score is above the average value, whereas a negative z-score would indicate that the score is below the average value. As seen by the results in Fig. 2e, most metabolites in the uRBCs (blue) are concentrated between −2 and +2 on the *x* axis, while metabolites from iRBCs for different pathways are scattered on both sides of the *x* axis, with the highest concentration of data points on the positive side of the *x* axis. Thus, this reiterates that there is a dynamic progression of metabolic activity upon Babesia infection, resulting in upregulation of several molecules in the host RBC.

### Dissection of changes in the major metabolic pathways using biomolecular classifiers reveals significant changes across major superpathways.

**FIG 3:**
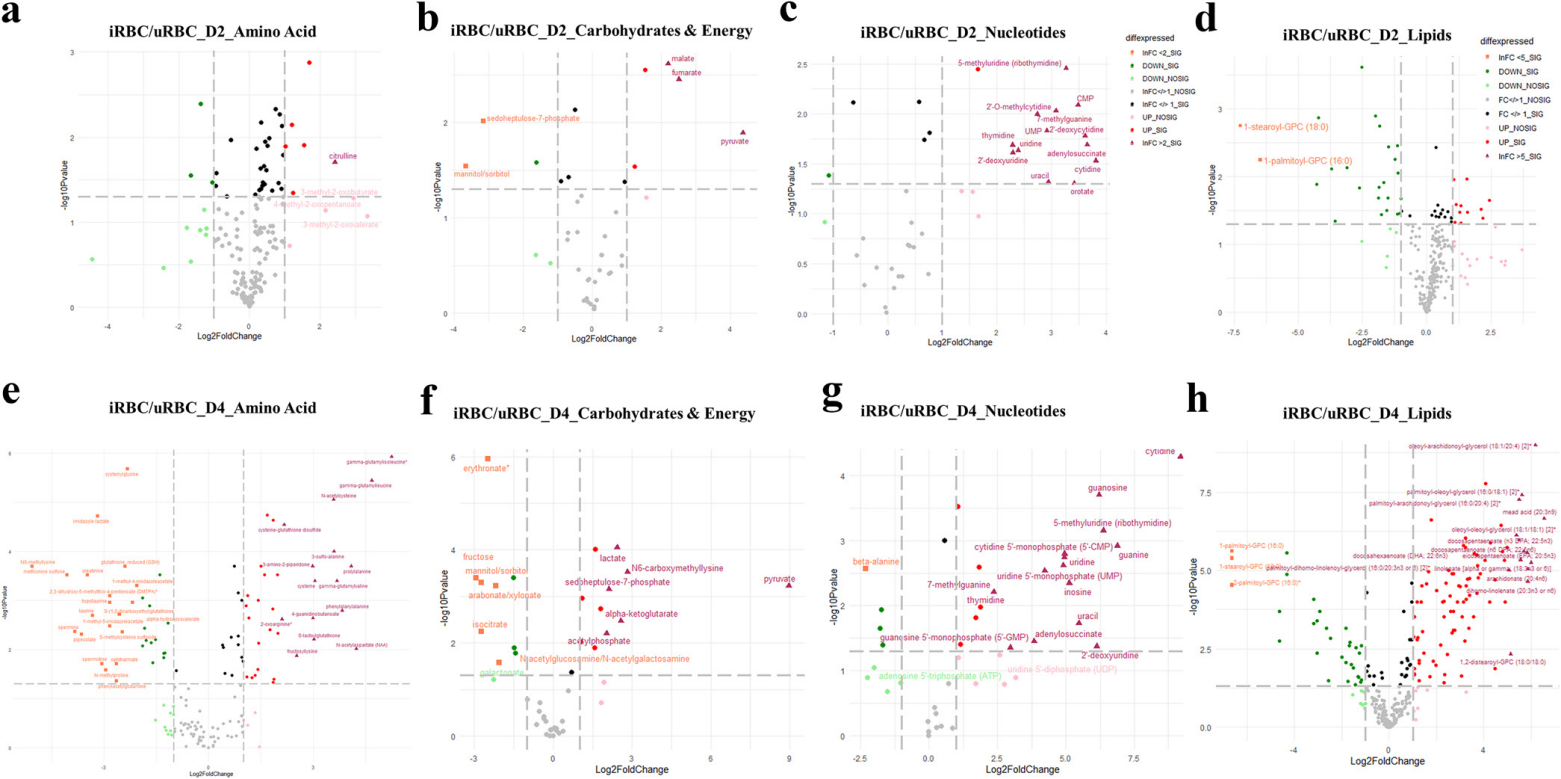
Volcano plots of differential metabolites across different classes show dramatic changes at D2 and D4 postinvasion. The volcano plots are scatterplots with −log_10_ *P* values (cutoff at *P* < 0.05) on the *y* axis and log_2_ fold change (log_2_FC) values on the *x* axis. Black dots refer to metabolites with log_2_FC values between −1 and +1 and significant *P* values; gray dots represent metabolites with log_2_FC values between 1 and +1 but with nonsignificant *P* values; light green and light red dots represent metabolites with log_2_FC values between −1 and −2 or +1 and +2, respectively, and significant *P* values, except for lipids, where they refer to log_2_FC values between −1 and −5 or +1 and +5, respectively, with significant *P* values; and orange squares and purple triangles represent metabolites with log_2_FC values of less than −2 or more than +2, respectively, and significant *P* values, except for lipids, where they represent log_2_FC values of less than −5 or more than +5, respectively, with significant *P* values. (a to d) Volcano plots were constructed from data from D2 postinvasion and represent metabolites belonging to amino acid (a), carbohydrate and energy (b), nucleotide (c), and lipid (d) pathways. (e to g) Plots were constructed from data from D4 postinvasion. As is shown, the numbers of metabolites observed with differential expression were drastically increased at the later time point (D4) that corresponded to higher parasitemia.

We then constructed volcano plots for both infection groups, D2 and D4, based on the fold change values obtained for the top superpathways (as outlined in the Excel sheets in files 4 and 5 in the supplemental material), which were lipids, amino acids and peptides, nucleotides, and carbohydrates and energy (Fig. 3a to d for low parasitemia [D2], and Fig. 3e to h for high parasitemia [D4]).

**(i) Lipids.** In our analysis of the infected RBCs, different metabolic pathways of lipids were identified, namely, those associated with both monounsaturated fatty acids and polyunsaturated fatty acids (PUFA), phospholipids, phosphatidylcholine (PC), phosphatidylethanolamine (PE), phosphatidylserine (PS), phosphatidylinositol (PI), lysophospholipids, plasmalogens, monoacylglycerol (MAG), diacylglycerol (DAG), sphingolipids, ceramides, sterol, and bile acid metabolism. Membrane lipids are important modulators of the RBC rheology and are responsible for maintaining both membrane deformability and fluidity as the red cells pass through capillaries in the host. Across both infection levels, significant changes across all lipid classes were observed. As the parasite proliferates inside the RBCs, there is an enormous demand for lipids to form membranes of daughter cells (plasma membrane, membranes of organelles like mitochondria, and accumulation of fat in lipid bodies) (34). Despite this high demand for lipids, it is most likely, based on its genome analysis, that Babesia does not synthesize most of its own lipids, much like Plasmodium and Toxoplasma (17). Additionally, the host RBCs cannot synthesize phospholipids, cholesterol, or free fatty acids (34). In the D2 (low parasitemia) group, the negative log fold change of iRBCs/uRBCs was maximal in the class of lysophospholipids (file 4 in the supplemental material), while the positive log fold change was maximal in the class of DAGs (Fig. 3d). As shown by the results in Fig. 3h, in the D4 (high parasitemia) group, the top downregulated metabolites were also lysophospholipids, albeit with a much higher fold change (file 5 in the supplemental material). Lysophospholipids are present in cell membranes and are known to aid in cell signaling and protein folding, as well as mobilizing intracellular Ca2^+^ stores; however, their role in the parasite has not been elucidated yet. The top 10 upregulated metabolites in the D4 set were diacylglycerols (DAGs) and long-chain polyunsaturated fatty acids (LC-PUFA), with the FC values obtained averaging more than 40-fold. Below, we describe some of the more striking differences observed in different lipid classes between uRBCs and iRBCs at both D2 and D4.

***(a)* Fatty acids and acyl glycerides.** The top upregulated metabolites in the high parasitemia cultures were LC-PUFA, followed by DAGs. DAGs and TAGs are the main glycerolipid species in the parasite and are used as a source of fatty acids that the parasite can use for membrane synthesis and beta oxidation. However, in our current study, TAGs were not detected on the global metabolomics platform due to their size and hydrophobicity. Therefore, early in infection, DAGs and lysophospholipids were the dominant differentially abundant lipids, and as infection progressed, these classes of lipids increased dramatically in amount; additionally, several lipids belonging to LC-PUFA were significantly upregulated. The differential expression of lipid metabolites on D2 and D4 is represented in Fig. 3d and 3h, respectively, and as evident, with an increase in the percentage of infection, the differential regulation of lipid expression was more acute, with a higher number of lipids dysregulated, along with higher significant fold changes in the amounts.

***(b)* Phospholipids and glycerophospholipids.** The major classes of phospholipids include phosphatidylcholine (PC), phosphatidylethanolamine (PE), and phosphatidylserine (PS). Compared to uRBCs, on D2, several phospholipids were upregulated 2- to 5-fold. Interestingly, PE and PS were not perturbed significantly at this time point, but there were changes associated with PC and PI. PC plays an important role in the structure of membranes but can also modulate cell signaling, as the hydrolysis of PC forms DAGs, which were highly differentially abundant between uRBCs and iRBCs (35).

***(c)* Sphingolipids.** Another lipid class that was differentially abundant between uninfected and infected RBCs was sphingolipids. These lipids possess a sphingosine backbone and can be acetylated to form ceramides, which in turn can be modified through the addition of phosphocholine or phosphoethanolamine to form sphingomyelins or glycosylated to form glycosphingolipids. Interestingly, while precursors to sphingolipids like sphingosine were enriched more than 10-fold in infected cells, the sphingomyelins and sphingosine were not. However, sphingosine-1-phosphate (S1P), which has been implicated as playing an important role in erythrocyte glycolysis and parasite survival, was also downregulated in this set by a log_2_ fold change of >3. Sphingomyelins can also be used to synthesize ceramides, which have been shown to be potent second messengers regulating different cellular processes. We found that a few ceramides, including ceramide (d18:1/14:0, d16:1/16:0) and *N*-palmitoyl-sphingadienine (d18:2/16:0), were increased ~5- to 6-fold upon infection.

**(ii) Amino acids and peptides.** As in other apicomplexans, *in silico* analysis of the genome of Babesia spp. suggests an absence of *de novo* amino acid synthesis pathways, implying that the parasite depends largely on the host cell and environment for acquiring these essential nutrients. It has been suggested that even in organisms like Toxoplasma gondii, pathways for the *de novo* biosynthesis of several amino acids were dispensable, especially in fast-replicating stages of the parasite (36). While hemoglobin, the dominant RBC cytoplasmic protein, can potentially be used by the parasite for scavenging required amino acids, the actual pathway of hemoglobin degradation has not been elucidated in B. divergens (37). In our analysis, at both experimental time points, amino acids were identified as differentially abundant in iRBCs compared to uRBCs. This has been represented using volcano plots of their abundances at D2 (low parasitemia) (Fig. 3a) and D4 (high parasitemia) (Fig. 3e).

Strikingly, the key metabolites in the γ-glutamyl cycle and glutathione metabolism were downregulated at D2 (low parasitemia), but as infection progressed, several of these intermediate γ-glutamyl-amino acid metabolites, *N*-acetylcysteine, *S*-lactoyl-glutathione, cysteine, glutamate, and 5-oxoproline, were upregulated severalfold, with significant reductions in cysteinyl-glycine and GSH (reduced glutathione). This suggests that with increased proliferation of the parasite, there is an increased reliance on this redox regulatory pathway, as has also been implicated in a recent research study from our laboratory (32). These findings also largely agree with studies on the metabolomics of Plasmodium falciparum-infected RBCs, where it has been shown that with the progress of infection, these redox pathway intermediates are elevated severalfold (29, 38). Interestingly, pipecolic acid, which is a product of lysine degradation that increases severalfold in both P. falciparum and P. vivax infections (29) and is projected to be a unique biomarker for malaria infection, was unaffected at low parasitemia and was significantly downregulated at higher parasitemia. Therefore, though these parasites are apicomplexans and evolutionarily close to each other, they seem to have unique metabolic pathways, which can be useful in both disease detection by biomarker analysis and developing new drugs targeting these pathways.

**(iii) Carbohydrate and energy pathways.** The intracellular growth of Babesia requires considerable energy expenditure, as within a short window of time, parasite multiplication results in 4 to 8 Babesia progeny. Using global and targeted metabolomics, several studies have highlighted the importance of energy pathways in parasites (12, 13, 39, 40). As shown by the results in Fig. 3b and f, multiple metabolites were differentially present in the energy pathways at both low and high parasitemia. Like Plasmodium, Babesia seems to depend largely on glycolysis for energy production and has all the enzymes required for glycolysis (17). RBCs are rich in glucose/hexose transporters. Not much is known about glucose transporters in B. divergens; however, in B. bovis, iRBCs were reported to accumulate far more glucose than uRBCs (41) and were not affected by human GLUT1 inhibitors at low concentrations (42). Interestingly, in our study, at both D2 and D4, pyruvate was massively upregulated in iRBCs (20-fold and 500-fold, respectively), along with an upregulation of lactate (2.3-fold and 5.4-fold, respectively). Another striking difference from Plasmodium is the lack of pyruvate dehydrogenase (PDH) in Babesia spp. PDH is responsible for converting pyruvate to acetyl-coenzyme A (acetyl-CoA) in the mitochondria, which then feeds into the tricarboxylic acid (TCA) cycle and is converted into ATP via the electron transport chain (ETC) and oxidative phosphorylation. However, in Toxoplasma gondii and Plasmodium berghei, PDH is targeted to the apicoplast, and this function is fulfilled by branched-chain ketoacid dehydrogenase (BCKDH) (43, 44). BCKDH is present in Babesia spp., but its functionality in converting pyruvate to acetyl-CoA has not yet been investigated.

*In silico* analysis of the B. divergens genome shows that genes encoding the various enzymes mediating the TCA cycle are present, and this was confirmed by the metabolite analysis in our study, showing alteration in key TCA metabolite levels; for example, citrate and isocitrate were unaltered on D2 but were significantly reduced in iRBCs compared to their levels in uRBCs on D4. Other key metabolites, such as fumarate, malate, and α-ketoglutarate, were increased on D2 (~2-fold) and D4 (~3- to 4-fold). These metabolites were differentially abundant in iRBCs and uRBCs, and thus, the parasite may also use mitochondrial aerobic respiration to fulfill some of its energy needs. Our data show that carbon flux and energy pathways are altered in host red cells upon infection, and future work will allow detailed analysis of the contributions of specific subpathways.

**(iv) Nucleotides.** Nucleotides are another important class of biomolecules that were differentially abundant in uRBCs and iRBCs. Intra-RBC proliferation of Babesia requires a constant supply of nucleotides for DNA replication to form daughter parasites. Each 1N parasite (“1N” refers to one genome copy) can proliferate to form 2N, 4N, and >4N parasites, and each of these higher forms can egress and infect new host cells (25). Not much is known about the synthesis and metabolic pathways used by the parasite. However, it has been shown that the parasite can incorporate tritiated hypoxanthine, adenine, and adenosine but not uridine, thymine, and thymidine, thus suggesting a differential regulation of purines versus pyrimidines in Babesia spp. (45). As seen in our analysis, at both D2 and D4, guanine, cytidine, and uracil and their derivatives were enormously abundant in infected cells (as shown in the volcano plots in Fig. 3). Therefore, the rapid growth of the parasite leads to an accumulation of nucleotides and their derivatives and precursors in iRBCs. Of specific interest, infected cells were found to have more than 500-fold enrichment in cytidine, which was not matched by the other nucleotides. However, the Kennedy pathway, which is the major pathway of phosphatidylcholine synthesis in eukaryotes, uses cytidine to convert the precursor phosphocholine to cytidine-5′-diphosphocholine. This step is catalyzed by CTP:phosphocholine cytidylyltransferase (CCT) (46), whose homolog is present in B. divergens (Bdiv_002670). Cytidine-5′-diphosphocholine was also found to be present at more than a 6-fold-higher level in infected RBCs, which suggests that this pathway may be utilized in B. divergens.

In the experiments reported in the next few sections, we analyzed some of these key metabolic pathways in more detail, using inhibitors of major enzymatic reactions in these pathways and studying the effects of their inhibition on various aspects of the parasite cycle. By unveiling the mechanism of action of these inhibitors, we direct attention to potential vulnerable spots in the parasite metabolic network and show how metabolomics analyses can lay the foundation for identifying promising therapeutics, as well as helping to uncover the mechanisms of their action.

### High-resolution microscopy reveals distinct morphological lipid staining patterns of B. divergens-infected cells and validates the lipidomic profile of iRBCs.

**FIG 4:**
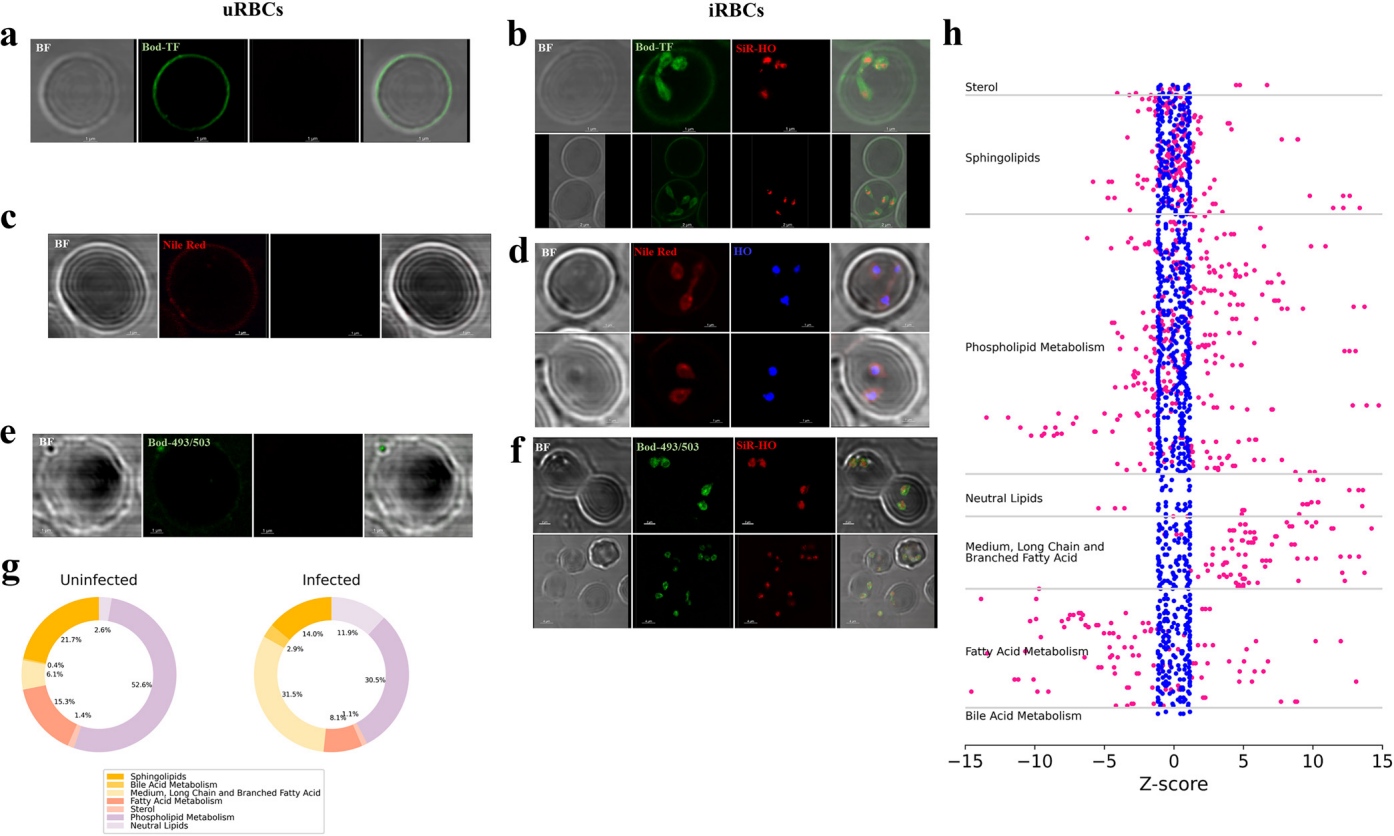
Confocal microscopy reveals distinct morphological patterns of B. divergens-infected cells and validates the lipidomic profile of iRBCs. Uninfected RBCs (uRBCs) and B. divergens-infected RBCs (iRBCs) were stained with lipid dyes and a DNA stain (silicone-rhodamine Hoechst stain [SiR-HO]) and viewed at ×100 magnification in an Abberior STED microscope. Each image is arranged in a panel to show the bright-field (BF) image, the respective dyes (DNA stain was used only in iRBCs), and the merged image. uRBCs are shown in panels a, c, and e, while iRBCs are shown in panels b, d, and f. (a and b) uRBCs and iRBCs were labeled with Bodipy TopFluor (Bod-TF; green), which stains for cholesterol, and SiR-HO (red), which stains for DNA. In uRBCs, the dye stains mainly the membrane of the cell. In iRBCs, the parasite membrane fluoresces brightly, along with some internal staining, while the signal along the periphery of the RBCs is dim. (c and d) uRBCs and iRBCs were stained with Nile red, which stains for polar lipids (red), and Hoechst stain, which stains for DNA (HO; blue). The uRBC membrane stains with Nile red, while iRBCs show stain only around the nucleus and along the membranes of the parasite. (e and f) uRBCs and iRBCs were stained with Bodipy 493/503 (Bod-493/503; green), which stains for neutral lipids, and SiR-HO (red), which stains for DNA. uRBCs and iRBCs have very dim signals, while the parasites fluoresce brightly. (g) The pie charts map the distributions of different lipids in uRBCs and iRBCs. Lipids were divided into subclasses and based on their average intensity in the sample set. The percentages of the lipids changed drastically upon infection, with the major changes reflected in percentages of neutral lipids, sphingolipids, and metabolites belonging to the fatty acid metabolism. (h) The z-score plot was constructed for the subclasses of lipids. Each metabolite from each of the uRBCs (blue) and iRBCs (D4; pink) was plotted. Across all subclasses, the lipid metabolites exhibited different levels in uRBCs and iRBCs (increased or decreased levels as shown by the distribution of pink dots across the positive and negative end of the *x* axis, respectively), suggesting that the parasite confers a distinct lipidomic signature on host RBCs.

Lipids were one of the major classes of metabolites that was the most altered between uRBCs and iRBCs (Fig. 4). To confirm the lipidomic profiles obtained in our global metabolomics analysis, we labeled uninfected and infected red cells with different lipid probes for neutral lipids, polar lipids, and cholesterol, making sure to image all fields at the same settings. The images in Fig. 4b, d, and f show the overall alterations in the lipid staining patterns of infected RBCs containing various pleotropic forms of the parasite compared to those of uRBCs. Of note, in uRBCs, all staining was predominantly localized to the cell plasma membrane (Fig. 4a, c, and e).

Cholesterol staining was visualized using Bodipy TopFluor (Bod-TF), which has been shown to be an excellent tool for visualizing sterol distribution in various cells (47). The plasma membrane of uRBCs is known to contain high levels of free cholesterol, which facilitates the fluidity of the membranes while carefully controlling its permeability. This was corroborated in our microscopy analysis (Fig. 4a), where it was seen that the uRBC membrane was highly fluorescent. However, upon infection, the intensity of the cholesterol staining decreased in the RBC membrane, with the concomitant appearance of the stain in the parasite membranes, and this depletion was proportional to the parasite load (Fig. 4b). While the parasite plasma membrane was fluorescent, accumulation of sterol was also detected within the parasite (Fig. 4b), which may be parasite organellar structures.

Nile red was next used to probe the polar lipid localization in both uRBCs and iRBCs (4c and d). The uRBC membrane showed fluorescence that was weaker than the Bod-TF staining of cholesterol but stronger than the neutral lipid staining (Fig. 4e and f). However, iRBCs showed strong staining with Nile red that was distributed throughout the parasite structure but was stronger in specific areas, which again could be parasite organelles (Fig. 4d). Organellar membranes are known to be rich in cholesterol and phospholipids (48), and further work is needed to ascertain whether the intracellular lipid staining is localized to these membranes.

The neutral lipids, including the acyl glycerides, were revealed to be the most enriched lipids in the infected cells from our metabolomics data (files 4 and 5 in the supplemental material). While our metabolomics platform does not detect triacylglycerides (TAGs) due to their size and hydrophobicity, all the DAGs were changed at high parasitemia, with an FC of 5 or more in iRBCs with respect to their levels in uRBCs. This is consistent with our confocal microscopy analysis using Bodipy-493/50, which due to its nonpolar structure and long-wavelength absorption and fluorescence can be used as a stain for neutral lipids. While uRBC membranes stained only weakly with this dye (Fig. 4e), iRBCs exhibited strong fluorescence within the parasite cytoplasm, specifically staining structures that may be lipid bodies, based on their appearance (Fig. 4f).

Overall, the microscopic analysis confirmed the elevated levels of the major classes of lipids in the Babesia-infected cells and allowed dissection of these quantitative differences into morphological distributions within the iRBCs and the parasites.

To analyze the major changes in lipid classes during infection, we constructed a pie chart based on the percentage intensity of each class of lipids in uRBCs and iRBCs. For each metabolite within the 7 lipid classes, the metabolic intensity obtained from the Metabolon platform was averaged across replicates, and its proportion compared to those of all classes was defined and plotted in the pie chart. Therefore, this gives a semiquantitative contribution of metabolites in each of the lipid classes in both uRBCs and iRBCs. As seen in Fig. 4g, the major changes in the metabolic profile upon infection were from the medium-chain, long-chain, and fatty acids (6.11% in uRBCs versus 31.50% in iRBCs), neutral lipids (2.65% in uRBCs versus 11.86% in iRBCs), and phospholipid metabolism (52.62% in uRBCs versus 30.54% in iRBCs). Therefore, infection not only modulated the levels of lipid metabolites but also caused alterations in the absolute composition of lipids, as has also been shown in Plasmodium (34). The changes in the physical properties of the red cell membrane due to alteration in important membrane lipids by the parasite remain to be investigated in Babesia-infected red cells.

Finally, we constructed a z-score plot for the lipid classes (Fig. 4h). The seven classes of lipids are labeled and separated by horizontal lines. Blue dots represent metabolites from each uRBC’s D4 sample, while pink dots represent metabolites from each iRBC’s D4 sample. The extent of deviation of the pink dots away from the blue dots (centrally located along the *x* axis) is representative of the number of standard deviations by which the absolute value of that metabolite is separated from the mean value. Therefore, the greater the value of the metabolite along the negative or positive *x* axis, the more downregulated or upregulated it is, respectively. Therefore, our results suggest that parasites depend heavily upon host/host-derived lipids and that alteration in lipids is integral to parasite growth and proliferation. In the experiments described in the next couple of sections, we investigated possible drug targets for disruption of specific lipid homeostasis and the effects on parasite survival.

### Loss of cholesterol in the RBC membrane by methyl-β-cyclodextrin (MβCD) treatment does not impact parasite invasion but results in the premature release of the intracellular parasite.

While B. divergens possesses some pathways for both the synthesis and modification of lipids, we found that, like Plasmodium and Toxoplasma, B. divergens lacks the machinery to synthesize cholesterol *de novo* (49, 50). An *in silico* BLASTp analysis for the key enzymes of the cholesterol synthesis pathway against several varied model organisms (Homo sapiens, Arabidopsis thaliana, Mycobacterium tuberculosis, and Schizosaccharomyces pombe) showed that no homologs of this pathway are present in B. divergens. In Fig. S1 in the supplemental material, a simple illustration of the cholesterol biosynthetic pathway is shown, with key enzymes in green. The red cross-marks on these enzymes (HMG CoA synthase, HMG CoA reductase, and lanosterol-14α-demethylase) point out the absence of these enzymes in B. divergens. Thus, these results suggest that, like their related apicomplexans, it is probable that Babesia spp. also scavenge cholesterol from their host cells.

Cholesterol is the most abundant lipid present in the RBC membrane, and thus, this membrane could serve as the source for the parasite’s escalating needs. The metabolomics profile of the infected RBCs revealed an almost 7-fold increase of 7-hydroxycholesterol, which is an oxysterol and, apart from being a biomarker of lipid peroxidation, is the first step in the conversion of cholesterol to bile acids. This allows us to hypothesize that the parasite scavenges cholesterol from its host cell to fulfill this sterol requirement.

**FIG 5:**
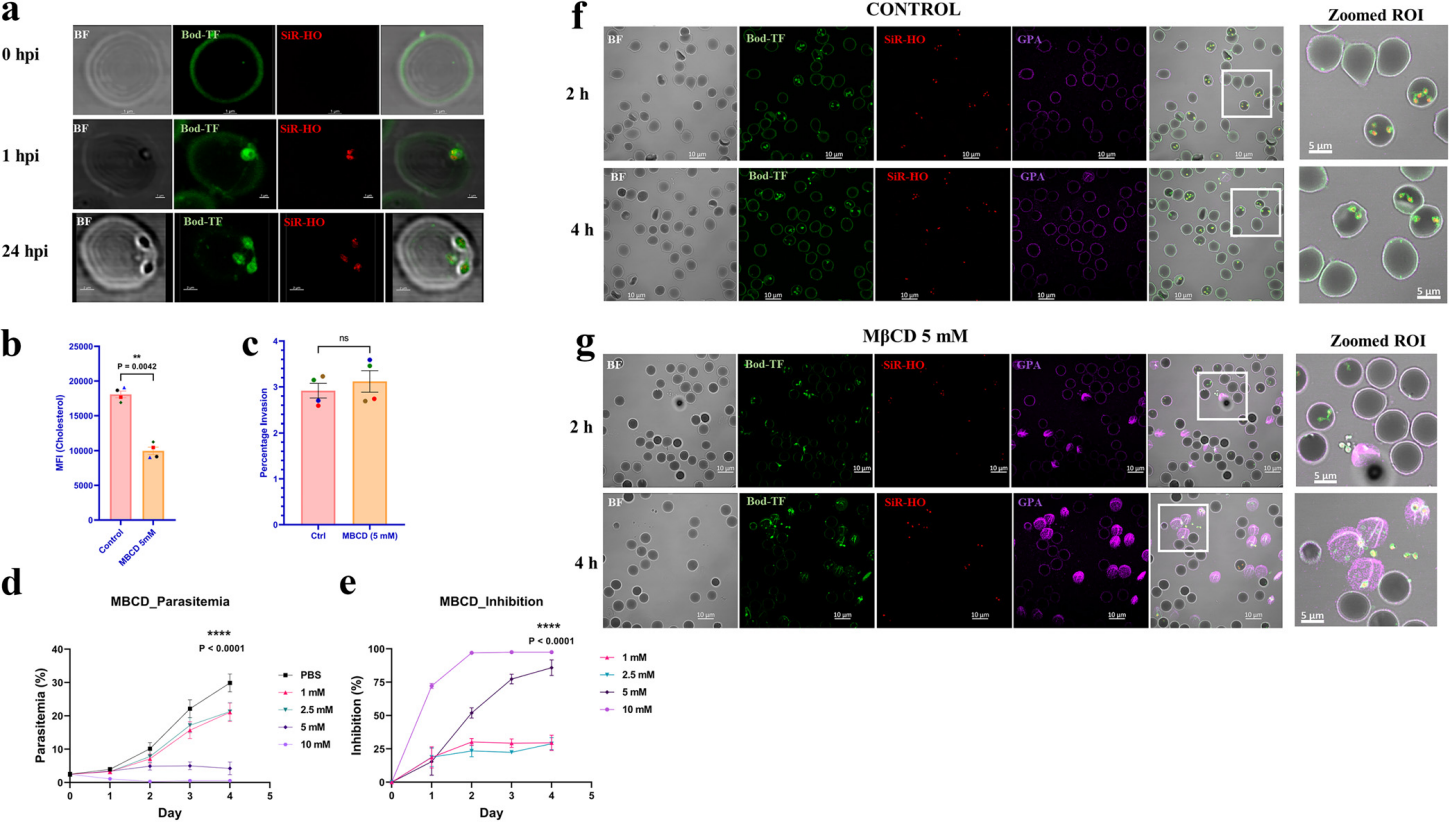
Cholesterol plays an important role in B. divergens infection of RBCs. (a) Uninfected RBCs (uRBCs) were stained with Bodipy TopFluor (Bod-TF) at 0 hpi, and merozoites were allowed to invade these stained cells. Cells were imaged at 1 hpi and 24 hpi. As is evident, the parasites scavenged the labeled cholesterol from their host RBCs at 1 hpi, with a significant signal also present along the membrane of the red blood cell. However, as parasites grew (24 hpi), the signal on the RBC surface decreased drastically, while the parasites had a strong green fluorescent signal around the red DNA dye, along the periphery of the parasite. (b) Cholesterol was measured fluorometrically in control RBCs and in the presence of RBCs treated with the cholesterol inhibitor methyl-β-cyclodextrin (MβCD) (5 mM for 30 min). The cholesterol content in MβCD-treated red cells was significantly depleted, to ~50% of the cholesterol content of control red cells (*P* = 0.0042, *n* = 4). Error bars show standard errors of the means. (c) Purified merozoites were added to control and MβCD (5 mM for 30 min)-treated RBCs, and invasion was monitored using flow cytometry. As is shown, invasion parasitemia was similar under both conditions (*P* = 0.976, *n* = 4). Thus, invasion of B. divergens seems to be unaffected by a reduction in cholesterol content in host RBCs. Error bars show standard errors of the means. ns, not significant. (d and e) Growth inhibition assays (GIAs) were performed with asynchronous ~2% parasitemia cultures mock treated or treated with different concentrations of cholesterol inhibitor, and parasitemia was measured and plotted every 24 h (*n* = 3) (d), along with the percentages of inhibition (e). On day 1 of the assay, parasitemia was 4.6% ± 0.5% (mean value ± SEM) for the control, while the cells treated with MβCD at 1 mM, 2.5 mM, 5 mM, and 10 mM had parasitemias of 3.2% ± 0.4%, 3.2% ± 0.4%, 3.4% ± 0.5%, and 1.1% ± 0.2%, respectively. On day 3, the parasitemia was 24.457% ± 21.191% for the control, while for the increasing drug concentrations, the levels were 15.7% ± 1.4%, 17.183% ± 1.3%, 5.0% ± 0.6%, and 0.5% ± 0.1%, corresponding to percentages of inhibition of ~30% for 1 mM and 2.5 mM and >85% for 5 mM and 10 mM drug concentrations (*P* < 0.0001). (f and g) Control mock-treated or MβCD (5 mM)-treated asynchronous parasites at ~25% parasitemia were incubated for 2 h and 4 h, and at both time points, cells were labeled with DNA dye SiR-HO (red), RBC membrane marker GPA (violet), and cholesterol-staining dye Bod-TF (green). Bright-field images and each of the fluorescent signals are shown, along with a merged image and a merged and zoomed region of interest (ROI) showing the BF image and all the dyes. The ROI from each image is marked in a white square. As is shown, at 2 h and 4 h, RBCs and parasites in control cultures were morphologically preserved, while the cultures treated with 5 mM MβCD had extracellular parasites ejected out of red cells, which resemble ghost cells.

To prove that the parasite in fact scavenges cholesterol from its host, we prelabeled RBCs with Bod-TF (Fig. 5a, green, 0 hpi) and performed an invasion assay by adding freshly purified merozoites. The parasite DNA was stained with silicone-rhodamine Hoechst stain (SiR-HO) (red) and imaged on an STED microscope at 0 hpi, 1 hpi, and 24 hpi. As can be seen in the sequence of events imaged in Fig. 5a, at 1 h postinvasion, the earliest time point assessed in this experiment, there was fluorescence associated with the newly invaded parasite, Thus, the invading merozoite must acquire this cholesterol from the RBC membrane. As the parasite proliferates within the RBC, its requirement for cholesterol is constant, and thus, we followed this sequence of development within the labeled RBC at 1 and 48 h postinvasion (Fig. 5a). Interestingly, even upon binary fission, we could detect the fluorescent cholesterol in the newly formed parasites at all subsequent divisions, albeit at lower intensities. At 24 hpi, while the parasite fluoresced bright green, the fluorescence in the RBC membrane was heavily depleted compared to the fluorescence at the zero-hour time point. These experiments suggest that B. divergens is continuously scavenging cholesterol from the host RBC membrane for its expanding requirements as the intracellular parasite load increases.

To analyze the importance of cholesterol in the biological processes of the parasite, we then used methyl-β-cyclodextrin (MβCD) to deplete the erythrocyte plasma membrane of cholesterol. While MβCD does not fully extract all the cholesterol from the membrane, it has been reported to significantly reduce the content of the sterol in the host RBC membranes (51–55). We used RBCs from four different donors, as cholesterol content is known to vary among individuals (Fig. 5b). To ensure that MβCD treatment (5 mM for 30 min at 37°C) did indeed diminish membrane cholesterol content, we quantified cholesterol using a cholesterol fluorometric kit and solutions with varying cholesterol standards. The results presented in Fig. 5b show that after 5 mM MβCD treatment, all 4 RBC samples exhibited an ~50% decrease in cholesterol content, which was statistically significant (*P* = 0.0042, *n* = 4). Thus, MβCD treatment yielded host cell membranes significantly depleted of cholesterol and suitable to assess the impact of cholesterol loss on parasite processes.

We first examined whether B. divergens could efficiently invade these MβCD-treated RBCs. Previous studies with P. falciparum have shown that the RBC cannot support parasite invasion upon MβCD treatment (51, 53). However, the efficiency of B. divergens invasion using RBCs pretreated with 5 mM MβCD (30 min at 37°C) remained comparable to the efficiency in untreated RBCs using 4 different human blood samples (Fig. 5c). Erythrocyte raft domains are thought to be the areas of the membrane that are cholesterol rich and, thus, targets of MβCD (52). Furthermore, these raft domains are also the points of invasion of Plasmodium (56). This leads us to believe that B. divergens invasion must differ from that of malaria parasites in either the specific areas of the RBC membrane that the parasite requires for successful invasion or the cholesterol richness of those same membranes.

Although invasion was not impacted by MβCD treatment, we examined whether parasites cultured in the presence of the drug would show impaired growth and proliferation. Growth inhibition assays (GIAs) were set up using 0 to 10 mM concentrations of MβCD. As seen by the results in Fig. 5d and e, there was a significant inhibition of parasitemia with increase in drug concentration, and the inhibition was time dependent. Both 5 and 10 mM MβCD exhibited dramatic inhibition of parasite growth, but treatment of cells with 10 mM MβCD also resulted in host RBC lysis. We therefore did not use the 10 mM concentration of MβCD further in our experiments. We confirmed that concentrations of 5 mM MβCD and lower had no impact on RBC integrity and did not cause lysis but showed >75% inhibition by day 4. Concentrations of 1 and 2 mM MβCD were more moderate in their inhibition, and these parasites presented continued culture progression, although at significantly lower parasitemia than the growth rate of the control cultures, and they exhibited a constant 25% inhibition of parasitemia through all 4 days of the assay (Fig. 5d).

To explore the mechanism of this inhibition, we examined Giemsa-stained smears of these MβCD-treated parasite cultures. The drug-treated parasites present a contrasting picture to control cultures. While control cultures contained intra-RBC parasites with only a few extracellular merozoites, drug-treated cultures showed bunches of parasites outside the RBCs. This expulsion of parasites from host RBCs was dose dependent and increased as the time of drug exposure increased. To investigate this inhibition more carefully, a time series of imaging was performed where asynchronous parasite cultures at 25% parasitemia were exposed to 5 mM MβCD and stained with Bod-TF (green) as a marker of cholesterol, SiR-HO (red) as a marker of DNA, and anti-glycophorin A (GPA) antibody (violet) as a marker of the RBC membrane. The images reveal that soon after the introduction of MβCD, cultures began to reveal extracellular parasites, and the host red cell membrane appeared ghost-like, with a disordered GPA stain. In fact, microscopy revealed that the treated red cells seemed to spit out the parasites, leading to the decreased parasitemia in these cultures. These cells grew in number, and at 4 h postexposure, most of the parasites appeared to be extracellular, in contrast to control parasites, which continued their regular intracellular development. Our results echo those obtained in P. falciparum, where a dramatic expulsion of mature-stage parasites occurred upon exposure to MβCD (51). Therefore, our results indicate that, although cholesterol is scavenged by the parasite from the RBC membrane, selective depletion of cholesterol by MβCD did not interfere with parasite invasion. However, the viability of the intracellular parasite was compromised upon treatment of infected cultures with MβCD. Additionally, the mechanism of inhibition by MβCD appears to be like that seen in Plasmodium, where the parasite is forcefully ejected from the infected red cells (51).

### Orlistat is a potent inhibitor of the lipase that degrades acylglycerides and disrupts B. divergens merozoite membrane formation.

The neutral glycerolipids, including several forms of diacyl glycerols (DAGs), were one of the major classes of lipids that were highly expressed in the erythrocytic B. divergens stages. To understand the potential role these lipids play in the asexual erythrocytic cycle of the parasite, we used the inhibitor orlistat, which is an irreversible inhibitor of the lipase that hydrolyzes TAGs and DAGs to a monoglyceride molecule and free fatty acids (57, 58). Orlistat is known to target the human enzyme monoacylglycerol lipase (MGLL) (59), which has the characteristic GXSXG motif, a catalytic triad (serine, histidine, and aspartate), and a cysteine residue adjacent to the active site. In P. falciparum, several serine hydrolases were identified, but it was conclusively shown that PF3D7_1038900 (also called PfMAGLLP) was the target of orlistat (60). An *in silico* search of the B. divergens genome revealed a homolog of PfMAGLLP, namely, *Bdiv_023350c*. As shown in Fig. S2, the lipase homolog in B. divergens has all the conserved domains (the GXSXG motif, critical cysteine residue, and catalytic triad). Thus, we hypothesize that this enzyme is the target of orlistat, although there could be additional targets in B. divergens.

**FIG 6:**
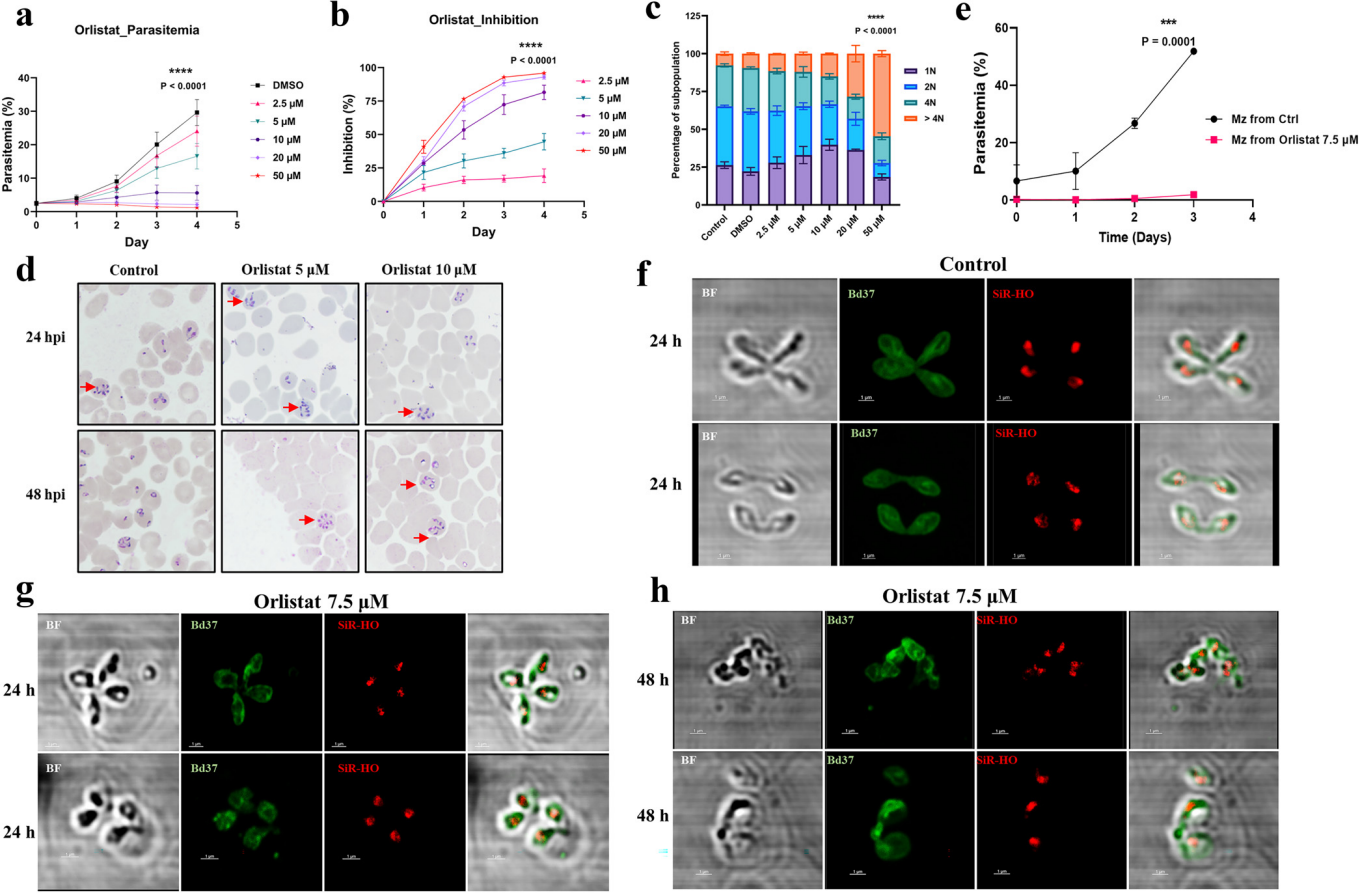
Orlistat potently inhibits the growth of the parasite by disrupting its plasma membrane organization. (a and b) Growth inhibition assays (GIAs) were performed with asynchronous ~2% parasitemia cultures mock treated or treated with different concentrations of orlistat, and parasitemia was measured and plotted every 24 h (*n* = 3) (a), along with percentages of inhibition (b). Parasitemia on day 3 for the control was 24.5% ± 2.2% (mean value ± SEM), while for orlistat-treated cultures, the levels were 16.7% ± 2.0% (2.5 μM), 13.0% ± 1.7% (5 μM), 5.7% ± 1.3% (10 μM), 2.4% ± 0.5% (20 μM), and 1.4% ± 0.15% (50 μM), with statistically significant parasitemia and percentages of inhibition compared to the control (*P* < 0.0001, *n* = 3). (c) The subpopulation structure of parasites (D2 of the GIA) growing under each of these conditions was examined and plotted as percentages of parasites that were 1N, 2N, 4N, and >4N. With increasing drug concentration, the proportion of >4N parasites was significantly increased. While >4N contributed 7.77% ± 0.659% of the subpopulation structure of control parasites, treatment with orlistat resulted in proportions of >4N parasites of 11.5% ± 0.07% (2.5 μM), 12.1% ± 0.6% (5 μM), 15.0% ± 0.2% (10 μM), 28.4% ± 3.1% (20 μM), and 54.6% ± 1.2% (50 μM). (d) Giemsa-stained iRBCs corroborate our findings from the experiment whose results are shown in panel c. At 24 h and 48 h, several iRBCs in orlistat-treated cultures harbored multiple >4N parasites (marked with red arrows), in contrast to the few seen in control iRBCs. (e) To assess the viability of parasites in orlistat-treated cultures, merozoites were purified from control or orlistat (7.5 μM for 24 h)-treated cultures, and an invasion assay was set up with RBCs. While merozoites purified from control cultures invaded normally, the invasion of merozoites purified from the drug-treated culture was ~0.2, and it continued to remain <1% until day 3 postinvasion, while control parasites grew to ~60% (*n* = 2). Error bars show standard errors of the means. (f) Control parasites from the GIA were imaged in the Abberior STED microscope at ×100 magnification and stained with antibody against parasite membrane protein Bd37, as well as an FITC-labeled secondary antibody (green) and the DNA dye SiR-HO (red). As seen, parasites appear morphologically healthy and Bd37 was seen labeling the periphery of the parasite. (g and h) However, for parasites treated with orlistat (7.5 μM) for 24 h (g) and 48 h (h) and stained with Bd37-FITC and SiR-HO, it was clearly evident that the parasites were morphologically defective and the Bd37-FITC was not uniform across the periphery of the parasites, with the effect being time dependent (also shown in Movies S1, S2, and S3 in the supplemental material).

Upon orlistat treatment (7.5 μM for 24 h), we could see an increase in the intensity of the signal of the nonpolar lipid-staining dye (Bodipy 493/503), suggesting that our treatment was targeting TAG hydrolysis. Growth inhibition assays (GIAs) were performed with asynchronous ~2% parasitemia cultures treated with different concentrations of orlistat (2.5, 5. 10, 20, and 50 μM), and parasitemia was measured and plotted every 24 h, along with the percentages of inhibition, as shown in Fig. 6b. As a control, cultures with dimethyl sulfoxide (DMSO) diluent were used for monitoring differential parasite proliferation. Inhibition assays were run for 4 days, with medium changes with the drug carried out every 24 h. As can be seen from the results in Fig. 6a and b, all concentrations of orlistat inhibited parasite growth, and this inhibition was both concentration and time dependent. Significant changes in parasitemia were noted as early as 24 h posttreatment, with the highest concentrations (50 and 20 μM) of orlistat yielding 25 to 35% inhibition and 10 μM orlistat showing ~10% inhibition. The parasitemia on day 3 for the control was 24.5% ± 2.2% (mean value ± standard error of the mean [SEM]), while for orlistat-treated cultures, the values were 16.7% ± 2.0% (2.5 μM), 12.9% ± 1.7% (5 μM), 5.7% ± 1.3% (10 μM). 2.3% ± 0.5% (20 μM), and 1.4% ± 0.2% (50 μM), with statistically significant percentages of growth inhibition compared to the growth of the control (*P* < 0.0001, *n* = 3). As shown, at 10 μM drug treatment for 2 days, parasites were inhibited by ~50%, while higher concentrations of the drug led to an inhibition of >75% on D2. Thus, orlistat was potent in inhibiting parasite growth, implying the importance of these lipases for parasite survival.

To understand the mechanism of this inhibition, we first turned to Giemsa-stained smears of the control and orlistat-treated cultures. As seen in Fig. 6d, while the control cultures exhibited morphologies consistent with a healthy mix of the various pleomorphic parasite forms, (mix of rings, paired-figures and Maltese crosses), the orlistat-treated cultures, even at low drug concentrations (5 and 10 μM) had predominantly infected cells hosting multiple parasites (Fig. 6d). Flow cytometric analysis confirmed this finding, where it was found that orlistat-treated cultures had significantly higher 4N and >4N subpopulations of parasites (Fig. 6c). With an increasing drug concentration, the proportion of >4N parasites was significantly increased. While >4N parasites contribute 7% ± 0.7% of the subpopulation structure of control parasites, their proportions in cultures treated with orlistat were 11.5% ± 0.1% at 2.5 μM, 12.1% ± 0.6% at 5 μM, 15.0% ± 0.3% at 10 μM, 28.4% ± 3.2% at 20 μM, and 54.6% ± 1.2% at 50 μM orlistat. This led us to conclude that while intracellular proliferation continued, inhibition was occurring at the level of merozoite release or merozoite maturation. A rational explanation for this conclusion could be that the formation of free fatty acids from DAGs that help in merozoite membrane formation is inhibited by orlistat. Conversely, the free fatty acids may also play a role in RBC membrane lysis due to their detergent-like properties, and this is impaired by orlistat treatment (61).

To assess the viability and functionality of merozoites from orlistat-treated cultures, we purified merozoites from both control and 7.5 μM orlistat-treated cultures (24h) harvested at the same parasitemia (~25%). Following invasion, parasitemias were assessed at 1h and 24 h postinvasion. As can be seen from the results in Fig. 6e, merozoites from orlistat-treated cultures were unable to successfully invade new host RBCs, and thus, new cycles of invasion could not be initiated. Therefore, these results suggest that ineffective RBC membrane lysis was not responsible for the low parasitemia in drug-treated cultures. Instead, defective merozoite maturation may have been the responsible factor. To assess whether the loss of free fatty acids resulted in defective merozoite membrane genesis, we used Bd37 as a marker to examine the merozoite membranes. Bd37 is the major surface antigen of B. divergens and has been shown to be glycosylphosphatidylinositol (GPI) anchored at the surface of the merozoite (62–64).

Parasites were treated with 7.5 μM orlistat at 20% parasitemia and imaged for Bd37 (green) localization after 24 h of incubation with Orlistat. Parasites were stained with SiR-HO (red) to stain the DNA of the parasite. The images in Fig. 6f to h show the results of STED-based microscopic analysis of orlistat-treated parasite cultures compared to DMSO-treated cultures. While the DMSO-treated parasites exhibited normal parasite membrane structures, as evidenced by the uniform localization of Bd37 around the merozoites (Fig. 6f), orlistat-treated merozoites lacked defined membrane structures, as seen in the discontinuity in Bd37 localization around the daughter merozoites at 24h (Fig. 6g) and even more severely at 48h (Fig. 6h). In the control cultures, the plasma membrane of the merozoites completely enveloped the merozoites, while incomplete ingression of the plasma membrane in daughter merozoites characterized the orlistat-treated parasites. Our results suggest that the drug inhibited merozoite maturation before egress and that TAG and DAG hydrolysis is essential for proper merozoite maturation. To corroborate our two-dimensional (2-D) images from the STED microscopy, Z-stack images were acquired to create a nearly isotropic three-dimensional (3-D) image in Imaris. Parasites stained with Bd37-fluorescein isothiocyanate (FITC; green) and SiR-HO (red) in samples from the DMSO control for 24 h (Movie S1) and 7.5 μM orlistat for 24 h (Movie S2) and 48 h (Movie S3) are shown in the supplemental material.

## DISCUSSION

Metabolomic analyses provide a chemical fingerprint of thousands of metabolites present in cells, allowing us to probe the mechanisms behind both biological processes and disease states. Here, we have shown that the development of Babesia divergens within the human host RBCs modulates the levels of hundreds of compounds from every major metabolic process, including lipid, amino acid, and nucleotide metabolism, energy generation, and cellular redox potential. Our data suggest an intricate interplay between the host RBCs and the parasite for nutrient acquisition and turnover, as well as waste removal. This is the first global metabolomics study of host RBCs infected with B. divergens in an *in vitro* culture, performed at both low and high parasitemia using the metabolomics platform Metabolon. Our analysis revealed that in infection, there are major changes across all biomolecules and major metabolic pathways. More than 600 metabolites were detected, with significant changes in ~150 and ~350 metabolites in low parasitemia (D2) and high parasitemia (D4). As highlighted by the results in Fig. 1 and Fig. 4, lipids were the major class of molecules impacted by parasite growth. The profiles of amino acids and carbohydrates were also shown to be massively altered by parasite growth in human RBCs. As parasite growth continued, there was an increased reliance on energy pathways like glycolysis and the TCA cycle to fuel the rapid proliferation, and these changes were reflected in the metabolome of the infected host cells. Additionally, as expected and as previously shown by us using redox dyes (32), there were significant changes in the redox regulatory pathways in the infected red cells (Fig. 3f).

We further corroborated changes in specific major subclasses of lipids that were modified by the parasite using both growth inhibition assays and high-resolution STED microscopy (Fig. 4, 5, and 6). We investigated two subclasses of lipids: cholesterol, which is the major lipid in the plasma membrane of cells, and acylglycerols. Our experiments revealed that the parasite scavenges all its cholesterol requirements from the host cell (Fig. 5a) and that a disruption in cholesterol scavenging (as seen by treatment with MβCD) is lethal for the parasite, as it is prematurely ejected out of the RBC (Fig. 5d to g). Furthermore, we found that orlistat, which is a lipase inhibitor and prevents the conversion of TAGs to DAGs, stalls the egress of the parasite from the host, leading to a buildup of 4N and >4N parasite population (Fig. 6c and d) and disrupted parasite membrane structure (Fig. 6f to h and Movies S2 and S3). Our findings are in broad agreement with those reported for the related apicomplexan parasite P. falciparum and, expectedly, as they both share a host cell, the human RBC. However, subtle differences were also recorded, as seen when MβCD did not impact B. divergens invasion, in contrast to Plasmodium. Further work is needed to obtain a comprehensive atlas of the similarities and differences in the metabolic networks of these intraerythrocytic parasites.

A limitation of metabolomics of uninfected and infected red cells is the fact that the host RBC also undergoes metabolic modulation when kept in culture. This has been extensively studied in malaria (12, 27), and it has been shown that the RBC metabolism of the donor contributes to time-dependent changes in RBC metabolic activities during *in vitro* culture. In our system, we also observed these changes in uninfected RBCs kept in culture on D2 versus D4. However, it must be noted that the changes due to infection are far greater than the relatively minor changes in uninfected red cells.

**FIG 7:**
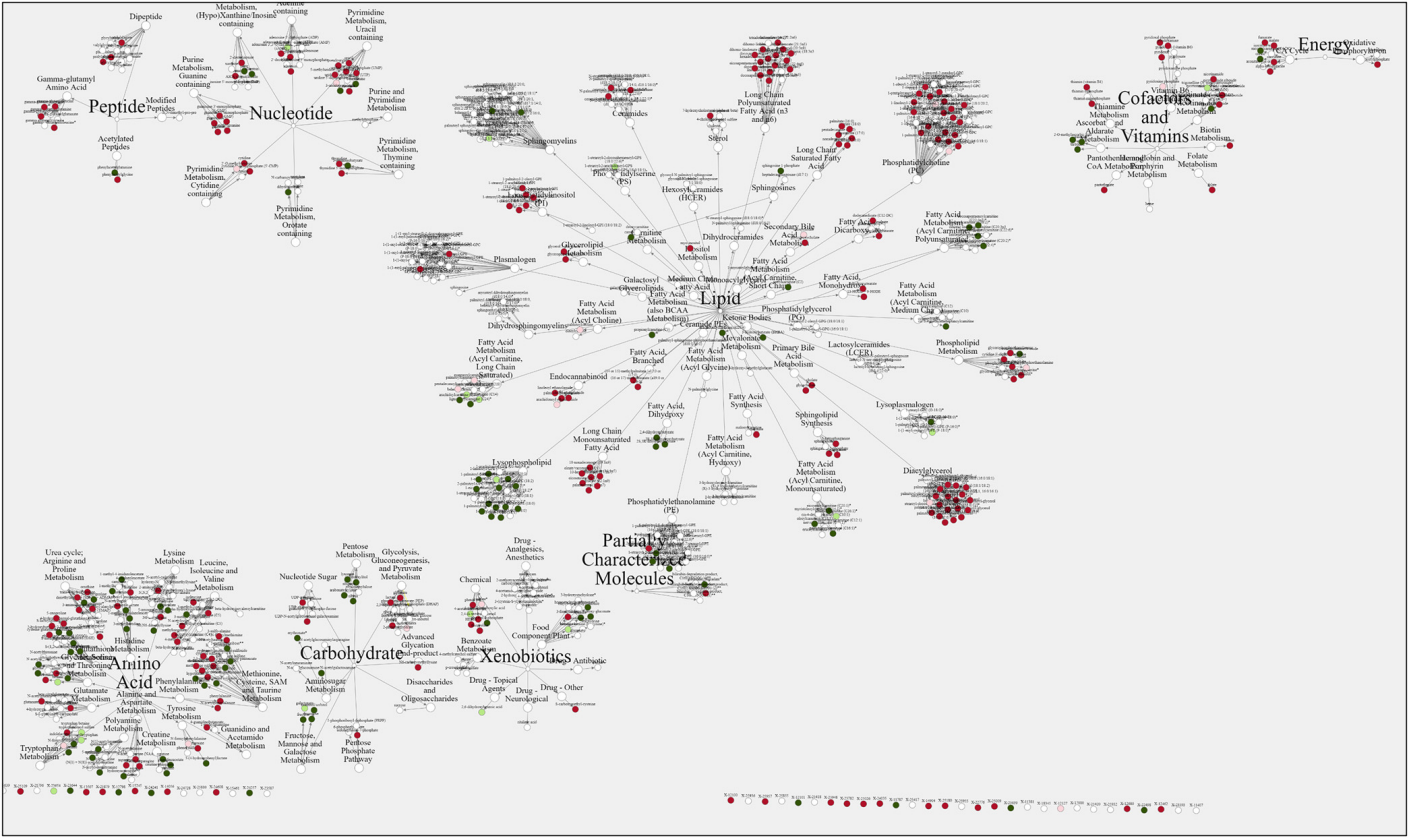
Global metabolomic view of B. divergens-infected RBCs. The image shows the different metabolic pathways uncovered by our analysis classified according to the major biomolecules: lipids, amino acids, carbohydrates, energy production, nucleotides, cofactors and vitamins, xenobiotics, and partially characterized molecules. Under each of these categories, different superpathways are labeled, and metabolites are shown by different colored circular nodes. The color of the circle refers to the fold change of metabolites in iRBCs compared to uRBCs, as follows: white, not significantly affected in the study; light red, increased, with a *P* value between 0.01 and 0.05; dark red, increased, with a *P* value of <0.05; light green, decreased, with a significance value between 0.1 and 0.5; dark green, decreased, with a *P* value of <0.05. Therefore, this study provides the first metabolome of B. divergens-infected RBCs and comprehensively covers modifications of the different metabolic pathways in the infected host cell.

In conclusion, high-resolution metabolomic data were generated and analyzed from uninfected RBCs and B. divergens-infected RBCs at two quantitative infection levels. A global snapshot of changes brought about by B. divergens infection of human RBCs is presented in Fig. 7, with spatial separation by superpathways as described in file 5 in the supplemental material. Those obtained in this study are marked as nodes in green and red, where these colors represent downregulation and upregulation of metabolite abundance, respectively, in iRBCs compared to uRBCs. The metabolites that were unaltered between uRBCs and iRBCs are shown as white nodes. The data in Fig. 7 further emphasize that lipids were the dominant class of biomolecules identified in our study as differentially abundant in iRBCs, as evident from the spread of the lipid web in the figure. Perturbations in amino acid pathways and carbohydrate and nucleotide metabolites are also evident from the overview. Overall, parasite infection of RBCs completely changes the metabolic landscape of the host cells, and the data reported here will deepen our understanding of Babesia biology and its auxotrophic dependence on the host RBC. Furthermore, our study provides a blueprint to further investigate clinically important biochemical pathways in Babesia, as well as related apicomplexans, in greater detail to identify promising chemotherapeutic targets for future investigation.

## MATERIALS AND METHODS

### Ethics statement.

Human blood from healthy volunteer donors was used to culture B. divergens *in vitro*. The blood samples were deidentified and approved for use by the NYBC IRB. All blood donors gave informed written consent for use of their blood for research purposes.

### B. divergens *in vitro* culture.

B. divergens parasites (Bd Rouen 1986 strain) were maintained in human RBCs at 5% hematocrit in complete medium (RPMI 1640 supplemented with 50 μg/mL hypoxanthine, 0.24% [vol/vol] sodium bicarbonate, and 10% human serum) under a low-oxygen atmosphere (5% O_2_, 5% CO_2_, 90% N_2_) at 37°C, as previously described (65). A+ RBCs were collected in 10% citrate-phosphate-dextrose (CPD) and washed thrice with RPMI 1640 medium for the complete removal of plasma and white blood cells.

### Free-merozoite isolation.

A high concentration of viable free merozoites was isolated from unsynchronized cultures at high parasitemia (~35% to 40%) as described previously (25). Briefly, ~12 × 10^9^ infected RBCs were resuspended in 6 mL of fresh complete medium at room temperature and filtered once with a 5-μm filter and twice with 2-μm filters (Versapor membranes) using a 1-mL syringe. Centrifugation (670 × *g* for 5 min) was applied only after the last filtration. The supernatant (suspension of free merozoites) was further spun at 10,000 rpm for 5 min at room temperature. The merozoite pellet was resuspended in 2 mL of complete medium and used for invasion of an equal number of host RBCs. All inoculum preparations were checked for potential contamination with carryover RBCs by light microscopy and flow cytometry.

### Assessment of invasion, development, and egress in various RBCs.

Fresh cultures were seeded with purified merozoite suspension at 20% (vol/vol) of culture volume. Host RBC counts were made to ensure that equal numbers of host cells were taken during the invasion. After 60 min of invasion at 37°C, cultures were centrifuged (670 × *g* for 2 min at room temperature) and the medium replaced with fresh complete medium to remove the lysed RBC components introduced into the culture during the inoculation. At additional time points (24 to 72 h), samples were collected to assess the culture progression and subpopulation dynamics from the perspective of parasite development and egress, as previously described (25). The culture size (parasitemia) and parasite proliferation analysis were carried out by flow cytometry, as described in detail below. Characterization of parasite morphology and development was performed on Giemsa-stained slides using light microscopy.

### Flow cytometry.

Samples were analyzed in 1 mL of cell suspension at a concentration of 1 × 10^7^ infected cells/mL in fresh complete medium without previous fixation or washing procedures, as previously described (66). Samples were analyzed on an LSR Fortessa SORP analyzer (BD Biosciences, San Jose, CA) equipped with a 488-nm blue laser for Vybrant DyeCycle green (1:500) (catalog number V350041; Thermo Fisher) detection (bandpass [BP] 530/30 nm) and a 405-nm violet laser for BV421 mouse anti-human CD235a antibody (1:500; BD-562938) and anti-GPA antibody (BP 450/50 nm) detection. Totals of 50,000 singlets were collected at a low flow rate to achieve the maximum resolution among subpopulations of iRBC and further analyzed with FCS Express Software version 7.12. Appropriate compensations were applied to all experiments. All parameters were processed using log scaling.

### Light microscopy.

Blood smears were fixed with methanol and stained with 20% Giemsa stain (Sigma-Aldrich, St. Louis, MO) for the morphological analysis of parasites. A minimum of 2,000 cells were scanned for assessment of changes in morphology using a Nikon Eclipse E 600 microscope.

### Metabolomics of red cell pellets from uRBCs and iRBCs.

Invasion assays were performed in fresh wild-type cells at 5% hematocrit, parasitemia was measured postinvasion, and cultures were incubated at 37°C in an incubator with 90% N_2_, 5% O_2_, and 5% CO_2_. An equal number of uninfected cells was incubated under identical conditions. At the required time point, 150 μL amounts of the cell pellets were washed thoroughly with 1× phosphate-buffered saline (PBS), flash frozen in a methanol-dry ice bath, and stored in bar-coded tubes at −80°C. Samples were shipped to Metabolon, Inc., and details of the workflow and data analysis, along with statistical parameters used in the study, are provided in file 1 in the supplemental material.

### Membrane cholesterol estimation assays.

Membrane cholesterol was quantified using a fluorometric cholesterol quantitation kit (Sigma-Aldrich) in accordance with the manufacturer’s instructions. Membranes were extracted with a chloroform-isopropanol-IGEPAL CA-630 solution and spun at 13,000 × *g* for 10 min, and the organic phase was transferred to a new tube. Samples were speed vacuum treated for 30 min to remove any residual organic solvents before the lipid pellets were dissolved in cholesterol assay buffer and vortexed until the mixture was homogenous. Fluorescence intensity was measured using a fluorescence plate reader (excitation, 535 nm; emission, 587 nm). A standard curve was graphed, and for the test samples, the concentrations of cholesterol correlating to their respective fluorescence intensities were determined.

### Immunofluorescence assays.

For immunofluorescence assays, the following dilutions of antibodies/dyes were used: Bodipy 493/503 (catalog number D3922; Life Technology) was used at 10 μg/mL (1 mg/mL stock in ethanol), LipidTOX green (catalog number H34475; Life Technology) was used at the recommended 1:200 dilution, Bodipy TopFluor (SKU 810255P; Avanti Polar Lipids) was used at 7.5 μM (stock 1.73 mM in ethanol), silicone-rhodamine Hoechst stain (SiR-HO) (catalog number SC007; Spirochrome) was used at 10 μM (10 mM stock in DMSO), Hoechst 33342 (catalog number PI62249; Life Technology) was used at 0.5 μM, and Nile red (product number 72485; Sigma) was used at 1 μg/mL (1 mg/mL stock in acetone). Glass slides were coated with 0.01% poly-l-lysine overnight and washed thrice with PBS. Parasite culture (200 μL at 5% hematocrit) was added to the slides, and RBCs were allowed to adhere for 30 min. Similar cell concentrations of uRBCs were used for attachment. Following this, RBCs were fixed with 4% paraformaldehyde and 0.0075% glutaraldehyde for 20 min at room temperature, followed by three washes with PBS. Following this, cells were stained with the respective dyes in accordance with manufacturer’s instructions. For the Bd37 experiment, cells were permeabilized with 0.1% Triton X-100 for 5 min postfixation and washed thrice with PBS. Cells were incubated with primary antibody anti-Bd37 antibody (15) at 1:1,000 for 1 h at room temperature. This was followed by 3 washes and incubation with Alexa Fluor 488 donkey anti-rabbit IgG (catalog number 406416; BioLegend) at 1:200.

### Drug treatments.

The following drugs and stock concentrations were used for the experiments described in this manuscript: orlistat (38 mM stock in DMSO) (product number O4139; Sigma) and methyl β-cyclodextrin (MβCD; 320 mM stock in 1× PBS) (product number C4555; Sigma). For drug assays in B. divergens *in vitro* cultures (growth inhibition assays [GIA]), drugs were added to the culture at the required concentration and at ~2% parasitemia. Every 24 h, fresh medium with drug was added. For invasion experiments with MβCD (Fig. 5c), 1 mL of 50% uRBCs was treated with 5 mM MβCD, as previously described (51), for 30 min in incomplete RPMI and washed thrice with PBS. Invasion was performed as described above, and parasitemia was measured using flow cytometry at 1 h postinvasion.

### STED microscopy and image analysis.

Images were acquired in an Abberior Facility Line stimulated emission depletion (STED) microscope at Rockefeller University Bio-Imaging Resource Center, New York, NY, USA. A 100× objective lens was used. The respective lasers were used for FITC (green) and SiR-HO (red; marker for DNA). For each experiment, laser power and accumulation were adjusted. For 2-D images, the pixel size was kept at 15 nm, while for z-stacks, the pixel size was 35 nm and z-steps were set at 50 nm to achieve a nearly isotropic 3-D image. Dwell time was always kept at 10 μs. The .obf images obtained were converted to .ims format and further analyzed in the Imaris software. Imaris 9.9.1 software by Oxford Instruments was used to analyze and process images and videos. A 3-D model was processed using surface rendering, with a surface grain size of 0.025 μm. Animation of the 3-D model was recorded at 20 frames per second with a total of 550 frames and 1,600 by 1,200 Ultra eXtended Graphics array (UXGA) (4:3) resolution. The video clips were saved in the .mp4 format.

### Statistics.

Parasitemia was defined as the total number of infected RBCs (iRBCs) in every 100 RBCs, not taking into consideration the number of parasites present in each cell when measured by flow cytometry. As RBCs do not contain a nucleus, iRBCs and their subpopulations were identified as a function of the presence and number of intraerythrocytic parasites/genome (intraerythrocytic parasite load), where “1N” refers to one genome copy, based on the method previously described (66). Inhibition of culture growth was calculated as the percentage of parasitemia of control cultures. Data reproducibility was ensured by running independent experiments, each in triplicate. Results are represented as the mean values ± standard errors of the means (SEM). Student’s *t* test, one-way analysis of variance (ANOVA), and two-way ANOVA were calculated using GraphPad Prism 9, as applicable. The significance level was set at a *P* value of <0.05.
