# Supplementary material for: Global Metabolomic Profiling of Host Red Blood Cells Infected with Babesia divergens Reveals Novel Antiparasitic Target Pathways
Source: Microbiol Spectr. 2023 Feb 14;11(2):e04688-22. doi: 10.1128/spectrum.04688-22 (PMC10100774; doi:10.1128/spectrum.04688-22)
Supplement: Supplemental file 1 — Supplemental material. Download spectrum.04688-22-s0001.pdf, PDF file, 0.9 MB [file spectrum.04688-22-s0001.pdf]

List of Supplementary Data:

Supplementary Files:

Supplementary File 1. Detailed Metabolon Inc methodology and statistical tools used for acquiring and analysis of the global metabolomics data.

Supplementary File 2. Codes written in RStudio (ggplot2) and Python matplolib for the plots constructed in Fig. 1, 2 and 3.

Supplementary File 3. MetaboAnalyst 5.0 report generated on the Pathway Impact Analysis and Metabolite set Enrichment Analysis (MSEA).

Supplementary File 4. D2 low parasitemia set raw intensity values and fold change between uRBCs and iRBCs along with log_2FC and -log_{10}P-value for metabolites belonging to lipids, nucleotides, amino acids and carbohydrates and energy.

Supplementary File 5. D4 high parasitemia set raw intensity values and fold change between uRBCs and iRBCs along with log_2FC and -log_{10}P-value for metabolites belonging to lipids, nucleotides, amino acids and carbohydrates and energy.

Supplementary Figures:

Figure -S1: *In silico* analysis of key enzymes in cholesterol biosynthesis shows absence of these enzymes.

Figure -S2. Multiple sequence analysis of MAGL from human, *P. falciparum* and *B. divergens.*

Supplementary Videos:

Videos generated in Imaris™ of 3D Z-stacks acquired in Abberior STED microscope using 100X objective lens magnification.

Video S1: Mock DMSO-treated control *B. divergens*.

Video S2: 7.5 µM orlistat-treated *B. divergens* at 24h time-point; and

Video S3: 7.5 µM orlistat-treated *B. divergens* at 48-hr time point.

Supplementary File 1: Detailed Metabolon Inc methodology and statistical tools used for acquiring and analysis of global metabolomics data.

Metabolite Summary and Significantly Altered Biochemicals

The present dataset comprises a total of 673 biochemicals, 627 compounds of known identity (named biochemicals) and 46 compounds of unknown structural identity (unnamed biochemicals). ANOVA contrasts were used to identify biochemicals that differed significantly between experimental groups.

An estimate of the false discovery rate (q-value) is calculated to take into account the multiple comparisons that normally occur in metabolomic-based studies. For example, when analyzing 200 compounds, we would expect to see about 10 compounds meeting the $p \leq 0.05$ cut-off by random chance. The q-value describes the false discovery rate; a low q-value ($q<0.10$) is an indication of high confidence in a result. While a higher q-value indicates diminished confidence, it does not necessarily rule out the significance of a result. Other lines of evidence may be taken into consideration when determining whether a result merits further scrutiny. Such evidence may include a) significance in another dimension of the study, b) inclusion in a common pathway with a highly significant compound, or c) residing in a similar functional biochemical family with other significant compounds. Refer to the Appendix for general definitions and further descriptions of false discovery rate and other statistical tests used at Metabolon.

Data Quality: Instrument and Process Variability

**Quality Control Sample**	**Median RSD**
	**RBC**
Internal Standards	3%
Endogenous Biochemicals	6%

Instrument variability was determined by calculating the median relative standard deviation (RSD) for the internal standards that were added to each sample prior to injection into the mass spectrometers. Overall process variability was determined by calculating the median RSD for all endogenous metabolites (i.e., non-instrument standards) present in the MTRX7 technical replicates. Values for instrument and process variability meet Metabolon's acceptance criteria as shown in the table above.

Metabolon Platform

Sample Accessioning: Following receipt, samples were inventoried and immediately stored at -80°C. Each sample received was accessioned into the Metabolon LIMS system and was assigned by the LIMS a unique identifier that was associated with the original source identifier only. This identifier was used to track all sample handling, tasks, results, etc. The samples (and all derived aliquots) were tracked by the LIMS system. All portions of any sample were automatically assigned their own unique identifiers by the

LIMS when a new task was created; the relationship of these samples was also tracked. All samples were maintained at -80ºC until processed.

Sample Preparation: Samples were prepared using the automated MicroLab STAR® system from Hamilton Company. Several recovery standards were added prior to the first step in the extraction process for QC purposes. To remove protein, dissociate small molecules bound to protein or trapped in the precipitated protein matrix, and to recover chemically diverse metabolites, proteins were precipitated with methanol under vigorous shaking for 2 min (Glen Mills GenoGrinder 2000) followed by centrifugation. The resulting extract was divided into five fractions: two for analysis by two separate reverse phase (RP)/UPLC-MS/MS methods with positive ion mode electrospray ionization (ESI), one for analysis by RP/UPLC-MS/MS with negative ion mode ESI, one for analysis by HILIC/UPLC-MS/MS with negative ion mode ESI, and one sample was reserved for backup. Samples were placed briefly on a TurboVap® (Zymark) to remove the organic solvent. The sample extracts were stored overnight under nitrogen before preparation for analysis.

QA/QC: Several types of controls were analyzed in concert with the experimental samples: a pooled matrix sample generated by taking a small volume of each experimental sample (or alternatively, use of a pool of well-characterized human plasma) served as a technical replicate throughout the data set; extracted water samples served as process blanks; and a cocktail of QC standards that were carefully chosen not to interfere with the measurement of endogenous compounds were spiked into every analyzed sample, allowed instrument performance monitoring and aided chromatographic alignment. Tables shown below describe these QC samples and standards. Instrument variability was determined by calculating the median relative standard deviation (RSD) for the standards that were added to each sample prior to injection into the mass spectrometers. Overall process variability was determined by calculating the median RSD for all endogenous metabolites (i.e., non-instrument standards) present in 100% of the pooled matrix samples. Experimental samples were randomized across the platform run with QC samples spaced evenly among the injections, as outlined below.

Description of Metabolon QC Samples

Type	Description	Purpose
MTRX	Large pool of human plasma maintained by Metabolon that has been characterized extensively.	Assure that all aspects of the Metabolon process are operating within specifications.
CMTRX	Pool created by taking a small aliquot from every customer sample.	Assess the effect of a non-plasma matrix on the Metabolon process and distinguish biological variability from process variability.
PRCS	Aliquot of ultra-pure water	Process Blank used to assess the contribution to compound signals from the process.
SOLV	Aliquot of solvents used in extraction.	Solvent Blank used to segregate contamination sources in the extraction.

Metabolon QC Standards

Type	Description	Purpose
RS	Recovery Standard	Assess variability and verify performance of extraction and instrumentation.
IS	Internal Standard	Assess variability and performance of instrument.

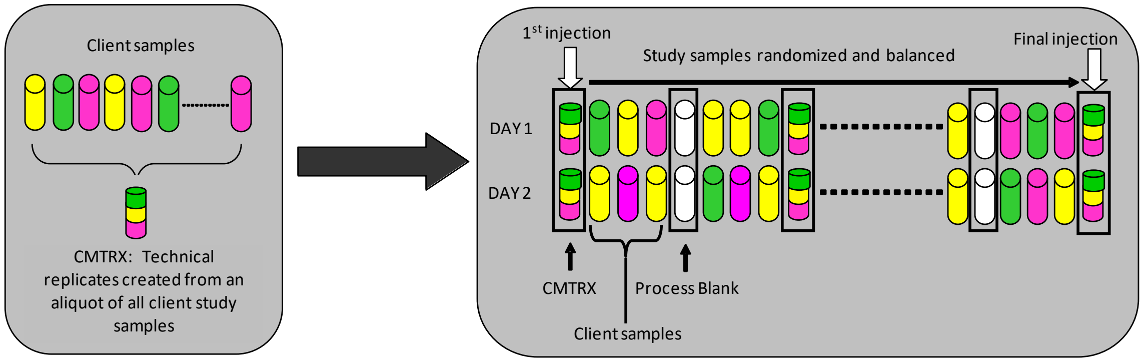

Preparation of client-specific technical replicates. A small aliquot of each client sample (colored cylinders) is pooled to create a CMTRX technical replicate sample (multi-colored cylinder), which is then injected periodically throughout the platform run. Variability among consistently detected biochemicals can be used to calculate an estimate of overall process and platform variability.

Ultrahigh Performance Liquid Chromatography-Tandem Mass Spectroscopy (UPLC-MS/MS): All methods utilized a Waters ACQUITY ultra-performance liquid chromatography (UPLC) and a Thermo Scientific Q-Exactive high resolution/accurate mass spectrometer interfaced with a heated electrospray ionization (HESI-II) source and Orbitrap mass analyzer operated at 35,000 mass resolution. The sample extract was dried then reconstituted in solvents compatible to each of the four methods. Each reconstitution solvent contained a series of standards at fixed concentrations to ensure injection and chromatographic consistency. One aliquot was analyzed using acidic positive ion conditions, chromatographically optimized for more hydrophilic compounds. In this method, the extract was gradient eluted from a C18 column (Waters UPLC BEH C18-2.1x100 mm, 1.7 µm) using water and methanol, containing 0.05% perfluoropentanoic acid (PFPA) and 0.1% formic acid (FA). Another aliquot was also analyzed using acidic positive ion conditions, however it was chromatographically optimized for more hydrophobic compounds. In this method, the extract was gradient eluted from the same afore mentioned C18 column using methanol, acetonitrile, water, 0.05% PFPA and 0.01% FA and was operated at an overall higher organic content. Another aliquot was analyzed using basic negative ion optimized conditions using a separate dedicated C18 column. The basic extracts were gradient eluted from the column using methanol and water, however with 6.5mM Ammonium Bicarbonate at pH 8. The fourth aliquot was analyzed via negative ionization following elution from a HILIC column (Waters UPLC BEH Amide 2.1x150 mm, 1.7 µm) using a gradient consisting of water and acetonitrile with 10mM Ammonium Formate, pH 10.8. The MS analysis alternated between MS and data-dependent MS^n scans using dynamic exclusion. The scan range varied slighted between methods but covered 70-1000 m/z. Raw data files are archived and extracted as described below.

Bioinformatics: The informatics system consisted of four major components, the Laboratory Information Management System (LIMS), the data extraction and peak-identification software, data processing tools for QC and compound identification, and a collection of information interpretation and visualization tools for use by data analysts. The hardware and software foundations for these informatics components were the LAN backbone, and a database server running Oracle 10.2.0.1 Enterprise Edition.

LIMS: The purpose of the Metabolon LIMS system was to enable fully auditable laboratory automation through a secure, easy to use, and highly specialized system. The scope of the Metabolon LIMS system encompasses sample accessioning, sample preparation and instrumental analysis and reporting and advanced data analysis. All of the subsequent software systems are grounded in the LIMS data structures. It has been modified to leverage and interface with the in-house information extraction and data visualization systems, as well as third party instrumentation and data analysis software.

Data Extraction and Compound Identification: Raw data was extracted, peak-identified and QC processed using Metabolon's hardware and software. These systems are built on a web-service platform utilizing Microsoft's .NET technologies, which run on high-performance application servers and fiber-channel storage arrays in clusters to provide active failover and load-balancing. Compounds were identified by comparison to library entries of purified standards or recurrent unknown entities. Metabolon maintains a library based on authenticated standards that contains the retention time/index (RI), mass to charge ratio (*m/z)*, and chromatographic data (including MS/MS spectral data) on all molecules present in the library. Furthermore, biochemical identifications are based on three criteria: retention index within a narrow RI window of the proposed identification, accurate mass match to the library +/- 10 ppm, and the MS/MS forward and reverse scores between the experimental data and authentic standards. The MS/MS scores are based on a comparison of the ions present in the experimental spectrum to the ions present in the library

spectrum. While there may be similarities between these molecules based on one of these factors, the use of all three data points can be utilized to distinguish and differentiate biochemicals. More than 3300 commercially available purified standard compounds have been acquired and registered into LIMS for analysis on all platforms for determination of their analytical characteristics. Additional mass spectral entries have been created for structurally unnamed biochemicals, which have been identified by virtue of their recurrent nature (both chromatographic and mass spectral). These compounds have the potential to be identified by future acquisition of a matching purified standard or by classical structural analysis.

Curation: A variety of curation procedures were carried out to ensure that a high quality data set was made available for statistical analysis and data interpretation. The QC and curation processes were designed to ensure accurate and consistent identification of true chemical entities, and to remove those representing system artifacts, mis-assignments, and background noise. Metabolon data analysts use proprietary visualization and interpretation software to confirm the consistency of peak identification among the various samples. Library matches for each compound were checked for each sample and corrected if necessary.

Metabolite Quantification and Data Normalization: Peaks were quantified using area-under-the-curve. For studies spanning multiple days, a data normalization step was performed to correct variation resulting from instrument inter-day tuning differences. Essentially, each compound was corrected in run-day blocks by registering the medians to equal one (1.00) and normalizing each data point proportionately (termed the "block correction"; as shown below). For studies that did not require more than one day of analysis, no normalization is necessary, other than for purposes of data visualization. In certain instances, biochemical data may have been normalized to an additional factor (e.g., cell counts, total protein as determined by Bradford assay, osmolality, etc.) to account for differences in metabolite levels due to differences in the amount of material present in each sample.

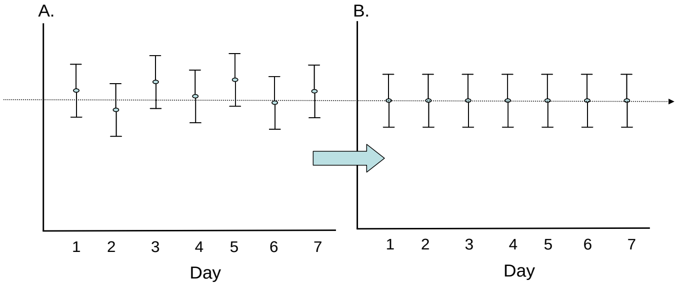

Visualization of data normalization steps for a multiday platform run.

Statistical Methods and Terminology

Statistical Calculations: For many studies, two types of statistical analysis are usually performed: (1) significance tests and (2) classification analysis. Standard statistical analyses are performed in ArrayStudio/Jupyter Notebook. Below are examples of frequently employed significance tests and classification methods followed by a discussion of p- and q-value significance thresholds.

1. **Two-way ANOVA**
   ANOVA stands for analysis of variance. For ANOVA, it is assumed that all populations have the same variances. For a two-way ANOVA, three statistical tests are typically performed: the main effect of each factor and the interaction.

2. **p-values**
   For statistical significance testing, p-values are given. The lower the p-value, the more evidence we have that the null hypothesis (typically that two population means are equal) is not true. If "statistical significance" is declared for p-values less than 0.05, then 5% of the time we incorrectly conclude the means are different, when they are the same.

   The p-value is the probability that the test statistic is at least as extreme as observed in this experiment given that the null hypothesis is true. Hence, the more extreme the statistic, the lower the p-value and the more evidence the data gives against the null hypothesis.

3. **q-values**
   The level of 0.05 is the false positive rate when there is one test. However, for a large number of tests we need to account for false positives. There are different methods to correct for multiple testing. The oldest methods are family-wise error rate adjustments (Bonferroni, Tukey, etc.), but these tend to be extremely conservative for a very large number of tests. With gene arrays, using the False Discovery Rate (FDR) is more common. The family-wise error rate adjustments give one a high degree of confidence that there are zero false discoveries. However, with FDR methods, one can allow for a small number of false discoveries. The FDR for a given set of compounds can be estimated using the q-value.

Supplementary File 2: Codes written in RStudio (ggplot2) and Python matplolib for the plots constructed in Fig. 1, 2 and 3.

VOLCANO PLOT IN R

```
library("readxl")
library("ggrepel")


FILE="/Users/Documents/human/NYBC-01-21VW+ CO DATA TABLES.XLSX"
my_data <- read_excel(FILE, sheet = 3)
my_data_cleaned = my_data
my_data_cleaned$diffexpressed <- "FC</>1_NOSIG"
my_data_cleaned$diffexpressed[my_data_cleaned$Log2FoldChange >= 1.0 & my_data_cleaned$Log2FoldChange < 5.0 & my_data_cleaned$`-log10Pvalue` > 1.3] <- "UP_SIG"
my_data_cleaned$diffexpressed[my_data_cleaned$Log2FoldChange >= 5.0 & my_data_cleaned$`-log10Pvalue` > 1.3] <- "FC >5_SIG"
my_data_cleaned$diffexpressed[my_data_cleaned$Log2FoldChange >= 1.0 & my_data_cleaned$`-log10Pvalue` <= 1.3] <- "UP_NOSIG"
my_data_cleaned$diffexpressed[my_data_cleaned$Log2FoldChange <= -1.0 & my_data_cleaned$Log2FoldChange > -5.0 & my_data_cleaned$`-log10Pvalue` > 1.3] <- "DOWN_SIG"
my_data_cleaned$diffexpressed[my_data_cleaned$Log2FoldChange <= -5.0 & my_data_cleaned$`-log10Pvalue` > 1.3] <- "FC <5_SIG"
my_data_cleaned$diffexpressed[my_data_cleaned$Log2FoldChange <= -1.0 & my_data_cleaned$`-log10Pvalue` <= 1.3] <- "DOWN_NOSIG"
my_data_cleaned$diffexpressed[my_data_cleaned$Log2FoldChange > -1.0 & my_data_cleaned$Log2FoldChange < 1.0 & my_data_cleaned$`-log10Pvalue` > 1.3] <- "FC </> 1_SIG"
my_data_cleaned$metalabels <- ""
my_data_cleaned$metalabels <- ifelse(my_data_cleaned$Log2FoldChange <= -5.0 & my_data_cleaned$`-log10Pvalue` > 1.3 | my_data_cleaned$Log2FoldChange >= 5.0 & my_data_cleaned$`-log10Pvalue` > 1.3, TRUE, FALSE)
mycolors <- c("coral", "green4", "lightgreen", "gray", "black", "pink", "red", "maroon")
names(mycolors) <- c("FC <5_SIG", "DOWN_SIG", "DOWN_NOSIG", "FC</>1_NOSIG", "FC </> 1_SIG", "UP_NOSIG", "UP_SIG", "FC >5_SIG")
myshape <- c(15, 16, 16, 16, 16, 16, 16, 17)
```

```
names(myshape) <- c("FC <5_SIG", "DOWN_SIG", "DOWN_NOSIG", "FC</>1_NOSIG", "FC </> 1_SIG", "UP_NOSIG", "UP_SIG", "FC >5_SIG")

ggplot(data=my_data_cleaned, aes(x=Log2FoldChange, y=`-log10Pvalue`, label=`Biochemical Name`, col=diffexpressed)) +

  geom_point(aes(shape=diffexpressed), size=2.0) +

  #xlim(-7, 7)# x axis limits

  theme_minimal()  +

  coord_cartesian(clip = "off")+

  geom_text_repel(

    label = ifelse(my_data_cleaned$metalabels, my_data_cleaned$`Biochemical Name`, ""),

    size = 2.2,

    force_pull    = 10, # do not pull text toward the point at (0,0)

    max.time      = 0.5,

    max.iter      = 1e5,

    max.overlaps  = Inf,

    segment.color = NA,

    point.padding = NA,

    point.size = 2

  ) +

  scale_color_manual(values=mycolors) +

  scale_shape_manual(values=myshape) +

  #scale_x_continuous(breaks=seq(0,40,5))

  #scale_x_continuous(name = Log2FoldChange, limits = c(0,10))+

  #scale_y_continuous(name = -log10Pvalue, limits = c(0,10)) +

  geom_vline(xintercept=c(-1.0, 1.0), col="gray76", linetype="longdash", size=1.1) +

  geom_hline(yintercept=-log10(0.05), col="gray76", linetype="longdash", size=1.1) +

  ggtitle("iAA/uAA_high parasitemia") +

  theme(

    plot.title=element_text(family='', face='bold', colour='black', size=20, hjust=0.45, vjust=0.0)+

      png("J:/Bloodborne Parasites/Metabolomics Paper/png files of volcanoplots/iAA/uAA_highparasitemia.png")

  )
```

```
ggsave(
  "/Users/Documents/check.png",
  plot = last_plot(),
  device = NULL,
  path = NULL,
  scale = 1,
  width = NA,
  height = NA,
  units = c("in", "cm", "mm", "px"),
  dpi = 600,
  limitsize = TRUE,
  bg = NULL,
)
```

Z Score plot in PYTHON

```
import math
import pandas as pd
import numpy as np
from matplotlib import pyplot as plt
MAIN_DATA_FILE="/Users/Documents/human/NYBC-01-21VW+ CO DATA TABLES.XLSX"
# SHEET_NAME="Param_bradford_mgml-norm Data"
SHEET_NAME="Log Transformed Data"
PATHWAY_SHEET_NAME="Chemical Annotation"
UNINFECTED_WILD_TYPE_SAMPLES=[
    "NYBC-00142",
    "NYBC-00143",
    "NYBC-00144",
]
INFECTED_WILD_TYPE_SAMPLES=[
    "NYBC-00145",
    "NYBC-00146",
    "NYBC-00147",
```

```
]
df = pd.read_excel(io=MAIN_DATA_FILE, sheet_name=SHEET_NAME)
df_uninfected = df[df['PARENT_SAMPLE_NAME'].isin(UNINFECTED_WILD_TYPE_SAMPLES)]
df_infected = df[df['PARENT_SAMPLE_NAME'].isin(INFECTED_WILD_TYPE_SAMPLES)]

column_indexer = df.columns != 'PARENT_SAMPLE_NAME'
mean = df_uninfected.loc[:, column_indexer].mean()
std = df_uninfected.loc[:, column_indexer].std()
df_uninfected.loc[:, column_indexer] = (df_uninfected.loc[:, column_indexer] - mean) / (std)
df_infected.loc[:, column_indexer] = (df_infected.loc[:, column_indexer] - mean) / (std)
pathway_mapping_df = pd.read_excel(io=MAIN_DATA_FILE,
sheet_name=PATHWAY_SHEET_NAME)
pathway_mappings = []
pathway_separation = []
for row in pathway_mapping_df.iterrows():
    if not isinstance(row[1]['SUPER_PATHWAY'], str):
        if math.isnan(row[1]['SUPER_PATHWAY']):
            continue
    if row[1]['SUPER_PATHWAY'].strip() == 'Partially Characterized Molecules':
        continue
    pathway_mappings.append((row[1]['CHEM_ID'], row[1]['SUPER_PATHWAY']))
pathway_mappings = sorted(pathway_mappings, key=lambda x: x[1])
previour_pathway = pathway_mappings[0][1]
pathway_names = []
pathway_names.append(previour_pathway)
pathway_names_y_locations = []
previous_index = 0
for index, pathway in enumerate(pathway_mappings[1:]):
    if pathway[1] != previour_pathway:
        pathway_separation.append(index)
        pathway_names_y_locations.append((index + previous_index)//2)
        previour_pathway = pathway[1]
```

```
        pathway_names.append(previour_pathway)
        previous_index = index
pathway_names_y_locations.append((index + previous_index)//2)

pathway_mappings_chemids = [x[0] for x in pathway_mappings]

infected_datapoints = []
for _, metabolite_id in enumerate(df_infected):
    if metabolite_id == 'PARENT_SAMPLE_NAME':
        continue
    if metabolite_id not in pathway_mappings_chemids:
        print(metabolite_id)
        continue
    for val in df_infected[metabolite_id].values.tolist():
        infected_datapoints.append((pathway_mappings_chemids.index(metabolite_id), metabolite_id, val))
uninfected_datapoints = []
for metabolite_index, metabolite_id in enumerate(df_uninfected):
    if metabolite_id == 'PARENT_SAMPLE_NAME':
        continue
    if metabolite_id not in pathway_mappings_chemids:
        continue
    for val in df_uninfected[metabolite_id].values.tolist():
        uninfected_datapoints.append((pathway_mappings_chemids.index(metabolite_id), metabolite_id,
val))
# plt.figure(figsize=(6, 10), dpi=100)
fig, ax = plt.subplots()
fig.set_size_inches(6, 10)
ax.spines['top'].set_visible(False)
ax.spines['right'].set_visible(False)
# ax.spines['bottom'].set_visible(False)
ax.spines['left'].set_visible(False)
ax.get_yaxis().set_visible(False)
```

```
plt.xticks(fontsize=14)
plt.scatter(
    x=[val[2] for val in infected_datapoints],
    y=[val[0] for val in infected_datapoints],
    c='deeppink',
    s=7,
)
for pathway in pathway_separation:
    plt.axhline(y=pathway, color='lightgray', linestyle='-')
for pathway_name, pathway_names_y_location in zip(pathway_names, pathway_names_y_locations):
    fontsize = 8
    #  if pathway_name == 'Energy' else 10
    ax.text(-15, pathway_names_y_location - 3, pathway_name, fontsize=fontsize)
# 100010917
plt.scatter(
    x=[val[2] for val in uninfected_datapoints],
    y=[val[0] for val in uninfected_datapoints],
    c='blue',
    s=7,
)
plt.xlim(-15, 15)
plt.xlabel('Z-score', fontsize=15)
# plt.ylim(-2, 2)
# plt.rc('ytick', labelsize=SMALL_SIZE)    # fontsize of the tick labels
# plt.rc('legend', fontsize=SMALL_SIZE)    # legend fontsize
# plt.rc('figure', titlesize=BIGGER_SIZE)  # fontsize of the figure title
suffix = SHEET_NAME.replace(' ', '_').lower()
plt.savefig(f'zscore_{suffix}.png', dpi=600)
```

PCA PLOT and PCA LOADING PLOT IN PYTHON

```
import math
```

```
import pandas as pd
import numpy as np
from matplotlib import pyplot as plt
from sklearn.decomposition import PCA
from matplotlib.patches import Ellipse
import matplotlib.transforms as transforms
from adjustText import adjust_text
MAIN_DATA_FILE="/Users/Documents/human/NYBC-01-21VW+ CO DATA TABLES.XLSX"
# SHEET_NAME="Param_bradford_mgml-norm Data"
SHEET_NAME="Log Transformed Data"
PATHWAY_SHEET_NAME="Chemical Annotation"
UNINFECTED_WILD_TYPE_SAMPLES=[
    "NYBC-00142",
    "NYBC-00143",
    "NYBC-00144",
]
INFECTED_WILD_TYPE_SAMPLES=[
    "NYBC-00145",
    "NYBC-00146",
    "NYBC-00147",
]
def confidence_ellipse(x, y, ax, n_std_x=3.0, n_std_y=3.0, facecolor='none', **kwargs):
    """
Create a plot of the covariance confidence ellipse of *x* and *y*.
    Parameters
    ----------
    x, y : array-like, shape (n, )
        Input data.
    ax : matplotlib.axes.Axes
        The axes object to draw the ellipse into.
    n_std : float
```

```
    The number of standard deviations to determine the ellipse's radiuses.
**kwargs
    Forwarded to `~matplotlib.patches.Ellipse`
Returns
-------
matplotlib.patches.Ellipse
"""
if x.size != y.size:
    raise ValueError("x and y must be the same size")
cov = np.cov(x, y)
pearson = cov[0, 1]/np.sqrt(cov[0, 0] * cov[1, 1])
# Using a special case to obtain the eigenvalues of this
# two-dimensionl dataset.
ell_radius_x = np.sqrt(1 + pearson)
ell_radius_y = np.sqrt(1 - pearson)
ellipse = Ellipse((0, 0), width=ell_radius_x * 2, height=ell_radius_y * 2,
                  facecolor=facecolor, **kwargs)
# Calculating the stdandard deviation of x from
# the squareroot of the variance and multiplying
# with the given number of standard deviations.
scale_x = np.sqrt(cov[0, 0]) * n_std_x
mean_x = np.mean(x)
# calculating the stdandard deviation of y ...
scale_y = np.sqrt(cov[1, 1]) * n_std_y
mean_y = np.mean(y)
transf = transforms.Affine2D() \
    .rotate_deg(45) \
    .scale(scale_x, scale_y) \
    .translate(mean_x, mean_y)
ellipse.set_transform(transf + ax.transData)
return ax.add_patch(ellipse)
```

```
labels = UNINFECTED_WILD_TYPE_SAMPLES + INFECTED_WILD_TYPE_SAMPLES
colors = ['blue'] * 3 + ['deeppink'] * 3
df = pd.read_excel(io=MAIN_DATA_FILE, sheet_name=SHEET_NAME)
df_uninfected = df[df['PARENT_SAMPLE_NAME'].isin(UNINFECTED_WILD_TYPE_SAMPLES)]
df_infected = df[df['PARENT_SAMPLE_NAME'].isin(INFECTED_WILD_TYPE_SAMPLES)]

column_indexer = df.columns != 'PARENT_SAMPLE_NAME'
pca = PCA(n_components=2)
np_uninfected = np.array(df_uninfected.loc[:, column_indexer])
np_infected = np.array(df_infected.loc[:, column_indexer])
all_samples = np.vstack([np_uninfected, np_infected])
all_samples_transformed = pca.fit_transform(all_samples)
print(all_samples_transformed)
fig, ax = plt.subplots()
ax.scatter(
    all_samples_transformed[:3,0],
    all_samples_transformed[:3,1],
    c=['blue'] * 3
)
ax.scatter(
    all_samples_transformed[3:,0],
    all_samples_transformed[3:,1],
    c=['deeppink'] * 3,
)
plt.legend(['Uninfected', 'Infected'])
plt.xlim(-30, 30)
plt.ylim(-25, 25)
confidence_ellipse(all_samples_transformed[:3, 0], all_samples_transformed[:3, 1], ax, n_std_x=3.0,
n_std_y=3.0, facecolor='blue', alpha=0.2, edgecolor='none', zorder=0)
confidence_ellipse(all_samples_transformed[3:, 0], all_samples_transformed[3:, 1], ax, n_std_x=3.5,
n_std_y=1.6, facecolor='deeppink', alpha=0.2, edgecolor='none', zorder=0)
first_pc_explained_variance = str(round(pca.explained_variance_ratio_[0] * 100,2))
```

```
second_pc_explained_variance = str(round(pca.explained_variance_ratio_[1] * 100,2))
plt.xlabel(f'PC1 ({first_pc_explained_variance}%)', fontsize=12)
plt.ylabel(f'PC2 ({second_pc_explained_variance}%)', fontsize=12)
plt.axhline(y=0, color='black', linestyle=(0, (1, 4)), linewidth=1)
plt.axvline(x=0, color='black', linestyle=(0, (1, 4)), linewidth=1)

suffix = SHEET_NAME.replace(' ', '_').lower()
plt.savefig(f'pca_{suffix}.png', dpi=600)
# Loading Plot
pathway_mapping_df = pd.read_excel(io=MAIN_DATA_FILE,
sheet_name=PATHWAY_SHEET_NAME)
pathway_mappings = {}
chemid_to_chemname = {}
chemids = []
for row in pathway_mapping_df.iterrows():
    if not isinstance(row[1]['SUPER_PATHWAY'], str):
        if math.isnan(row[1]['SUPER_PATHWAY']):
            row[1]['SUPER_PATHWAY'] = 'Uncharacterized Molecules'
    chemids.append(row[1]['CHEM_ID'])
    chemid_to_chemname[row[1]['CHEM_ID']] = row[1]['CHEMICAL_NAME']
    pathway_mappings[row[1]['CHEM_ID']] = row[1]['SUPER_PATHWAY']
all_superpathways = list(set(pathway_mappings.values()))
mapped_pathways = [pathway_mappings[x] for x in df.columns[1:].tolist()]
chemnames = [chemid_to_chemname[x] for x in df.columns[1:].tolist()]
fig, ax = plt.subplots()
for superpath in all_superpathways:
    ax.scatter(
        pca.components_[0, np.array(mapped_pathways) == superpath],
        pca.components_[1, np.array(mapped_pathways) == superpath],
        s=3
    )
TOPN = 10
```

```
to_annotate = (-abs(pca.components_[0])).argsort()[:TOPN].tolist()
#  \
    # + (-pca.components_[0]).argsort()[:TOPN].tolist() \
    # + (pca.components_[1]).argsort()[:TOPN].tolist() \
    # + (-pca.components_[1]).argsort()[:TOPN].tolist()



chemname_to_text_offset = {
    'cytidine': (-10, 4),
    'pyruvate': (-12, 4),
    '1-stearoyl-GPC (18:0)': (-12, -6),
    '1-palmitoyl-GPC (16:0)': (-20, 4),
    'guanine': (2, -2),
    'mead acid (20:3n9)': (-15, 4),
    'X-25009': (-10, -6),
    '2-palmitoyl-GPC (16:0)*': (-20, 3),
    'dihomo-linolenate (20:3n3 or n6)': (11, 3),
    'linolenate [alpha or gamma; (18:3n3 or 6)]': (-16, 6),
}
annotations = []
for index in to_annotate:
    chemid = chemids[index]
    chemname = chemid_to_chemname[chemid]
    print(chemname)
    x = pca.components_[0][index]
    y = pca.components_[1][index]
    if 'mead acid' in chemname:
        name_to_display = 'mead acid'
    else:
        name_to_display = chemname.split(' ')[0]
    arrowprops = None
```

```
    # if name_to_display in ('mead acid'):
    #     arrowprops = dict(arrowstyle="->")
    annotations.append(ax.annotate(
        name_to_display, (x, y), fontsize=5,
        textcoords='offset points',
        # xytext=(-int(len(chemname)), 4)
        xytext=chemname_to_text_offset[chemname],
        arrowprops=arrowprops,
    ))
# adjust_text(annotations, only_move={'points':'y', 'texts':'y'}, arrowprops=dict(arrowstyle="->", color='r'))
plt.legend(all_superpathways, fontsize=6)
plt.xlim(-0.15, 0.2)
plt.ylim(-0.3, 0.2)
plt.xlabel(f'PC1 ({first_pc_explained_variance}%)', fontsize=12)
plt.ylabel(f'PC2 ({second_pc_explained_variance}%)', fontsize=12)
plt.axhline(y=0, color='black', linestyle=(0, (1, 4)), linewidth=1)
plt.axvline(x=0, color='black', linestyle=(0, (1, 4)), linewidth=1)
plt.savefig(f'pca_loading_{suffix}.png', dpi=600)
```

DONUT PIE-CHART FOR LIPIDS

```
import math
import pandas as pd
import numpy as np
from matplotlib import pyplot as plt
from sklearn.decomposition import PCA
from matplotlib.patches import Ellipse
import matplotlib.transforms as transforms
from adjustText import adjust_text
from collections import defaultdict

MAIN_DATA_FILE="/Users/Documents/human/NYBC-01-21VW+ CO DATA TABLES.XLSX"
```

```
# SHEET_NAME="Param_bradford_mgml-norm Data"
SHEET_NAME="Param_bradford_mgml-norm Data"
PATHWAY_SHEET_NAME="Chemical Annotation"

LIPID_MAPPING_DATA_FILE="//Users/Documents/human/NYBC-01-21VW+ CO DATA
TABLES.XLSX"
LIPID_MAPPING_SHEET_NAME="new lipid classification reform"

UNINFECTED_WILD_TYPE_SAMPLES=[
    "NYBC-00142",
    "NYBC-00143",
    "NYBC-00144",
]
INFECTED_WILD_TYPE_SAMPLES=[
    "NYBC-00145",
    "NYBC-00146",
    "NYBC-00147",
]
df = pd.read_excel(io=MAIN_DATA_FILE, sheet_name=SHEET_NAME)

df_uninfected = df[df['PARENT_SAMPLE_NAME'].isin(UNINFECTED_WILD_TYPE_SAMPLES)]
df_infected = df[df['PARENT_SAMPLE_NAME'].isin(INFECTED_WILD_TYPE_SAMPLES)]
column_indexer = df.columns != 'PARENT_SAMPLE_NAME'
lipid_chems = []
chemid_to_subpathway = {}
pathway_mapping_df = pd.read_excel(io=MAIN_DATA_FILE,
sheet_name=PATHWAY_SHEET_NAME)
for row in pathway_mapping_df.iterrows():
    if not isinstance(row[1]['SUPER_PATHWAY'], str):
        if math.isnan(row[1]['SUPER_PATHWAY']):
            row[1]['SUPER_PATHWAY'] = 'Uncharacterized Molecules'
```

```
    if row[1]['SUPER_PATHWAY'] != 'Lipid':
        continue
    lipid_chems.append(row[1]['CHEM_ID'])
    chemid_to_subpathway[row[1]['CHEM_ID']] = row[1]['SUB_PATHWAY']
lipid_mapping_df = pd.read_excel(io=LIPID_MAPPING_DATA_FILE,
sheet_name=LIPID_MAPPING_SHEET_NAME)
lipid_subpathway_to_grouping = {}
for row in lipid_mapping_df.iterrows():
    lipid_subpathway_to_grouping[row[1]['subpathway']] = row[1]['subpathway grouping']
subpathway_grouping_to_chemids = defaultdict(list)
for chemid in lipid_chems:
    subpathway = chemid_to_subpathway[chemid]
    if subpathway not in lipid_subpathway_to_grouping:
        print(subpathway)
        continue
    subpathway_grouping = lipid_subpathway_to_grouping[subpathway]
    subpathway_grouping_to_chemids[subpathway_grouping].append(chemid)
fig, axs = plt.subplots(1, 2)
index = 0
# colors =['#FFCE53', '#FFDA7E', '#FFE9B2', '#30B7EA', '#56C7F2', '#C8A2C9','#D6A7DF']
colors =['#ffb700', '#FFCE53', '#FFE9B2', '#ff9f80', '#ffc6b3', '#d9beda','#ecdfec']
for data, title in zip([df_uninfected, df_infected], ['Uninfected', 'Infected']):
    lipid_data = data[lipid_chems]
    lipid_data_mean = lipid_data.mean()
    subpathway_grouping_to_summed_val = {}
    subpathway_grouping_percentages = []
    subpathway_grouping_names = []
    for subpathway_grouping, subpathway_grouping_chemids in
subpathway_grouping_to_chemids.items():
        subpathway_grouping_to_summed_val[subpathway_grouping] =
lipid_data_mean[subpathway_grouping_chemids].sum()
```

```
subpathway_grouping_percentages.append(subpathway_grouping_to_summed_val[subpathway_grouping] * 100.0 / lipid_data_mean.sum())
        subpathway_grouping_names.append(subpathway_grouping)
    # wedges, texts =
    axs[index].pie(
        subpathway_grouping_percentages,
        wedgeprops=dict(width=0.3),
        startangle=90,
        autopct='%.2f%%',
        textprops={'fontsize': 3.5, 'weight': 'bold'},
        colors=colors,
        pctdistance=0.57
    )
    axs[index].set_title(title, y = 1.0, pad=-6)
    index += 1
plt.figlegend(subpathway_grouping_names, loc='lower center', fontsize=5, ncol=1, labelspacing=0., bbox_to_anchor=(0.51, 0.2))
plt.savefig(f'lipid_donut.png', dpi=600)
```

Supplementary File 3: MetaboAnalyst 5.0 report generated on the Pathway Impact Analysis and Metabolite set Enrichment Analysis (MSEA).

Metabolomic Data Analysis with MetaboAnalyst 5.0

Name: guest1426864616232074298

October 26, 2022

1 Background

MSEA or Metabolite Set Enrichment Analysis is a way to identify biologically meaningful patterns that are significantly enriched in quantitative metabolomic data. In conventional approaches, metabolites are evaluated individually for their significance under conditions of study. Those compounds that have passed certain significance level are then combined to see if any meaningful patterns can be discerned. In contrast, MSEA directly investigates if a set of functionally related metabolites without the need to preselect compounds based on some arbitrary cut-off threshold. It has the potential to identify subtle but consistent changes among a group of related compounds, which may go undetected with the conventional approaches.

Essentially, MSEA is a metabolomic version of the popular GSEA (Gene Set Enrichment Analysis) software with its own collection of metabolite set libraries as well as an implementation of user-friendly web-interfaces. GSEA is widely used in genomics data analysis and has proven to be a powerful alternative to conventional approaches. For more information, please refer to the original paper by Subramanian A, and a nice review paper by Nam D, Kim SY. [1]. [2]

2 MSEA Overview

Metabolite set enrichment analysis consists of four steps - data input, data processing, data analysis, and results download. Different analysis procedures are performed based on different input types. In addition, users can also browse and search the metabolite set libraries as well as upload their self-defined metabolite sets for enrichment analysis. Users can also perform metabolite name mapping between a variety of compound names, synonyms, and major database identifiers.

3 Data Input

There are three enrichment analysis algorithms offered by MSEA. Accordingly, three different types of data inputs are required by these three approaches:

- A list of important compound names - entered as a one column data (*Over Representation Analysis (ORA)*);
- A single measured biofluid (urine, blood, CSF) sample- entered as tab separated two-column data with the first column for compound name, and the second for concentration values (*Single Sample Profiling (SSP)*);

[1]Subramanian*Gene set enrichment analysis: A knowledge-based approach for interpreting genome-wide expression profiles.*, Proc Natl Acad Sci USA. 2005 102(43): 15545-50

[2]Nam D, Kim SY. *Gene-set approach for expression pattern analysis*, Briefings in Bioinformatics. 2008 9(3): 189-197.

- A compound concentration table - entered as a comma separated (.csv) file with the each sample per row and each metabolite concentration per column. The first column is sample names and the second column for sample phenotype labels (*Quantitative Enrichment Analysis (QEA)*)

You selected Over Representation Analysis (ORA) which requires a list of compound names as input.

4 Data Process

The first step is to standardize the compound labels. It is an essential step since the compound labels will be subsequently compared with compounds contained in the metabolite set library. MSEA has a built-in tool to convert between compound common names, synonyms, identifiers used in HMDB ID, PubChem, ChEBI, BiGG, METLIN, KEGG, or Reactome. **Table 1** shows the conversion results. Note: *1* indicates exact match, *2* indicates approximate match, and *0* indicates no match. A text file contain the result can be found the downloaded file *name_map.csv*

T

	Query	Match	HMDB	PubChem	KEGG	SMILES
1	HMDB0000123	Glycine	HMDB0000123	750	C00037	C(C(=O)O)N
2	HMDB0000532	Acetylglycine	HMDB0000532	10972		CC(=O)NCC(=O)O
3	HMDB0000271	Sarcosine	HMDB0000271	1088	C00213	CNCC(=O)O
4	HMDB0000092	Dimethylglycine	HMDB0000092	673	C01026	CN(C)CC(=O)O
5	HMDB0000043	Betaine	HMDB0000043	247	C00719	C[N+](C)(C)CC(=O)[
6	HMDB0000187	L-Serine	HMDB0000187	5951	C00065	C([C@@H](C(=O)O)N
7	HMDB0002931	N-Acetylserine	HMDB0002931	65249		CC(=O)N[C@@H](CO)
8	HMDB0000167	L-Threonine	HMDB0000167	6288	C00188	C[C@H]([C@@H](C(=
9	HMDB0062557	N-Acetylthreonine	HMDB0062557	152204		C[C@@H](O)[C@H](N
10	HMDB0011185	O-Phosphothreonine	HMDB0011185	3246323	C12147	C[C@H]([C@@H](C(=
11	HMDB0000161	L-Alanine	HMDB0000161	5950	C00041	C[C@@H](C(=O)O)N
12	HMDB0000766	N-Acetyl-L-alanine	HMDB0000766	88064		C[C@@H](C(=O)O)N
13	HMDB0000191	L-Aspartic acid	HMDB0000191	5960	C00049	C([C@@H](C(=O)O)N
14	HMDB0000812	N-Acetyl-L-aspartic acid	HMDB0000812	65065	C01042	CC(=O)N[C@@H](CC
15	HMDB0000168	L-Asparagine	HMDB0000168	6267	C00152	C([C@@H](C(=O)O)N
16	HMDB0006028	N-Acetylasparagine	HMDB0006028	99715		CC(=O)N[C@@H](CC
17	HMDB32332	Hydroxylated lecithin	HMDB0032332	97663	C03124	C([C@@H](C(=O)O)N
18	HMDB0000148	L-Glutamic acid	HMDB0000148	33032	C00025	C(CC(=O)O)[C@@H](
19	HMDB0000641	L-Glutamine	HMDB0000641	5961	C00064	C(CC(=O)N)[C@@H](
20	HMDB0001138	N-Acetylglutamic acid	HMDB0001138	185	C00624	CC(=O)NC(CCC(=O)
21	HMDB0006029	N-Acetylglutamine	HMDB0006029	25561		CC(=O)NC(CCC(=O)
22	HMDB0001344	NA	NA	NA	NA	NA
23	HMDB0061715	NA	NA	NA	NA	NA
24	HMDB0002201	N-Carboxyethyl-g-aminobutyric acid	HMDB0002201	2572		C(CC(=O)O)CNCCC(
25	HMDB0001301	1-Pyrroline-5-carboxylic acid	HMDB0001301	6642	C03912	C1CC(N=C1)C(=O)O
26	HMDB0000177	L-Histidine	HMDB0000177	6274	C00135	C1=C(NC=N1)C[C@@
27	HMDB0000001	1-Methylhistidine	HMDB0000001	92105	C01152	CN1C=C(N=C1)C[C@
28	HMDB0000479	3-Methylhistidine	HMDB0000479	64969	C01152	CN1C=NC=C1C[C@@
29	HMDB0032055	N-Acetylhistidine	HMDB0032055	273260	C02997	CC(=O)NC(CC1=CN=
30	HMDB0002320	Imidazolelactic acid	HMDB0002320	459122	C05132	C1=CN(C=N1)CC(C(
31	HMDB0000033	Carnosine	HMDB0000033	439224	C00386	C1=C(NC=N1)C[C@@
32	HMDB0000194	Anserine	HMDB0000194	112072	C01262	CN1C=NC=C1C[C@@
33	HMDB0000870	Histamine	HMDB0000870	774	C00388	C1=C(NC=N1)CCN
34	HMDB0002820	Methylimidazoleacetic acid	HMDB0002820	75810	C05828	CN1C=C(N=C1)CC(=
35	HMDB04988	Pi-Methylimidazoleacetic acid	HMDB0004988	6451814		CN1C=NC=C1CC(=O
36	HMDB0002331	Imidazoleacetic acid riboside	HMDB0002331	440569	C05131	C1=C(N=CN1[C@H]2[
37	HMDB0002024	Imidazoleacetic acid	HMDB0002024	96215	C02835	C1=C(NC=N1)CC(=O
38	HMDB0003405	D-Lysine	HMDB0003405	866	C00739	C(CCN)C[C@H](C(=O
39	HMDB0000206	N6-Acetyl-L-lysine	HMDB0000206	92832	C02727	CC(=O)NCCCC[C@@
40	HMDB0002038	N(6)-Methyllysine	HMDB0002038	164795	C02728	CNCCCC[C@@H](C(=
41	HMDB0013287	Ne,Ne dimethyllysine	HMDB0013287	4478779	C05545	CN(C)CCCCC(C(=O)
42	HMDB0001325	N6,N6,N6-Trimethyl-L-lysine	HMDB0001325	440120	C03793	C[N+](C)(C)CCCC[C
43	HMDB0034879	1-[(5-Amino-5-carboxypentyl)amino]-1-deoxyfructose	HMDB0034879	9839580	C16488	NC(CCCCNCC1(O)OC
44	HMDB0000510	Aminoadipic acid	HMDB0000510	469	C00956	C(CC(C(=O)O)N)CC(
45	HMDB0000070	Pipecolic acid	HMDB0000070	849	C00408	C1CCNC(C1)C(=O)O
46	HMDB0002284	N-Acetylcadaverine	HMDB0002284	189087		CC(=O)NCCCCCN
47	HMDB0003355	5-Aminopentanoic acid	HMDB0003355	138	C00431	C(CCN)CC(=O)O
48	HMDB0000159	L-Phenylalanine	HMDB0000159	6140	C00079	C1=CC=C(C=C1)C[C
49	HMDB0000512	N-Acetyl-L-phenylalanine	HMDB0000512	74839	C03519	CC(=O)N[C@@H](CC
50	HMDB0000158	L-Tyrosine	HMDB0000158	6057	C00082	C1=CC(=CC=C1C[C
51	HMDB0000755	Hydroxyphenyllactic acid	HMDB0000755	9378	C03672	C1=CC(=CC=C1CC(
52	HMDB0060015	Phenyl hydrogen sulfate	HMDB0060015	74426	C02180	OS(=O)(=O)OC1=CC
53	HMDB0240317	NA	NA	NA	NA	NA
54	HMDB0000929	L-Tryptophan	HMDB0000929	6305	C00078	C1=CC=C2C(=C1)C(
55	HMDB0240296	NA	NA	NA	NA	NA
56	HMDB0061115	Lenticin	HMDB0061115	442106	C09213	C[N+](C)(C)[C@@H](
57	HMDB01200	N'-Formylkynurenine	HMDB0001200	910	C02406	C1=CC=C(C(=C1)C(
58	HMDB0000684	L-Kynurenine	HMDB0000684	161166	C00328	C1=CC=C(C(=C1)C(
59	HMDB0000671	Indolelactic acid	HMDB0000671	92904	C02043	C1=CC=C2C(=C1)C(

60	HMDB0000682	Indoxyl sulfate	HMDB0000682	10258		C1=CC=C2C(=C1)C(
61	HMDB0000687	L-Leucine	HMDB0000687	6106	C00123	CC(C)C[C@@H](C(=O
62	HMDB0000695	Ketoleucine	HMDB0000695	70	C00233	CC(C)CC(=O)C(=O)O
63	HMDB0000688	Isovalerylcarnitine	HMDB0000688	6426851		CC(C)CC(=O)OC(CC
64	HMDB0000754	3-Hydroxyisovaleric acid	HMDB0000754	69362	C20827	CC(C)(CC(=O)O)O
65	HMDB0000172	L-Isoleucine	HMDB0000172	6306	C00407	CC[C@H](C)[C@@H](C
66	HMDB0061684	N-Acetylisoleucine	HMDB0061684	7036275		CC[C@H](C)[C@H](NC
67	HMDB0000491	3-Methyl-2-oxovaleric acid	HMDB0000491	47	C00671	CCC(C)C(=O)C(=O)O
68	HMDB0000378	2-Methylbutyroylcarnitine	HMDB0000378	6426901		CCC(C)C(=O)OC(CC
69	HMDB0002366	Tiglylcarnitine	HMDB0002366	91825636		C/C=C(\C)/C(=O)O[
70	HMDB0001844	Methylsuccinic acid	HMDB0001844	10349	C08645	CC(CC(=O)O)C(=O)O
71	HMDB0000883	L-Valine	HMDB0000883	6287	C00183	CC(C)[C@@H](C(=O)
72	HMDB0011757	N-Acetylvaline	HMDB0011757	227752		CC(C)C(C(=O)O)NC(
73	HMDB0000019	Alpha-ketoisovaleric acid	HMDB0000019	49	C00141	CC(C)C(=O)C(=O)O
74	HMDB0000407	2-Hydroxy-3-methylbutyric acid	HMDB0000407	99823		CC(C)C(C(=O)O)O
75	HMDB0000736	Isobutyryl-L-carnitine	HMDB0000736	168379		CC(C)C(=O)OC(CC(
76	HMDB0000696	L-Methionine	HMDB0000696	6137	C00073	CSCC[C@@H](C(=O)O
77	HMDB0011745	N-Acetyl-L-methionine	HMDB0011745	6180	C02712	CC(=O)NC(CCSC)C(
78	HMDB0001015	N-Formyl-L-methionine	HMDB0001015	6995182	C03145	CSCC[C@H](C(=O)O)
79	HMDB0062174	NA	NA	NA	NA	NA
80	HMDB0002005	Methionine sulfoxide	HMDB0002005	847	C02989	CS(=O)CCC(C(=O)O)
81	HMDB0000939	S-Adenosylhomocysteine	HMDB0000939	439155	C00021	C1=NC2=C(C(=N1)N
82	HMDB0001087	5-Methylthioribose	HMDB0001087	439904	C03089	CSC[C@@H]1[C@H]([C
83	HMDB0240388	NA	NA	NA	NA	NA
84	HMDB0000742	Homocysteine	HMDB0000742	778	C00155	C(CS)C(C(=O)O)N
85	HMDB0000574	L-Cysteine	HMDB0000574	5862	C00097	C([C@@H](C(=O)O)N
86	HMDB0001890	Acetylcysteine	HMDB0001890	12035	C06809	CC(=O)N[C@@H](CS)
87	HMDB0029432	(S)C(S)S-S-Methylcysteine sulfoxide	HMDB0029432	82142		CS(=O)CC(C(=O)O)N
88	HMDB0000996	3-Sulfinoalanine	HMDB0000996	439270	C00606	C([C@@H](C(=O)O)N
89	HMDB0000965	Hypotaurine	HMDB0000965	107812	C00519	C(CS(=O)O)N
90	HMDB0000251	Taurine	HMDB0000251	1123	C00245	C(CS(=O)(=O)O)N
91	HMDB0240253	NA	NA	NA	NA	NA
92	HMDB0002757	Cysteic acid	HMDB0002757	25701	C00506	C(C(C(=O)O)N)S(=O
93	HMDB0000517	L-Arginine	HMDB0000517	6322	C00062	C(C[C@@H](C(=O)O)
94	HMDB0000294	Urea	HMDB0000294	2447	C00086	C(=O)(N)N
95	HMDB0000214	Ornithine	HMDB0000214	6262	C00077	C(C[C@@H](C(=O)O)
96	HMDB0000323	3-Amino-2-piperidone	HMDB0000323	5200225		C1CC(C(=O)NC1)N
97	HMDB0004225	2-Oxoarginine	HMDB0004225	558	C03771	C(CC(=O)C(=O)O)CN
98	HMDB0000904	Citrulline	HMDB0000904	9750	C00327	C(C[C@@H](C(=O)O)
99	HMDB0000162	NA	NA	NA	NA	NA
100	HMDB0003411	D-Proline	HMDB0003411	8988	C00763	C1C[C@@H](NC1)C(
101	HMDB0003334	NA	NA	NA	NA	NA
102	HMDB0001539	Asymmetric dimethylarginine	HMDB0001539	123831	C03626	CN(C)C(=NCCC[C@
103	HMDB0004620	N-a-Acetyl-L-arginine	HMDB0004620	67427		CC(=O)N[C@@H](CC
104	HMDB0000725	4-Hydroxyproline	HMDB0000725	5810	C01157	C1[C@H](CN[C@@H]1
105	HMDB0094696	N-Methyl-proline	HMDB0094696	643474		CN1CCC[C@H]1C(O)=
106	HMDB0240365	NA	NA	NA	NA	NA
107	HMDB0000128	Guanidoacetic acid	HMDB0000128	763	C00581	C(C(=O)O)N=C(N)N
108	HMDB0000064	Creatine	HMDB0000064	586	C00300	CN(CC(=O)O)C(=N)N
109	HMDB0000562	Creatinine	HMDB0000562	588	C00791	CN1CC(=O)N=C1N
110	HMDB0001511	Phosphocreatine	HMDB0001511	587	C02305	CN(CC(=O)O)C(=NP
111	HMDB0002064	N-Acetylputrescine	HMDB0002064	122356	C02714	CC(=O)NCCCCN
112	HMDB0001257	Spermidine	HMDB0001257	1102	C00315	C(CCNCCCN)CN
113	HMDB0001276	NA	NA	NA	NA	NA
114	HMDB0002189	N8-Acetylspermidine	HMDB0002189	123689	C01029	CC(=O)NCCCCNCCC
115	HMDB0001256	Spermine	HMDB0001256	1103	C00750	C(CCNCCCN)CNCCC
116	HMDB0001186	N1-Acetylspermine	HMDB0001186	916	C02567	CC(=O)NCCCNCCCC
117	HMDB0001173	5'-Methylthioadenosine	HMDB0001173	439176	C00170	CSC[C@@H]1[C@H]([C
118	HMDB0001522	Methylguanidine	HMDB0001522	10111	C02294	CN=C(N)N
119	HMDB0003464	4-Guanidinobutanoic acid	HMDB0003464	500	C01035	C(CC(=O)O)CN=C(N
120	HMDB0000125	NA	NA	NA	NA	NA
121	HMDB0003337	Oxidized glutathione	HMDB0003337	975	C00127	C(CC(=O)NC(CSSCC
122	HMDB0000656	Cysteineglutathione disulfide	HMDB0000656	53477713		C(CC(=O)N[C@@H](C
123	HMDB0001066	S-Lactoylglutathione	HMDB0001066	440018	C03451	C[C@H](C(=O)SC[C@
124	HMDB0000078	Cysteinylglycine	HMDB0000078	439498	C01419	C([C@@H](C(=O)NCC
125	HMDB0000267	Pyroglutamic acid	HMDB0000267	7405	C01879	C1CC(=O)N[C@@H]1
126	HMDB0000729	NA	NA	NA	NA	NA
127	HMDB00008	2-Hydroxybutyric acid	HMDB0000008	11266	C05984	CCC(C(=O)O)O
128	HMDB0005765	Ophthalmic acid	HMDB0005765	7018721	C21016	CC[C@@H](C(=O)NCC
129	HMDB0240578	NA	NA	NA	NA	NA
130	HMDB0001049	gamma-Glutamylcysteine	HMDB0001049	123938	C00669	C(CC(=O)N[C@@H](C
131	HMDB0011737	gamma-Glutamylglutamic acid	HMDB0011737	92865	C05282	C(CC(=O)N[C@@H](C
132	HMDB0011738	N2-gamma-Glutamylglutamine	HMDB0011738	150914	C05283	C(CC(=O)N[C@@H](C
133	HMDB0011170	gamma-Glutamylisoleucine	HMDB0011170	22885096		CC[C@H](C)[C@@H](C
134	HMDB0011171	gamma-Glutamylleucine	HMDB0011171	4524287		CC(C)C[C@@H](C(=O
135	HMDB0029159	gamma-Glutamylthreonine	HMDB0029159	53861142		C[C@H]([C@@H](C(=
136	HMDB0011172	gamma-Glutamylvaline	HMDB0011172	7015683		CC(C)[C@@H](C(=O)
137	HMDB0028818	Glutamylglutamic acid	HMDB0028818	439500	C01425	C(CC(=O)O)[C@@H](
138	HMDB0028749	Aspartyl-Aspartate	HMDB0028749	471583		NC(CC(O)=O)C(=O)N
139	HMDB0000759	Glycylleucine	HMDB0000759	92843	C02155	CC(C)C[C@@H](C(=O
140	HMDB0000721	Glycylproline	HMDB0000721	79101		C1CC(N(C1)C(=O)CN
141	HMDB0028854	Glycyl-Valine	HMDB0028854	97417		CC(C)C(NC(=O)CN)C
142	HMDB0028988	Phenylalanyl-Alanine	HMDB0028988	5488196		CC(NC(=O)C(N)CC1=
143	HMDB0029010	Prolyl-Alanine	HMDB0029010	6347578		CC(NC(=O)C1CCCN1
144	HMDB0011178	Prolylglycine	HMDB0011178	98206		C1CC(NC1)C(=O)NCC
145	HMDB0011180	L-prolyl-L-proline	HMDB0011180	263469		C1CC(NC1)C(=O)N2C
146	HMDB0029127	Valyl-Glycine	HMDB0029127	6993111		CC(C)C(N)C(=O)NCC

147	HMDB0029131	Valyl-Leucine	HMDB0029131	6993118		CC(C)CC(NC(=O)C(N
148	HMDB0006344	Alpha-N-Phenylacetyl-L-glutamine	HMDB0006344	92258	C04148	C1=CC=C(C=C1)CC(
149	HMDB0000821	Phenylacetylglycine	HMDB0000821	68144	C05598	C1=CC=C(C=C1)CC(
150	HMDB0000122	D-Glucose	HMDB0000122	5793	C00221	C([C@@H]1[C@H]([C@
151	HMDB0001401	Glucose 6-phosphate	HMDB0001401	5958	C00092	C([C@@H]1[C@H]([C@
152	HMDB0003514	NA	NA	NA	NA	NA
153	HMDB0001058	Fructose 1,6-bisphosphate	HMDB0001058	445557	C05378	C([C@@H]1[C@H]([C@
154	HMDB0001294	2,3-Diphosphoglyceric acid	HMDB0001294	186004	C01159	C([C@H](C(=O)O)OP(
155	HMDB0001473	Dihydroxyacetone phosphate	HMDB0001473	668	C00111	C(C(=O)COP(=O)(O)
156	HMDB0000362	2-Phosphoglyceric acid	HMDB0000362	59		C(C(C(=O)O)OP(=O)
157	HMDB0000807	3-Phosphoglyceric acid	HMDB0000807	724	C00597	C(C(C(=O)O)O)OP(=
158	HMDB0000263	Phosphoenolpyruvic acid	HMDB0000263	1005	C00074	C=C(C(=O)O)OP(=O
159	HMDB0000243	Pyruvic acid	HMDB0000243	1060	C00022	CC(=O)C(=O)O
160	HMDB0000190	L-Lactic acid	HMDB0000190	61503	C00186	C[C@@H](C(=O)O)O
161	HMDB0006372	NA	NA	NA	NA	NA
162	HMDB0000139	Glyceric acid	HMDB0000139	439194	C00258	C([C@H](C(=O)O)O)O
163	HMDB0001316	6-Phosphogluconic acid	HMDB0001316	91493	C00345	C([C@H]([C@H]([C@@
164	HMDB0000280	Phosphoribosyl pyrophosphate	HMDB0000280	7339	C00119	C([C@@H]1[C@H]([C@
165	HMDB0001068	D-Sedoheptulose 7-phosphate	HMDB0001068	22833559	C05382	C([C@@H]1[C@H]([C@
166	HMDB0002917	NA	NA	NA	NA	NA
167	HMDB0000508	NA	NA	NA	NA	NA
168	HMDB0001851	NA	NA	NA	NA	NA
169	HMDB0000568	D-Arabitol	HMDB0000568	827	C01904	C(C(C(C(CO)O)O)O)O
170	HMDB0000867	Ribonic acid	HMDB0000867	5460677	C01685	C([C@H]([C@H]([C@H]
171	HMDB0000621	NA	NA	NA	NA	NA
172	HMDB0000751	NA	NA	NA	NA	NA
173	HMDB0001644	NA	NA	NA	NA	NA
174	HMDB0003371	L-Ribulose	HMDB0003371	439204	C00508	C1[C@H]([C@@H](C(O
175	HMDB0000539	Arabinonic acid	HMDB0000539	122045	C00878	C([C@H]([C@H]([C@@
176	HMDB0060255	L-Lyxonate	HMDB0060255	644110	C05412	OC[C@H](O)[C@@H](
177	HMDB0000258	Sucrose	HMDB0000258	5988	C00089	C([C@@H]1[C@H]([C@
178	HMDB0000660	D-Fructose	HMDB0000660	439709	C02336	C([C@@H]1[C@H]([C@
179	HMDB0000247	NA	NA	NA	NA	NA
180	HMDB0000765	Mannitol	HMDB0000765	6251	C00392	C([C@H]([C@H]([C@@
181	HMDB0000169	D-Mannose	HMDB0000169	18950	C00936	C([C@@H]1[C@H]([C@
182	HMDB0000565	Galactonic acid	HMDB0000565	128869	C00880	C([C@H]([C@@H]([C@
183	HMDB0000286	Uridine diphosphate glucose	HMDB0000286	53477679	C00029	C1=CN(C(=O)NC1=C
184	HMDB0000302	Uridine diphosphategalactose	HMDB0000302	18068	C00052	C1=CN(C(=O)NC1=C
185	HMDB0000935	Uridine diphosphate glucuronic acid	HMDB0000935	17473	C00167	C1=CN(C(=O)NC1=C
186	HMDB0000304	NA	NA	NA	NA	NA
187	HMDB0000290	Uridine diphosphate-N-acetylglucosamine	HMDB0000290	9547196	C00043	CC(=O)N[C@@H]1[C@
188	HMDB0000230	N-Acetylneuraminic acid	HMDB0000230	445063	C19910	CC(=O)N[C@@H]1[C@
189	HMDB0000489	Aspartylglycosamine	HMDB0000489	123826	C04540	CC(=O)N[C@@H]1[C@
190	HMDB0000613	Erythronic acid	HMDB0000613	2781043		C([C@H]([C@H](C(=O
191	HMDB0000212	NA	NA	NA	NA	NA
192	HMDB0000215	N-Acetyl-D-glucosamine	HMDB0000215	439174	C00140	CC(=O)N[C@@H]1[C@
193	HMDB0240347	NA	NA	NA	NA	NA
194	HMDB0000094	Citric acid	HMDB0000094	311	C00158	C(C(=O)O)C(CC(=O)
195	HMDB0000958	NA	NA	NA	NA	NA
196	HMDB000072	NA	NA	NA	NA	NA
197	HMDB0001874	D-threo-Isocitric acid	HMDB0001874	5318532	C00451	C([C@@H]([C@H](C(=
198	HMDB0000208	Oxoglutaric acid	HMDB0000208	51	C00026	C(CC(=O)O)C(=O)C(
199	HMDB0061717	NA	NA	NA	NA	NA
200	HMDB0000254	Succinic acid	HMDB0000254	1110	C00042	C(CC(=O)O)C(=O)O
201	HMDB0000134	Fumaric acid	HMDB0000134	444972	C00122	C(=C/C(=O)O)\C(=C
202	HMDB0000744	NA	NA	NA	NA	NA
203	HMDB0031518	NA	NA	NA	NA	NA
204	HMDB0000156	L-Malic acid	HMDB0000156	222656	C00149	C([C@@H](C(=O)O)O)
205	HMDB0001494	Acetylphosphate	HMDB0001494	186	C00227	CC(=O)OP(=O)(O)O
206	HMDB0001429	Phosphate	HMDB0001429	57424078	C00009	[O-]P(=O)([O-])[O-]
207	HMDB0002095	Malonylcarnitine	HMDB0002095	22833583		C[N+](C)(C)C[C@H](C
208	HMDB0000691	Malonic acid	HMDB0000691	867	C04025	C(C(=O)O)C(=O)O
209	HMDB0000535	Caproic acid	HMDB0000535	8892	C01585	CCCCCC(=O)O
210	HMDB0000826	Pentadecanoic acid	HMDB0000826	13849	C16537	CCCCCCCCCCCCCC
211	HMDB0000220	Palmitic acid	HMDB0000220	985	C00249	CCCCCCCCCCCCCC
212	HMDB0002259	Heptadecanoic acid	HMDB0002259	10465		CCCCCCCCCCCCCC
213	HMDB0000827	Stearic acid	HMDB0000827	5281	C01530	CCCCCCCCCCCCCC
214	HMDB0000772	Nonadecanoic acid	HMDB0000772	12591	C16535	CCCCCCCCCCCCCC
215	HMDB0002212	Arachidic acid	HMDB0002212	10467	C06425	CCCCCCCCCCCCCC
216	HMDB0002000	Myristoleic acid	HMDB0002000	5281119	C08322	CCCC/C=C\CCCCCC
217	HMDB0003229	Palmitoleic acid	HMDB0003229	5312427	C08362	CCCCCC/C=C\CCCC
218	HMDB0060038	10Z-Heptadecenoic acid	HMDB0060038	5312435		CCCCCC\C=C/CCCC
219	HMDB0000207	NA	NA	NA	NA	NA
220	HMDB0000573	NA	NA	NA	NA	NA
221	HMDB0003231	NA	NA	NA	NA	NA
222	HMDB0240219	cis-Vaccenic acid	HMDB0240219	5282761	C08367	CCCCCC\C=C/CCCC
223	HMDB0013622	Nonadeca-10(Z)-enoic acid	HMDB0013622	5312513	C00174	CCCCCCCC/C=C\CC
224	HMDB0002231	NA	NA	NA	NA	NA
225	HMDB0062436	9Z-Eicosenoic acid	HMDB0062436	5282767		[H]\C(CCCCCCCCCC
226	HMDB0002068	Erucic acid	HMDB0002068	5281116	C08316	CCCCCCCC/C=C\CC
227	HMDB0000560	5,8-Tetradecadienoic acid	HMDB0000560	5312409		CCCCC/C=C\C/C=C
228	HMDB0006547	Stearidonic acid	HMDB0006547	5312508	C16300	CC/C=C\C/C=C\C/C
229	HMDB0001999	Eicosapentaenoic acid	HMDB0001999	446284	C06428	CC/C=C\C/C=C\C/C
230	HMDB0001976	NA	NA	NA	NA	NA
231	HMDB0006528	Docosapentaenoic acid	HMDB0006528	5497182	C16513	CC/C=C\C/C=C\C/C
232	HMDB0002183	Docosahexaenoic acid	HMDB0002183	445580	C06429	CC/C=C\C/C=C\C/C
233	HMDB0002823	Docosatrienoic acid	HMDB0002823	5312557		CC/C=C\C/C=C\C/C

234	HMDB0000477	7Z,10Z-Hexadecadienoic acid	HMDB0000477	13932172		CCCCC/C=C\C/C=C
235	HMDB0000673	NA	NA	NA	NA	NA
236	HMDB0006270	Linoelaidic acid	HMDB0006270	5282457		CCCCC/C=C/C/C=C
237	HMDB0001388	NA	NA	NA	NA	NA
238	HMDB0003073	Gamma-Linolenic acid	HMDB0003073	5280933	C06426	CCCCC/C=C\C/C=C
239	HMDB0005060	Eicosadienoic acid	HMDB0005060	6439848	C16525	CCCCC/C=C\C/C=C
240	HMDB0002925	8,11,14-Eicosatrienoic acid	HMDB0002925	5280581	C03242	CCCCC/C=C\C/C=C
241	HMDB0001043	Arachidonic acid	HMDB0001043	444899	C00219	CCCCC/C=C\C/C=C
242	HMDB0002226	Adrenic acid	HMDB0002226	5497181	C16527	CCCCC/C=C\C/C=C
243	HMDB0001976	Docosapentaenoic acid (22n-6)	HMDB0001976	6441454		CCCCC/C=C\C/C=C
244	HMDB0061714	Docosadienoate (22:2n6)	HMDB0061714	5282807	C16533	CCCCCC=CCC=CCC
245	HMDB0010378	5,8,11-Eicosatrienoic acid	HMDB0010378	5312531		CCCCCCCC/C=C\C/
246	HMDB0061859	Methyl hexadecanoic acid	HMDB0061859	8181	C16995	CCCCCCCCCCCCCC
247	HMDB0037397	xi-17-Methyloctadecanoic acid	HMDB0037397	3083779		CC(C)CCCCCCCCCC
248	HMDB0000661	Glutaric acid	HMDB0000661	743	C00489	C(CC(=O)O)CC(=O)O
249	HMDB0059655	2-Hydroxyglutarate	HMDB0059655	43	C02630	OC(CCC(O)=O)C(O)=
250	HMDB0000176	Maleic acid	HMDB0000176	444972	C01384	C(=C\C(=O)O)\C(=O
251	HMDB0000623	Dodecanedioic acid	HMDB0000623	12736	C02678	C(CCCCCC(=O)O)CC
252	HMDB0000824	Propionylcarnitine	HMDB0000824	107738	C03017	CCC(=O)OC(CC(=O)
253	HMDB0013034	Palmitoylglycine	HMDB0013034	151008		CCCCCCCCCCCCCC
254	HMDB0000201	L-Acetylcarnitine	HMDB0000201	7045767	C02571	CC(=O)OC(CC(=O)[C
255	HMDB0000756	L-Hexanoylcarnitine	HMDB0000756	3246938		CCCCCC(=O)O[C@H]
256	HMDB0000791	L-Octanoylcarnitine	HMDB0000791	11953814	C02838	CCCCCCCC(=O)O[C@
257	HMDB0000651	Decanoylcarnitine	HMDB0000651	10245190		CCCCCCCCCC(=O)O
258	HMDB000225	NA	NA	NA	NA	NA
259	HMDB0005066	Tetradecanoylcarnitine	HMDB0005066	53477791		CCCCCCCCCCCCC(
260	HMDB0062517	NA	NA	NA	NA	NA
261	HMDB0000222	L-Palmitoylcarnitine	HMDB0000222	11953816	C02990	CCCCCCCCCCCCCC
262	HMDB0006210	Heptadecanoyl carnitine	HMDB0006210	53477803		CCCCCCCCCCCCCC
263	HMDB0000848	Stearoylcarnitine	HMDB0000848	52922056		CCCCCCCCCCCCCC
264	HMDB0006460	Arachidyl carnitine	HMDB0006460	53477833		CCCCCCCCCCCCCC
265	HMDB0062468	NA	NA	NA	NA	NA
266	HMDB0240665	NA	NA	NA	NA	NA
267	HMDB0006347	Hexacosanoyl carnitine	HMDB0006347	53477828		CCCCCCCCCCCCCC
268	HMDB0013205	9-Decenoylcarnitine	HMDB0013205	53481651		C[N+](C)(C)CC(CC(=
269	HMDB13326	trans-2-Dodecenoylcarnitine	HMDB0013326	53481671		CCCCCCCCC/C=C/C
270	HMDB0240588	NA	NA	NA	NA	NA
271	HMDB0013207	9-Hexadecenoylcarnitine	HMDB0013207	53481653		CCCCCC/C=C\CCCC
272	HMDB0005065	Oleoylcarnitine	HMDB0005065	46907933		CCCCCCCC/C=C\CC
273	HMDB0006509	Nervonyl carnitine	HMDB0006509	29385		CCC[N+](C)(C)C
274	HMDB0006469	Linoleyl carnitine	HMDB0006469	6450015		CCCCC/C=C\C/C=C
275	HMDB0006455	Arachidonyl carnitine	HMDB0006455	53477832		CCCCCCCCC/C=C\C
276	HMDB0013127	Hydroxybutyrylcarnitine	HMDB0013127	53481617		CC(CC(=O)O[C@@H](
277	HMDB0061636	NA	NA	NA	NA	NA
278	HMDB0013336	3-Hydroxyhexadecanoylcarnitine	HMDB0013336	53481691		CCCCCCCCCCCCC(
279	HMDB0001161	4-Trimethylammoniobutanoic acid	HMDB0001161	134	C01181	C[N+](C)(C)CCCC(=C
280	HMDB0000062	L-Carnitine	HMDB0000062	2724480	C00318	C[N+](C)(C)C[C@H](C
281	HMDB0000011	NA	NA	NA	NA	NA
282	HMDB0000357	NA	NA	NA	NA	NA
283	HMDB0000442	(S)-3-Hydroxybutyric acid	HMDB0000442	94318	C03197	C[C@@H](CC(=O)O)C
284	HMDB0240592	NA	NA	NA	NA	NA
285	HMDB0240596	NA	NA	NA	NA	NA
286	HMDB0062549	2-Hydroxystearic acid	HMDB0062549	439887	C03045	CCCCCCCCCCCCCC
287	HMDB0004667	NA	NA	NA	NA	NA
288	HMDB0004670	Alpha-dimorphecolic acid	HMDB0004670	5312830	C14767	CCCCC/C=C\C=C\[C
289	HMDB0002453	4-Deoxythreonic acid	HMDB0002453	10964471		C[C@H]([C@@H](C(=C
290	HMDB0000498	4-Deoxyerythronic acid	HMDB0000498	13120901		C[C@H]([C@H](C(=O)
291	HMDB0000360	2,4-Dihydroxybutanoic acid	HMDB0000360	192742		C(CO)C(C(=O)O)O
292	HMDB0002088	N-Oleoylethanolamine	HMDB0002088	5283454	C20792	CCCCCCCC/C=C\CC
293	HMDB0002100	Palmitoylethanolamide	HMDB0002100	4671	C16512	CCCCCCCCCCCCCC
294	HMDB0004080	Anandamide	HMDB0004080	5281969	C11695	CCCCC/C=C\C/C=C
295	HMDB0012252	Linoleoyl ethanolamide	HMDB0012252	5283446		CCCCC/C=C\C/C=C
296	HMDB0000211	myo-Inositol	HMDB0000211		C00137	O[C@H]1[C@H](O)[C@
297	HMDB0000097	Choline	HMDB0000097	305	C00114	C[N+](C)(C)CCO
298	HMDB0001565	Phosphorylcholine	HMDB0001565	8691	C00588	C[N+](C)(C)CCOP(=C
299	HMDB0001413	Citicoline	HMDB0001413	13804	C00307	C[N+](C)(C)CCOP(=C
300	HMDB0000086	Glycerophosphocholine	HMDB0000086	71920	C00670	C[N+](C)(C)CCOP(=C
301	HMDB0000224	O-Phosphoethanolamine	HMDB0000224	1015	C00346	C(COP(=O)(O)O)N
302	HMDB0001564	CDP-Ethanolamine	HMDB0001564	123727	C00570	C1=CN(C(=O)N=C1N
303	HMDB0000114	Glycerylphosphorylethanolamine	HMDB0000114	22833510	C01233	C(CO)N(CC(CO)O)P(
304	HMDB0000925	Trimethylamine N-oxide	HMDB0000925	1145	C01104	C[N+](C)(C)[O-]
305	HMDB0007869	PC(14:0/16:0)	HMDB0007869	129657	C00157	CCCCCCCCCCCCCC
306	HMDB0007883	PC(14:0/20:4(5Z,8Z,11Z,14Z))	HMDB0007883	24778634	C00157	CCCCCCCCCCCCC(
307	HMDB0007940	PC(15:0/18:2(9Z,12Z))	HMDB0007940	24778664	C00157	CCCCCCCCCCCCCC
308	HMDB0007949	PC(15:0/20:4(5Z,8Z,11Z,14Z))	HMDB0007949	52922330	C00157	CCCCCCCCCCCCCC
309	HMDB0007958	PC(15:0/22:6(4Z,7Z,10Z,13Z,16Z,19Z))	HMDB0007958	52922342	C00157	CCCCCCCCCCCCCC
310	HMDB0007967	PC(16:0/15:0)	HMDB0007967	24778680	C00157	CCCCCCCCCCCCCC
311	HMDB0000564	PC(16:0/16:0)	HMDB0000564	452110	C00157	CCCCCCCCCCCCCC
312	HMDB0007969	PC(16:0/16:1(9Z))	HMDB0007969	6443788	C00157	CCCCCCCCCCCCCC
313	HMDB0007970	PC(16:0/18:0)	HMDB0007970	24778686	C00157	CCCCCCCCCCCCCC
314	HMDB0007972	PC(16:0/18:1(9Z))	HMDB0007972	5497103	C00157	CCCCCCCCCCCCCC
315	HMDB0007973	PC(16:0/18:2(9Z,12Z))	HMDB0007973	5287971	C00157	CCCCCCCCCCCCCC
316	HMDB0007982	PC(16:0/20:4(5Z,8Z,11Z,14Z))	HMDB0007982	10747814	C00157	CCCCCCCCCCCCCC
317	HMDB0007984	PC(16:0/20:5(5Z,8Z,11Z,14Z,17Z))	HMDB0007984	24778723	C00157	CCCCCCCCCCCCCC
318	HMDB0007991	PC(16:0/22:6(4Z,7Z,10Z,13Z,16Z,19Z))	HMDB0007991	6441886	C00157	CCCCCCCCCCCCCC
319	HMDB0008006	PC(16:1(9Z)/18:2(9Z,12Z))	HMDB0008006	24778768	C00157	CCCCCC/C=C\CCCC
320	HMDB0008008	PC(16:1(9Z)/18:3(9Z,12Z,15Z))	HMDB0008008	52922452	C00157	CCCCCC/C=C\CCCC

321	HMDB0008036	PC(18:0/18:0)	HMDB0008036	94190	C00157	CCCCCCCCCCCCCCC
322	HMDB0008038	PC(18:0/18:1(9Z))	HMDB0008038	24778825	C00157	CCCCCCCCCCCCCCC
323	HMDB0008045	NA	NA	NA	NA	NA
324	HMDB0008143	PC(18:2(9Z,12Z)/20:0)	HMDB0008143	52922739	C00157	CCCCCCCCCCCCCCC
325	HMDB0008047	PC(18:0/20:3(8Z,11Z,14Z))	HMDB0008047	24778857	C00157	CCCCCCCCCCCCCCC
326	HMDB0008046	PC(18:0/20:3(5Z,8Z,11Z))	HMDB0008046	24778855	C00157	CCCCCCCCCCCCCCC
327	HMDB0008048	PC(18:0/20:4(5Z,8Z,11Z,14Z))	HMDB0008048	16219824	C00157	CCCCCCCCCCCCCCC
328	HMDB0008054	PC(18:0/22:4(7Z,10Z,13Z,16Z))	HMDB0008054	24778868	C00157	CCCCCCCCCCCCCCC
329	HMDB0008056	PC(18:0/22:5(7Z,10Z,13Z,16Z,19Z))	HMDB0008056	24778873	C00157	CCCCCCCCCCCCCCC
330	HMDB0008055	PC(18:0/22:5(4Z,7Z,10Z,13Z,16Z))	HMDB0008055	24778871	C00157	CCCCCCCCCCCCCCC
331	HMDB0008057	PC(18:0/22:6(4Z,7Z,10Z,13Z,16Z,19Z))	HMDB0008057	24778876	C00157	CCCCCCCCCCCCCCC
332	HMDB0008105	PC(18:1(9Z)/18:2(9Z,12Z))	HMDB0008105	24778939	C00157	CCCCCCCC/C=C\CC
333	HMDB0008123	PC(18:1(9Z)/22:6(4Z,7Z,10Z,13Z,16Z,19Z))	HMDB0008123	24778955	C00157	CCCCCCCC/C=C\CC
334	HMDB0008138	PC(18:2(9Z,12Z)/18:2(9Z,12Z))	HMDB0008138	5288075	C00157	CCCCC/C=C\C/C=C
335	HMDB0008141	PC(18:2(9Z,12Z)/18:3(9Z,12Z,15Z))	HMDB0008141	52922731	C00157	CCCCC/C=C\C/C=C
336	HMDB0008147	PC(18:2(9Z,12Z)/20:4(5Z,8Z,11Z,14Z))	HMDB0008147	24778979	C00157	CCCCC/C=C\C/C=C
337	HMDB0008279	PC(20:0/20:4(5Z,8Z,11Z,14Z))	HMDB0008279	24779048	C00157	CCCCCCCCCCCCCCC
338	HMDB0008923	PE(16:0/16:0)	HMDB0008923	445468	C00350	CCCCCCCCCCCCCCC
339	HMDB0008924	PE(16:0/16:1(9Z))	HMDB0008924	52924925	C00350	CCCCCCCCCCCCCCC
340	HMDB08925	PE(16:0/18:0)	HMDB0008925	5326793	C00350	CCCCCCCCCCCCCCC
341	HMDB0005320	NA	NA	NA	NA	NA
342	HMDB0005322	NA	NA	NA	NA	NA
343	HMDB0005323	NA	NA	NA	NA	NA
344	HMDB0008939	PE(16:0/20:5(5Z,8Z,11Z,14Z,17Z))	HMDB0008939	52924919	C00350	CCCCCCCCCCCCCCC
345	HMDB0008946	PE(16:0/22:6(4Z,7Z,10Z,13Z,16Z,19Z))	HMDB0008946	9546799	C00350	CCCCCCCCCCCCCCC
346	HMDB0008993	PE(18:0/18:1(9Z))	HMDB0008993	9546742	C00350	CCCCCCCCCCCCCCC
347	HMDB0009002	PE(18:0/20:3(8Z,11Z,14Z))	HMDB0009002	52924903	C00350	CCCCCCCCCCCCCCC
348	HMDB0009003	PE(18:0/20:4(5Z,8Z,11Z,14Z))	HMDB0009003	5289133	C00350	CCCCCCCCCCCCCCC
349	HMDB0009012	PE(18:0/22:6(4Z,7Z,10Z,13Z,16Z,19Z))	HMDB0009012	9546798	C00350	CCCCCCCCCCCCCCC
350	HMDB0009059	PE(18:1(9Z)/18:1(9Z))	HMDB0009059	9546757	C00350	CCCCCCCC/C=C\CC
351	HMDB0005349	NA	NA	NA	NA	NA
352	HMDB0009069	PE(18:1(9Z)/20:4(5Z,8Z,11Z,14Z))	HMDB0009069	52924897	C00350	CCCCCCCC/C=C\CC
353	HMDB0009078	PE(18:1(9Z)/22:6(4Z,7Z,10Z,13Z,16Z,19Z))	HMDB0009078	52922113	C00350	CCCCCCCC/C=C\CC
354	HMDB0009093	PE(18:2(9Z,12Z)/18:2(9Z,12Z))	HMDB0009093	9546812	C00350	CCCCC/C=C\C/C=C
355	HMDB0009102	PE(18:2(9Z,12Z)/20:4(5Z,8Z,11Z,14Z))	HMDB0009102	52924893	C00350	CCCCC/C=C\C/C=C
356	HMDB0010163	PS(18:0/18:1(9Z))	HMDB0010163	59720717	C02737	CCCCCCCCCCCCCCC
357	HMDB0012383	PS(18:0/20:4(5Z,8Z,11Z,14Z))	HMDB0012383	24779545		CCCCCCCCCCCCCCC
358	HMDB0010167	PS(18:0/22:6(4Z,7Z,10Z,13Z,16Z,19Z))	HMDB0010167	24779546	C02737	CCCCCCCCCCCCCCC
359	HMDB0010604	PG(18:0/18:1(9Z))	HMDB0010604	24779551		CCCCCCCCCCCCCCC
360	HMDB0009783	PI(16:0/18:1(9Z))	HMDB0009783		C00626	[H][C@@](COC(=O)CC
361	HMDB0009789	PI(16:0/20:4(5Z,8Z,11Z,14Z))	HMDB0009789		C00626	[H][C@@](COC(=O)CC
362	HMDB0240667	NA	NA	NA	NA	NA
363	HMDB0009809	PI(18:0/18:2(9Z,12Z))	HMDB0009809		C00626	[H][C@@](COC(=O)CC
364	HMDB0009815	PI(18:0/20:4(5Z,8Z,11Z,14Z))	HMDB0009815		C00626	[H][C@@](COC(=O)CC
365	HMDB0009814	PI(18:0/20:3(8Z,11Z,14Z))	HMDB0009814		C00626	[H][C@@](COC(=O)CC
366	HMDB0009821	PI(18:0/22:6(4Z,7Z,10Z,13Z,16Z,19Z))	HMDB0009821		C00626	[H][C@@](COC(=O)CC
367	HMDB0010382	LysoPC(16:0)	HMDB0010382	460602	C04230	CCCCCCCCCCCCCCC
368	HMDB0061702	NA	NA	NA	NA	NA
369	HMDB0010383	LysoPC(16:1(9Z))	HMDB0010383	24779461	C04230	CCCCCC/C=C\CCCC
370	HMDB0010384	LysoPC(18:0)	HMDB0010384	497299	C04230	CCCCCCCCCCCCCCC
371	HMDB0002815	LysoPC(18:1(9Z))	HMDB0002815	16081932	C04230	CCCCCCCC/C=C\CC
372	HMDB0010386	LysoPC(18:2(9Z,12Z))	HMDB0010386	11005824	C04230	CCCCC/C=C\C/C=C
373	HMDB0010390	LysoPC(20:0)	HMDB0010390	24779473	C04230	CCCCCCCCCCCCCCC
374	HMDB0010391	LysoPC(20:1(11Z))	HMDB0010391	52924051	C04230	CCCCCCCC/C=C\CC
375	HMDB0011503	LysoPE(16:0/0:0)	HMDB0011503	9547069		CCCCCCCCCCCCCCC
376	HMDB0011130	LysoPE(18:0/0:0)	HMDB0011130	9547068	C21484	CCCCCCCCCCCCCCC
377	HMDB0011129	LysoPE(0:0/18:0)	HMDB0011129	53480667		CCCCCCCCCCCCCCC
378	HMDB0011506	LysoPE(18:1(9Z)/0:0)	HMDB0011506	9547071		CCCCCCCC/C=C\CC
379	HMDB0011507	LysoPE(18:2(9Z,12Z)/0:0)	HMDB0011507	52925130		CCCCC/C=C\C/C=C
380	HMDB0011517	LysoPE(20:4(5Z,8Z,11Z,14Z)/0:0)	HMDB0011517	42607465		CCCCC/C=C\C/C=C
381	HMDB0011489	LysoPE(0:0/20:5(5Z,8Z,11Z,14Z,17Z))	HMDB0011489	53480938		CC/C=C\C/C=C\C/C
382	HMDB11496	LysoPE(0:0/22:6(4Z,7Z,10Z,13Z,16Z,19Z))	HMDB0011496	53480945		CC/C=C\C/C=C\C/C
383	HMDB0061698	NA	NA	NA	NA	NA
384	HMDB0061694	NA	NA	NA	NA	NA
385	HMDB0240600	NA	NA	NA	NA	NA
386	HMDB0061695	NA	NA	NA	NA	NA
387	HMDB0240261	LysoPI(18:0/0:0)	HMDB0240261			CCCCCCCCCCCCCCC
388	HMDB0061704	NA	NA	NA	NA	NA
389	HMDB0061693	NA	NA	NA	NA	NA
390	HMDB0240597	NA	NA	NA	NA	NA
391	HMDB0013405	PC(o-16:0/18:0)	HMDB0013405	11803170		CCCCCCCCCCCCCCC
392	HMDB0011151	PC(O-16:0/18:2(9Z,12Z))	HMDB0011151	6443157		CCCCCCCCCCCCCCC
393	HMDB0011342	PE(P-16:0/18:1(9Z))	HMDB0011342	52925128		CCCCCCCCCCCCCC/
394	HMDB0011343	PE(P-16:0/18:2(9Z,12Z))	HMDB0011343	52925127		CCCCCCCCCCCCCC/
395	HMDB0011206	PC(P-16:0/16:0)	HMDB0011206	11146967		CCCCCCCCCCCCCCC
396	HMDB0011207	PC(P-16:0/16:1(9Z))	HMDB0011207	52923882		CCCCCCCCCCCCCC/
397	HMDB0011352	PE(P-16:0/20:4(5Z,8Z,11Z,14Z))	HMDB0011352	52925126		CCCCCCCCCCCCCC/
398	HMDB0005780	PE(O-16:1(1Z)/22:6(4Z,7Z,10Z,13Z,16Z,19Z))	HMDB0005780	5283497	C00350	CCCCCCCCCCCCCC/
399	HMDB0007996	PC(16:0/P-18:1(11Z))	HMDB0007996	53478675	C00157	CCCCCCCCCCCCCCC
400	HMDB0011375	PE(P-18:0/18:1(9Z))	HMDB0011375	42607457		CCCCCCCCCCCCCCC
401	HMDB0011376	PE(P-18:0/18:2(9Z,12Z))	HMDB0011376	52925079		CCCCCCCCCCCCCCC
402	HMDB0011220	PC(P-16:0/20:4(5Z,8Z,11Z,14Z))	HMDB0011220	24779388		CCCCCCCCCCCCCC/
403	HMDB0011211	PC(P-16:0/18:2(9Z,12Z))	HMDB0011211	24779386		CCCCCCCCCCCCCC/
404	HMDB0005779	PE(O-18:1(1Z)/20:4(5Z,8Z,11Z,14Z))	HMDB0005779	9547058	C00350	CCCCCCCCCCCCCCC
405	HMDB0011262	PC(P-18:0/22:6(4Z,7Z,10Z,13Z,16Z,19Z))	HMDB0011262	42607430		CCCCCCCCCCCCCCC
406	HMDB0011394	PE(P-18:0/22:6(4Z,7Z,10Z,13Z,16Z,19Z))	HMDB0011394	42607458		CCCCCCCCCCCCCCC
407	HMDB0011149	LysoPC(O-18:0)	HMDB0011149	2733532	C04317	CCCCCCCCCCCCCCC

408	HMDB0011152	PE(P-16:0e/0:0)	HMDB0011152	42607469		CCCCCCCCCCCCC/
409	HMDB0240598	NA	NA	NA	NA	NA
410	HMDB0000126	Glycerol 3-phosphate	HMDB0000126	439162	C00093	C([C@H](COP(=O)(O)
411	HMDB0240316	NA	NA	NA	NA	NA
412	HMDB0011561	MG(14:0/0:0/0:0)	HMDB0011561	10957631		CCCCCCCCCCCCC(
413	HMDB0007102	DG(16:0/18:1(9Z)/0:0)	HMDB0007102	5282283	C13861	CCCCCCCCCCCCCC
414	HMDB0007103	DG(16:0/18:2(9Z,12Z)/0:0)	HMDB0007103	9543695	C00165	CCCCCCCCCCCCCC
415	HMDB0007112	DG(16:0/20:4(5Z,8Z,11Z,14Z)/0:0)	HMDB0007112	9543736	C00165	CCCCCCCCCCCCCC
416	HMDB0007121	DG(16:0/22:6(4Z,7Z,10Z,13Z,16Z,19Z)/0:0)	HMDB0007121	9543827		CCCCCCCCCCCCCC
417	HMDB0007218	DG(18:1(9Z)/18:1(9Z)/0:0)	HMDB0007218	9543716	C00165	CCCCCCCC/C=C\CC
418	HMDB0007219	DG(18:1(9Z)/18:2(9Z,12Z)/0:0)	HMDB0007219	9543722	C00165	CCCCCCCC/C=C\CC
419	HMDB0007248	DG(18:2(9Z,12Z)/18:2(9Z,12Z)/0:0)	HMDB0007248	9543729	C00165	CCCCC/C=C\C/C=C
420	HMDB0007170	DG(18:0/20:4(5Z,8Z,11Z,14Z)/0:0)	HMDB0007170	6438587	C00165	CCCCCCCCCCCCCC
421	HMDB0007228	DG(18:1(9Z)/20:4(5Z,8Z,11Z,14Z)/0:0)	HMDB0007228	9543786	C00165	CCCCCCCC/C=C\CC
422	HMDB0007257	DG(18:2(9Z,12Z)/20:4(5Z,8Z,11Z,14Z)/0:0)	HMDB0007257	9543796	C00165	CCCCC/C=C\C/C=C
423	HMDB0006790	Galactosylglycerol	HMDB0006790	656504	C05401	C([C@@H]1[C@@H]([C
424	HMDB01480	3-Dehydrosphinganine	HMDB0001480	439853	C02934	CCCCCCCCCCCCCC
425	HMDB0000269	Sphinganine	HMDB0000269	91486	C00836	CCCCCCCCCCCCCC
426	HMDB0001383	Sphinganine 1-phosphate	HMDB0001383	644260	C01120	CCCCCCCCCCCCCC
427	HMDB11760	Cer(d18:0/16:0)	HMDB0011760	5283572		CCCCCCCCCCCCCC
428	HMDB0011761	Cer(d18:0/18:0)	HMDB0011761	5283573		CCCCCCCCCCCCCC
429	HMDB0004949	Ceramide (d18:1/16:0)	HMDB0004949	5283564	C00195	CCCCCCCCCCCCCC
430	HMDB0004950	Ceramide (d18:1/18:0)	HMDB0004950	5283565	C00195	CCCCCCCCCCCCCC
431	HMDB0240686	NA	NA	NA	NA	NA
432	HMDB0011773	NA	NA	NA	NA	NA
433	HMDB0240681	NA	NA	NA	NA	NA
434	HMDB0240678	NA	NA	NA	NA	NA
435	HMDB0240683	NA	NA	NA	NA	NA
436	HMDB0240684	NA	NA	NA	NA	NA
437	HMDB0004951	NA	NA	NA	NA	NA
438	HMDB0240682	NA	NA	NA	NA	NA
439	HMDB0240679	NA	NA	NA	NA	NA
440	HMDB0240680	NA	NA	NA	NA	NA
441	HMDB0006750	Lactosylceramide (d18:1/16:0)	HMDB0006750	53477895	C01290	CCCCCCCCCCCCCC
442	HMDB0011594	Lactosylceramide (d18:1/22:0)	HMDB0011594	52921641	C01290	CCCCCCCCCCCCCC
443	HMDB0004872	Lactosylceramide (d18:1/24:1(15Z))	HMDB0004872	20057309	C01290	CCCCCCCCCCCCC/C
444	HMDB0012085	SM(d18:0/14:0)	HMDB0012085	44260138	C00550	CCCCCCCCCCCCCC
445	HMDB0010168	SM(d18:0/16:0)	HMDB0010168	5283591	C00550	CCCCCCCCCCCCCC
446	HMDB0012091	SM(d18:0/22:0)	HMDB0012091	44260132	C00550	CCCCCCCCCCCCCC
447	HMDB0012087	SM(d18:0/18:0)	HMDB0012087	44260130	C00550	CCCCCCCCCCCCCC
448	HMDB0012090	SM(d18:0/20:0)	HMDB0012090	44260131	C00550	CCCCCCCCCCCCCC
449	HMDB0010169	SM(d18:1/16:0)	HMDB0010169	5283590	C00550	CCCCCCCCCCCCCC
450	HMDB0001348	SM(d18:1/18:0)	HMDB0001348	5283588	C00550	CCCCCCCCCCCCCC
451	HMDB0012103	SM(d18:1/22:0)	HMDB0012103	44260125	C00550	CCCCCCCCCCCCCC
452	HMDB0012105	SM(d18:1/23:0)	HMDB0012105	46891684	C00550	CCCCCCCCCCCCCC
453	HMDB0011697	SM(d18:1/24:0)	HMDB0011697	5283595		CCCCCCCCCCCCCC
454	HMDB0240668	NA	NA	NA	NA	NA
455	HMDB0240644	NA	NA	NA	NA	NA
456	HMDB0012097	SM(d18:1/14:0)	HMDB0012097	11433862		CCCCCCCCCCCCC/C
457	HMDB0240637	NA	NA	NA	NA	NA
458	HMDB0240612	NA	NA	NA	NA	NA
459	HMDB0240608	NA	NA	NA	NA	NA
460	HMDB0240617	NA	NA	NA	NA	NA
461	HMDB0240677	NA	NA	NA	NA	NA
462	HMDB0240613	NA	NA	NA	NA	NA
463	HMDB0240638	NA	NA	NA	NA	NA
464	HMDB0240609	NA	NA	NA	NA	NA
465	HMDB0240622	NA	NA	NA	NA	NA
466	HMDB0240620	NA	NA	NA	NA	NA
467	HMDB0012101	SM(d18:1/18:1(9Z))	HMDB0012101	6443882	C00550	CCCCCCCCCCCCC/C
468	HMDB0012102	SM(d18:1/20:0)	HMDB0012102	44260124	C00550	CCCCCCCCCCCCCC
469	HMDB0240610	NA	NA	NA	NA	NA
470	HMDB0240632	NA	NA	NA	NA	NA
471	HMDB0240621	NA	NA	NA	NA	NA
472	HMDB0240611	NA	NA	NA	NA	NA
473	HMDB0240619	NA	NA	NA	NA	NA
474	HMDB0240676	NA	NA	NA	NA	NA
475	HMDB0012104	SM(d18:1/22:1(13Z))	HMDB0012104	52931203	C00550	CCCCCCCCCCCCC/C
476	HMDB0240672	NA	NA	NA	NA	NA
477	HMDB0240670	NA	NA	NA	NA	NA
478	HMDB0240669	NA	NA	NA	NA	NA
479	HMDB0240634	NA	NA	NA	NA	NA
480	HMDB0011696	NA	NA	NA	NA	NA
481	HMDB0240614	NA	NA	NA	NA	NA
482	HMDB0012107	SM(d18:1/24:1(15Z))	HMDB0012107	44260126	C00550	CCCCCCCCCCCCC/C
483	HMDB0240636	NA	NA	NA	NA	NA
484	HMDB0240615	NA	NA	NA	NA	NA
485	HMDB0240671	NA	NA	NA	NA	NA
486	HMDB0240675	NA	NA	NA	NA	NA
487	HMDB0240673	NA	NA	NA	NA	NA
488	HMDB0240674	NA	NA	NA	NA	NA
489	HMDB0000252	Sphingosine	HMDB0000252	5353955	C00319	CCCCCCCCCCCCC/C
490	HMDB0000277	Sphingosine 1-phosphate	HMDB0000277	5353956	C06124	CCCCCCCCCCCCC/C
491	HMDB0000355	3-Hydroxymethylglutaric acid	HMDB0000355	1662	C03761	CC(CC(=O)O)(CC(=O
492	HMDB0000067	Cholesterol	HMDB0000067	5997	C00187	C[C@H](CCCC(C)C)[C
493	HMDB00653	Cholesterol sulfate	HMDB0000653	65076	C18043	C[C@H](CCCC(C)C)[C
494	HMDB0000921	Cholestenone	HMDB0000921	91477	C00599	C[C@H](CCCC(C)C)[C

495	HMDB0002869	Campesterol	HMDB0002869	5283637	C01789	C[C@H](CC[C@H](C)C
496	HMDB0006119	7b-Hydroxycholesterol	HMDB0006119	473141		C[C@H](CCCC(C)C)[C
497	HMDB0000619	Cholic acid	HMDB0000619	221493	C00695	C[C@H](CCC(=O)O)[C
498	HMDB0000138	Glycocholic acid	HMDB0000138	23617285	C01921	C[C@H](CCC(=O)NCC
499	HMDB0000626	Deoxycholic acid	HMDB0000626	222528	C04483	C[C@H](CCC(=O)O)[C
500	HMDB00631	Deoxycholic acid glycine conjugate	HMDB0000631	3035026	C05464	C[C@H](CCC(=O)NCC
501	HMDB0001517	AICAR	HMDB0001517	65110	C04677	C1=NC(=C(N1[C@H]2
502	HMDB0000175	Inosinic acid	HMDB0000175	8582	C00130	C1=NC(=O)C2=C(N1
503	HMDB0000195	Inosine	HMDB0000195	6021	C00294	C1=NC(=O)C2=C(N1
504	HMDB0000157	Hypoxanthine	HMDB0000157	790	C00262	C1=NC2=C(N1)C(=O
505	HMDB0000292	Xanthine	HMDB0000292	1188	C00385	C1=NC2=C(N1)C(=O
506	HMDB0000071	Deoxyinosine	HMDB0000071	65058	C05512	C1[C@@H]([C@H](O[C
507	HMDB0000289	Uric acid	HMDB0000289	1175	C00366	C12=C(NC(=O)N1)NC
508	HMDB0000462	Allantoin	HMDB0000462	204	C01551	C1(C(=O)NC(=O)N1)
509	HMDB0000538	Adenosine triphosphate	HMDB0000538	5957	C00002	C1=NC2=C(C(=N1)N
510	HMDB0001341	ADP	HMDB0001341	6022	C00008	C1=NC2=C(C(=N1)N
511	HMDB0000045	Adenosine monophosphate	HMDB0000045	6083	C00020	C1=NC2=C(C(=N1)N
512	HMDB0000058	Cyclic AMP	HMDB0000058	6076	C00575	C1[C@@H]2[C@H]([C@
513	HMDB0000536	Adenylsuccinic acid	HMDB0000536	440122	C03794	C1=NC2=C(C(=N1)N
514	HMDB0000050	Adenosine	HMDB0000050	60961	C00212	C1=NC2=C(C(=N1)N
515	HMDB0000034	Adenine	HMDB0000034	190	C00147	C1=NC2=C(N1)C(=N
516	HMDB0000905	Deoxyadenosine monophosphate	HMDB0000905	12599	C00360	C1[C@@H]([C@H](O[C
517	HMDB0000912	Succinyladenosine	HMDB0000912	20849086		C1=NC2=C(C(=N1)N
518	HMDB0001397	Guanosine monophosphate	HMDB0001397	6804	C00144	C1=NC2=C(N1[C@H]3
519	HMDB0000133	Guanosine	HMDB0000133	6802	C00387	C1=NC2=C(N1[C@H]3
520	HMDB0000132	Guanine	HMDB0000132	764	C00242	C1=NC2=C(N1)C(=O
521	HMDB0000897	7-Methylguanine	HMDB0000897	11361	C02242	CN1C=NC2=C1C(=O)
522	HMDB0000828	Ureidosuccinic acid	HMDB0000828	93072	C00438	C([C@@H](C(=O)O)N
523	HMDB03349	L-Dihydroorotic acid	HMDB0003349	439216	C00337	C1[C@H](NC(=O)NC1
524	HMDB0000226	Orotic acid	HMDB0000226	967	C00295	C1=C(NC(=O)NC1=C
525	HMDB0000788	Orotidine	HMDB0000788	92751	C01103	C1=C(N(C(=O)NC1=
526	HMDB0000285	Uridine triphosphate	HMDB0000285	6133	C00075	C1=CN(C(=O)NC1=C
527	HMDB0000295	Uridine 5'-diphosphate	HMDB0000295	6031	C00015	C1=CN(C(=O)NC1=C
528	HMDB0000288	Uridine 5'-monophosphate	HMDB0000288	6030	C00105	C1=CN(C(=O)NC1=C
529	HMDB0000296	Uridine	HMDB0000296	6029	C00299	C1=CN(C(=O)NC1=C
530	HMDB0000300	Uracil	HMDB0000300	1174	C00106	C1=CNC(=O)NC1=O
531	HMDB0000767	Pseudouridine	HMDB0000767	15047	C02067	C1=C(C(=O)NC(=O)N
532	HMDB0000884	Ribothymidine	HMDB0000884	445408		CC1=CN(C(=O)NC1=
533	HMDB0000012	Deoxyuridine	HMDB0000012	13712	C00526	C1[C@@H]([C@H](O[C
534	HMDB0000026	Ureidopropionic acid	HMDB0000026	111	C02642	C(CNC(=O)N)C(=O)O
535	HMDB0000056	Beta-Alanine	HMDB0000056	239	C00099	C(CN)C(=O)O
536	HMDB0000095	Cytidine monophosphate	HMDB0000095	8117	C00055	C1=CN(C(=O)N=C1N
537	HMDB0000089	Cytidine	HMDB0000089	6253	C00475	C1=CN(C(=O)N=C1N
538	HMDB0000630	Cytosine	HMDB0000630	597	C00380	C1=C(NC(=O)N=C1)
539	HMDB0001227	5-Thymidylic acid	HMDB0001227	9700	C00364	CC1=CN(C(=O)NC1=
540	HMDB0000273	Thymidine	HMDB0000273	5789	C00214	CC1=CN(C(=O)NC1=
541	HMDB0002166	(S)-b-aminoisobutyric acid	HMDB0002166	439434	C03284	C[C@@H](CN)C(=O)O
542	HMDB61711	Methylphosphate	HMDB0061711	13130		COP(O)(O)=O
543	HMDB0001132	Nicotinic acid mononucleotide	HMDB0001132	53477721	C01185	C1C(C=CC=[N+]1[C@
544	HMDB0001406	Niacinamide	HMDB0001406	936	C00153	C1=CC(=CN=C1)C(=
545	HMDB0000229	Nicotinamide ribotide	HMDB0000229	14180	C00455	C1=CC(=C[N+](=C1)
546	HMDB0000855	Nicotinamide riboside	HMDB0000855	439924	C03150	C1=CC(=C[N+](=C1)
547	HMDB0001179	Nicotinic acid adenine dinucleotide	HMDB0001179	583440	C00857	C1=CC(=C[N+](=C1)
548	HMDB0000699	1-Methylnicotinamide	HMDB0000699	457	C02918	C[N+]1=CC=CC(=C1)
549	HMDB0000875	Trigonelline	HMDB0000875	5570	C01004	C[N+]1=CC=CC(=C1)
550	HMDB0000210	Pantothenic acid	HMDB0000210	6613	C00864	CC(C)(CO)C(C(=O)N
551	HMDB0240294	NA	NA	NA	NA	NA
552	HMDB0000943	NA	NA	NA	NA	NA
553	HMDB0003290	Gulonic acid	HMDB0003290	152304	C00800	C([C@H]([C@@H]([C@
554	HMDB0000030	Biotin	HMDB0000030	171548	C00120	C1[C@H]2[C@@H]([C@
555	HMDB0000121	Folic acid	HMDB0000121	6037	C00504	C1=CC(=CC=C1C(=
556	HMDB0003178	Heme	HMDB0003178	26945	C00032	CC1=C(CCC(O)=O)C
557	HMDB0000235	Thiamine	HMDB0000235	1130	C00378	CC1=C(SC=[N+]1CC2
558	HMDB0002666	Thiamine monophosphate	HMDB0002666	3382778	C01081	CC1=C(SC=[N+]1CC2
559	HMDB0001372	Thiamine pyrophosphate	HMDB0001372	1132	C00068	CC1=C(SC=[N+]1CC2
560	HMDB0000239	Pyridoxine	HMDB0000239	1054	C00314	CC1=NC=C(C(=C1O)
561	HMDB01319	Pyridoxine 5'-phosphate	HMDB0001319	1055	C00627	CC1=NC=C(C(=C1O)
562	HMDB0001431	Pyridoxamine	HMDB0001431	1052	C00534	CC1=NC=C(C(=C1O)
563	HMDB0001555	Pyridoxamine 5'-phosphate	HMDB0001555	1053	C00647	CC1=NC=C(C(=C1O)
564	HMDB0001491	Pyridoxal 5'-phosphate	HMDB0001491	1051	C00018	CC1=NC=C(C(=C1O)
565	HMDB0001545	Pyridoxal	HMDB0001545	1050	C00250	CC1=NC=C(C(=C1O)
566	HMDB0000017	4-Pyridoxic acid	HMDB0000017	6723	C00847	CC1=NC=C(C(=C1O)
567	HMDB0000714	Hippuric acid	HMDB0000714	464	C01586	C1=CC=C(C=C1)C(=
568	HMDB0059724	NA	NA	NA	NA	NA
569	HMDB0240459	NA	NA	NA	NA	NA
570	HMDB0011635	p-Cresol sulfate	HMDB0011635	4615423		CC1=CC=C(C=C1)O
571	HMDB29737	1H-Indole-3-carboxaldehyde	HMDB0029737	10256	C08493	C1=CC=C2C(=C1)C(
572	HMDB0029412	Betonicine	HMDB0029412	164642	C08269	C[N+]1(C[C@@H](C[C
573	HMDB0000625	Gluconic acid	HMDB0000625	10690	C00257	C([C@H]([C@H]([C@@
574	HMDB0003045	Ergothioneine	HMDB0003045	5351619	C05570	C[N+](C)(C)[C@@H](C
575	HMDB0003072	Quinic acid	HMDB0003072	6508	C00296	OC1C[C@@](O)(C[C@
576	HMDB0029422	L-Histidine trimethylbetaine	HMDB0029422	440727	C05575	C[N+](C)(C)C(CC1=C
577	HMDB0033433	(S)-Homostachydrine	HMDB0033433	441447	C08283	C[N+]1(CCCC[C@H]1C
578	HMDB0004827	Proline betaine	HMDB0004827	7016563	C10172	C[N+]1(CCC[C@H]1C(
579	HMDB0001353	2-Keto-3-deoxy-D-gluconic acid	HMDB0001353	194024	C01216	C([C@H]([C@@H](CO)
580	HMDB0029968	Ethyl beta-D-glucopyranoside	HMDB0029968	428040		CCOC1C(C(C(C(O1)C
581	HMDB0001859	Acetaminophen	HMDB0001859	1983	C06804	CC(=O)NC1=CC=C(

582	HMDB0059911	Paracetamol sulfate	HMDB0059911	83939		CC(=O)NC1=CC=C(C
583	HMDB0010316	Acetaminophen glucuronide	HMDB0010316	83944		CC(=O)NC1=CC=C(C
584	HMDB0062547	2-Hydroxyacetaminophen sulfate	HMDB0062547	86290013		CC(=O)NC1=CC=C(C
585	HMDB0062550	NA	NA	NA	NA	NA
586	HMDB0240217	NA	NA	NA	NA	NA
587	HMDB0001925	Ibuprofen	HMDB0001925	3672	C01588	CC(C)CC1=CC=C(C=
588	HMDB0060920	NA	NA	NA	NA	NA
589	HMDB0060564	NA	NA	NA	NA	NA
590	HMDB0014952	Meloxicam	HMDB0014952	54677470	C08169	CC1=CN=C(S1)NC(=
591	HMDB0014611	Quinine	HMDB0014611	3034034	C06526	COC1=CC2=C(C=CN
592	HMDB0042008	NA	NA	NA	NA	NA
593	HMDB0013676	2,6-Dihydroxybenzoic acid	HMDB0013676	9338	C21298	C1=CC(=C(C(=C1)O)
594	HMDB0029415	S-Carboxymethyl-L-cysteine	HMDB0029415	1080		C(C(C(=O)O)N)SCC(
595	HMDB01448	Sulfate	HMDB0001448	1117	C00059	[O-]S(=O)(=O)[O-]
596	HMDB0002520	Beta-Glycerophosphoric acid	HMDB0002520	2526	C02979	C(C(CO)OP(=O)(O)O
597	HMDB0062164	NA	NA	NA	NA	NA

The second step is to check concentration values. For SSP analysis, the concentration must be measured in *umol* for blood and CSF samples. The urinary concentrations must be first converted to *umol/mmol_creatinine* in order to compare with reported concentrations in literature. No missing or negative values are allowed in SSP analysis. The concentration data for QEA analysis is more flexible. Users can upload either the original concentration data or normalized data. Missing or negative values are allowed (coded as *NA*) for QEA.

5 Selection of Metabolite Set Library

Before proceeding to enrichment analysis, a metabolite set library has to be chosen. There are seven built-in libraries offered by MSEA:

- Metabolic pathway associated metabolite sets (*currently contains 99 entries*);
- Disease associated metabolite sets (reported in blood) (*currently contains 344 entries*);
- Disease associated metabolite sets (reported in urine) (*currently contains 384 entries*)
- Disease associated metabolite sets (reported in CSF) (*currently contains 166 entries*)
- Metabolite sets associated with SNPs (*currently contains 4598 entries*)
- Predicted metabolite sets based on computational enzyme knockout model (*currently contains 912 entries*)
- Metabolite sets based on locations (*currently contains 73 entries*)
- Drug pathway associated metabolite sets (*currently contains 461 entries*)

In addition, MSEA also allows user-defined metabolite sets to be uploaded to perform enrichment analysis on arbitrary groups of compounds which researchers want to test. The metabolite set library is simply a two-column comma separated text file with the first column for metabolite set names and the second column for its compound names (**must use HMDB compound name**) separated by "; ". Please note, the built-in libraries are mainly from human studies. The functional grouping of metabolites may not be valid. Therefore, for data from subjects other than human being, users are suggested to upload their self-defined metabolite set libraries for enrichment analysis.

6 Enrichment Analysis

Over Representation Analysis (ORA) is performed when a list of compound names is provided. The list of compound list can be obtained through conventional feature selection methods, or from a clustering algorithm, or from the compounds with abnormal concentrations detected in SSP, to investigate if some biologically meaningful patterns can be identified.

ORA was implemented using the *hypergeometric test* to evaluate whether a particular metabolite set is represented more than expected by chance within the given compound list. One-tailed p values are provided after adjusting for multiple testing. **Figure 2** below summarizes the result.

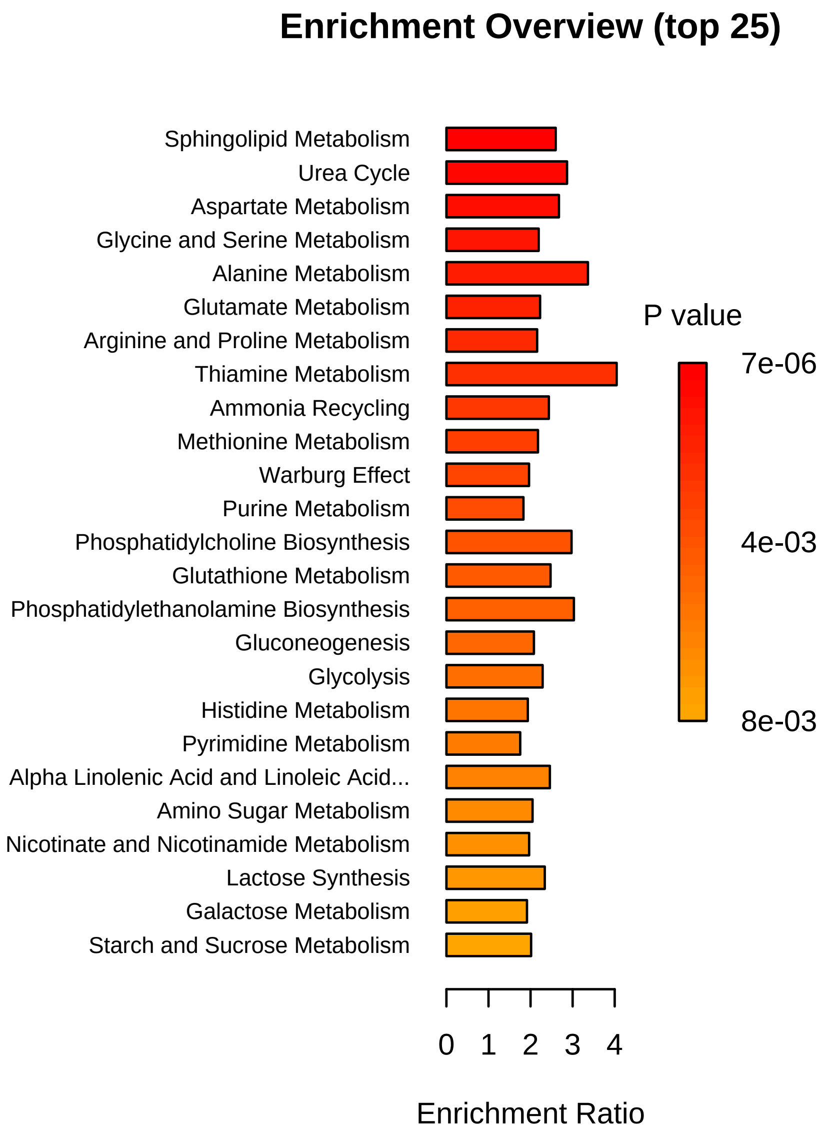

Figure 1: Summary Plot for Over Representation Analysis (ORA)

Table 2: Result from Over Representation Analysis

	total	expected	hits	Raw p	Holm p	FDR
Sphingolipid Metabolism	40	7.70	20	7.36E-06	7.21E-04	4.33E-04
Urea Cycle	29	5.58	16	1.29E-05	1.25E-03	4.33E-04
Aspartate Metabolism	35	6.73	18	1.33E-05	1.27E-03	4.33E-04
Glycine and Serine Metabolism	59	11.40	25	2.10E-05	1.99E-03	5.13E-04
Alanine Metabolism	17	3.27	11	4.35E-05	4.09E-03	8.53E-04
Glutamate Metabolism	49	9.43	21	8.41E-05	7.82E-03	1.37E-03
Arginine and Proline Metabolism	53	10.20	22	1.01E-04	9.32E-03	1.42E-03
Thiamine Metabolism	9	1.73	7	2.25E-04	2.05E-02	2.76E-03
Ammonia Recycling	32	6.16	15	2.85E-04	2.56E-02	3.10E-03
Methionine Metabolism	43	8.27	18	4.03E-04	3.59E-02	3.95E-03
Warburg Effect	58	11.20	22	4.87E-04	4.28E-02	4.34E-03
Purine Metabolism	74	14.20	26	6.02E-04	5.24E-02	4.92E-03
Phosphatidylcholine Biosynthesis	14	2.69	8	1.70E-03	1.47E-01	1.28E-02
Glutathione Metabolism	21	4.04	10	2.73E-03	2.32E-01	1.90E-02
Phosphatidylethanolamine Biosynthesis	12	2.31	7	2.91E-03	2.45E-01	1.90E-02
Gluconeogenesis	35	6.73	14	3.10E-03	2.57E-01	1.90E-02
Glycolysis	25	4.81	11	3.63E-03	2.98E-01	2.06E-02
Histidine Metabolism	43	8.27	16	3.78E-03	3.06E-01	2.06E-02
Pyrimidine Metabolism	59	11.40	20	4.39E-03	3.51E-01	2.26E-02
Alpha Linolenic Acid and Linoleic Acid Metabolism	19	3.66	9	4.69E-03	3.70E-01	2.30E-02
Amino Sugar Metabolism	33	6.35	13	5.10E-03	3.98E-01	2.38E-02
Nicotinate and Nicotinamide Metabolism	37	7.12	14	5.64E-03	4.34E-01	2.51E-02
Lactose Synthesis	20	3.85	9	7.13E-03	5.42E-01	3.03E-02
Galactose Metabolism	38	7.31	14	7.43E-03	5.57E-01	3.03E-02
Starch and Sucrose Metabolism	31	5.96	12	8.36E-03	6.19E-01	3.28E-02
Betaine Metabolism	21	4.04	9	1.04E-02	7.61E-01	3.93E-02
Spermidine and Spermine Biosynthesis	18	3.46	8	1.22E-02	8.78E-01	4.42E-02
Carnitine Synthesis	22	4.23	9	1.48E-02	1.00E+00	4.99E-02
Transfer of Acetyl Groups into Mitochondria	22	4.23	9	1.48E-02	1.00E+00	4.99E-02
Taurine and Hypotaurine Metabolism	12	2.31	6	1.55E-02	1.00E+00	5.07E-02
Cysteine Metabolism	26	5.00	10	1.66E-02	1.00E+00	5.26E-02
Mitochondrial Electron Transport Chain	19	3.66	8	1.76E-02	1.00E+00	5.40E-02
Beta-Alanine Metabolism	34	6.54	12	1.88E-02	1.00E+00	5.59E-02
Methylhistidine Metabolism	4	0.77	3	2.41E-02	1.00E+00	6.80E-02
Glucose-Alanine Cycle	13	2.50	6	2.43E-02	1.00E+00	6.80E-02
Citric Acid Cycle	32	6.16	11	2.96E-02	1.00E+00	7.90E-02
Phosphatidylinositol Phosphate Metabolism	17	3.27	7	2.98E-02	1.00E+00	7.90E-02
Phospholipid Biosynthesis	29	5.58	10	3.67E-02	1.00E+00	9.22E-02
Pentose Phosphate Pathway	29	5.58	10	3.67E-02	1.00E+00	9.22E-02
Valine, Leucine and Isoleucine Degradation	60	11.50	17	5.16E-02	1.00E+00	1.26E-01
Nucleotide Sugars Metabolism	20	3.85	7	7.09E-02	1.00E+00	1.63E-01
Vitamin B6 Metabolism	20	3.85	7	7.09E-02	1.00E+00	1.63E-01
Phenylacetate Metabolism	9	1.73	4	7.50E-02	1.00E+00	1.63E-01
Homocysteine Degradation	9	1.73	4	7.50E-02	1.00E+00	1.63E-01
Lactose Degradation	9	1.73	4	7.50E-02	1.00E+00	1.63E-01
Glycerolipid Metabolism	25	4.81	8	8.85E-02	1.00E+00	1.89E-01
Malate-Aspartate Shuttle	10	1.92	4	1.07E-01	1.00E+00	2.20E-01
Oxidation of Branched Chain Fatty Acids	26	5.00	8	1.08E-01	1.00E+00	2.20E-01
Cardiolipin Biosynthesis	11	2.12	4	1.44E-01	1.00E+00	2.87E-01
Phenylalanine and Tyrosine Metabolism	28	5.39	8	1.52E-01	1.00E+00	2.98E-01
Propanoate Metabolism	42	8.08	11	1.66E-01	1.00E+00	3.18E-01
Threonine and 2-Oxobutanoate Degradation	20	3.85	6	1.70E-01	1.00E+00	3.20E-01
Biotin Metabolism	8	1.54	3	1.86E-01	1.00E+00	3.44E-01
Pyruvate Metabolism	48	9.23	12	1.95E-01	1.00E+00	3.54E-01
Pantothenate and CoA Biosynthesis	21	4.04	6	2.01E-01	1.00E+00	3.59E-01
Beta Oxidation of Very Long Chain Fatty Acids	17	3.27	5	2.15E-01	1.00E+00	3.76E-01
De Novo Triacylglycerol Biosynthesis	9	1.73	3	2.41E-01	1.00E+00	4.15E-01
Selenoamino Acid Metabolism	28	5.39	7	2.83E-01	1.00E+00	4.78E-01
Trehalose Degradation	11	2.12	3	3.57E-01	1.00E+00	5.93E-01
Plasmalogen Synthesis	26	5.00	6	3.83E-01	1.00E+00	6.26E-01
Fructose and Mannose Degradation	32	6.16	7	4.21E-01	1.00E+00	6.65E-01
Sulfate/Sulfite Metabolism	22	4.23	5	4.21E-01	1.00E+00	6.65E-01
Folate Metabolism	29	5.58	6	4.96E-01	1.00E+00	7.72E-01
Lysine Degradation	30	5.77	6	5.32E-01	1.00E+00	8.15E-01
Catecholamine Biosynthesis	20	3.85	4	5.56E-01	1.00E+00	8.26E-01
Riboflavin Metabolism	20	3.85	4	5.56E-01	1.00E+00	8.26E-01
Phytanic Acid Peroxisomal Oxidation	26	5.00	5	5.81E-01	1.00E+00	8.37E-01
Inositol Phosphate Metabolism	26	5.00	5	5.81E-01	1.00E+00	8.37E-01
Pyruvaldehyde Degradation	10	1.92	2	6.02E-01	1.00E+00	8.55E-01
Mitochondrial Beta-Oxidation of Short Chain Saturated Fatty Acids	27	5.19	5	6.17E-01	1.00E+00	8.64E-01
Mitochondrial Beta-Oxidation of Long Chain Saturated Fatty Acids	28	5.39	5	6.52E-01	1.00E+00	8.81E-01
D-Arginine and D-Ornithine Metabolism	11	2.12	2	6.56E-01	1.00E+00	8.81E-01
Glycerol Phosphate Shuttle	11	2.12	2	6.56E-01	1.00E+00	8.81E-01
Estrone Metabolism	24	4.62	4	7.08E-01	1.00E+00	9.30E-01
Bile Acid Biosynthesis	65	12.50	11	7.37E-01	1.00E+00	9.30E-01
Butyrate Metabolism	19	3.66	3	7.40E-01	1.00E+00	9.30E-01

Ethanol Degradation	19	3.66	3	7.40E-01	1.00E+00	9.30E-01
Thyroid hormone synthesis	13	2.50	2	7.47E-01	1.00E+00	9.30E-01
Tryptophan Metabolism	60	11.50	10	7.49E-01	1.00E+00	9.30E-01
Inositol Metabolism	33	6.35	5	7.93E-01	1.00E+00	9.71E-01
Androstenedione Metabolism	24	4.62	3	8.70E-01	1.00E+00	1.00E+00
Degradation of Superoxides	11	2.12	1	9.06E-01	1.00E+00	1.00E+00
Androgen and Estrogen Metabolism	33	6.35	4	9.06E-01	1.00E+00	1.00E+00
Porphyrin Metabolism	40	7.70	5	9.11E-01	1.00E+00	1.00E+00
Steroid Biosynthesis	48	9.23	6	9.26E-01	1.00E+00	1.00E+00
Fatty Acid Biosynthesis	35	6.73	4	9.29E-01	1.00E+00	1.00E+00
Ketone Body Metabolism	13	2.50	1	9.39E-01	1.00E+00	1.00E+00
Fatty acid Metabolism	43	8.27	5	9.40E-01	1.00E+00	1.00E+00
Mitochondrial Beta-Oxidation of Medium Chain Saturated Fatty Acids	27	5.19	2	9.78E-01	1.00E+00	1.00E+00
Ubiquinone Biosynthesis	20	3.85	1	9.87E-01	1.00E+00	1.00E+00
Tyrosine Metabolism	72	13.90	7	9.93E-01	1.00E+00	1.00E+00
Caffeine Metabolism	24	4.62	1	9.94E-01	1.00E+00	1.00E+00
Retinol Metabolism	37	7.12	2	9.97E-01	1.00E+00	1.00E+00
Pterine Biosynthesis	29	5.58	1	9.98E-01	1.00E+00	1.00E+00
Arachidonic Acid Metabolism	69	13.30	5	9.99E-01	1.00E+00	1.00E+00
Steroidogenesis	43	8.27	2	9.99E-01	1.00E+00	1.00E+00
Fatty Acid Elongation In Mitochondria	35	6.73	1	1.00E+00	1.00E+00	1.00E+00

7 Appendix: R Command History

```
 [1] "mSet<-InitDataObjects(\"conc\", \"msetora\", FALSE)"
 [2] "cmpd.vec<-c(\"HMDB0000123\",\"HMDB0000532\",\"HMDB0000271\",\"HMDB0000092\",\"HMDB0000043\",\"H
 [3] "mSet<-Setup.MapData(mSet, cmpd.vec);"
 [4] "mSet<-CrossReferencing(mSet, \"hmdb\");"
 [5] "mSet<-CreateMappingResultTable(mSet)"
 [6] "mSet<-SetMetabolomeFilter(mSet, F);"
 [7] "mSet<-SetCurrentMsetLib(mSet, \"smpdb_pathway\", 2);"
 [8] "mSet<-CalculateHyperScore(mSet)"
 [9] "mSet<-PlotORA(mSet, \"ora_2_\", \"net\", \"png\", 72, width=NA)"
[10] "mSet<-PlotEnrichDotPlot(mSet, \"ora\", \"ora_dot_2_\", \"png\", 72, width=NA)"
[11] "mSet<-CalculateHyperScore(mSet)"
[12] "mSet<-PlotORA(mSet, \"ora_3_\", \"net\", \"png\", 72, width=NA)"
[13] "mSet<-PlotEnrichDotPlot(mSet, \"ora\", \"ora_dot_3_\", \"png\", 72, width=NA)"
[14] "mSet<-CalculateHyperScore(mSet)"
[15] "mSet<-PlotORA(mSet, \"ora_4_\", \"net\", \"png\", 72, width=NA)"
[16] "mSet<-PlotEnrichDotPlot(mSet, \"ora\", \"ora_dot_4_\", \"png\", 72, width=NA)"
[17] "mSet<-CalculateHyperScore(mSet)"
[18] "mSet<-PlotORA(mSet, \"ora_5_\", \"net\", \"png\", 72, width=NA)"
[19] "mSet<-PlotEnrichDotPlot(mSet, \"ora\", \"ora_dot_5_\", \"png\", 72, width=NA)"
[20] "mSet<-SaveTransformedData(mSet)"
[21] "mSet<-PreparePDFReport(mSet, \"guest1426864616232074298\")\n"
```

The report was generated on Wed Oct 26 13:37:23 2022 with R version 4.1.3 (2022-03-10), OS system: Linux, version: 23 20.04.2-Ubuntu SMP Wed Aug 17 02:46:40 UTC 2022 .

Metabolomic Data Analysis with MetaboAnalyst 5.0

Name: guest5859144621155800605

July 6, 2022

1 Background

The Pathway Analysis module combines results from powerful pathway enrichment analysis with pathway topology analysis to help researchers identify the most relevant pathways involved in the conditions under study.

There are many commercial pathway analysis software tools such as Pathway Studio, MetaCore, or Ingenuity Pathway Analysis (IPA), etc. Compared to these commercial tools, the pathway analysis module was specifically developed for metabolomics studies. It uses high-quality KEGG metabolic pathways as the backend knowledgebase. This module integrates many well-established (i.e. univariate analysis, over-representation analysis) methods, as well as novel algorithms and concepts (i.e. Global Test, GlobalAncova, network topology analysis) into pathway analysis. Another feature is a Google-Map style interactive visualization system to deliver the analysis results in an intuitive manner.

2 Data Input

The Pathway Analysis module accepts either a list of compound labels (common names, HMDB IDs or KEGG IDs) with one compound per row, or a compound concentration table with samples in rows and compounds in columns. The second column must be phenotype labels (binary, multi-group, or continuous). The table is uploaded as comma separated values (.csv).

3 Compound Name Matching

The first step is to standardize the compound labels used in user uploaded data. This is a necessary step since these compounds will be subsequently compared with compounds contained in the pathway library. There are three outcomes from the step - exact match, approximate match (for common names only), and no match. Users should click the textbfView button from the approximate matched results to manually select the correct one. Compounds without match will be excluded from the subsequently pathway analysis.

Table 1 shows the conversion results. Note: *1* indicates exact match, *2* indicates approximate match, and *0* indicates no match. A text file contain the result can be found the downloaded file *name_map.csv*

	Query	Match	HMDB	PubChem	KEGG	SMILE[illegible]
1	HMDB0000123	Glycine	HMDB0000123	750	C00037	C(C(=[illegible]
2	HMDB0000532	Acetylglycine	HMDB0000532	10972		CC(=O[illegible]
3	HMDB0000271	Sarcosine	HMDB0000271	1088	C00213	CNCC([illegible]
4	HMDB0000092	Dimethylglycine	HMDB0000092	673	C01026	CN(C)[illegible]
5	HMDB0000043	Betaine	HMDB0000043	247	C00719	C[N+]([illegible]
6	HMDB0000187	L-Serine	HMDB0000187	5951	C00065	C([C@[illegible]
7	HMDB0002931	N-Acetylserine	HMDB0002931	65249		CC(=O[illegible]
8	HMDB0000167	L-Threonine	HMDB0000167	6288	C00188	C[C@H[illegible]
9	HMDB0062557	N-Acetylthreonine	HMDB0062557	152204		C[C@@[illegible]

10	HMDB0011185	O-Phosphothreonine	HMDB0011185	3246323	C12147	C[C@H
11	HMDB0000161	L-Alanine	HMDB0000161	5950	C00041	C[C@@
12	HMDB0000766	N-Acetyl-L-alanine	HMDB0000766	88064		C[C@@
13	HMDB0000191	L-Aspartic acid	HMDB0000191	5960	C00049	C([C@
14	HMDB0000812	N-Acetyl-L-aspartic acid	HMDB0000812	65065	C01042	CC(=O
15	HMDB0000168	L-Asparagine	HMDB0000168	6267	C00152	C([C@
16	HMDB0006028	N-Acetylasparagine	HMDB0006028	99715		CC(=O
17	HMDB32332	Hydroxylated lecithin	HMDB0032332	97663	C03124	C([C@
18	HMDB0000148	L-Glutamic acid	HMDB0000148	33032	C00025	C(CC(
19	HMDB0000641	L-Glutamine	HMDB0000641	5961	C00064	C(CC(
20	HMDB0001138	N-Acetylglutamic acid	HMDB0001138	185	C00624	CC(=O
21	HMDB0006029	N-Acetylglutamine	HMDB0006029	25561		CC(=O
22	HMDB0001344	NA	NA	NA	NA	NA
23	HMDB0061715	NA	NA	NA	NA	NA
24	HMDB0002201	N-Carboxyethyl-g-aminobutyric acid	HMDB0002201	2572		C(CC(
25	HMDB0001301	1-Pyrroline-5-carboxylic acid	HMDB0001301	6642	C03912	C1CC(
26	HMDB0000177	L-Histidine	HMDB0000177	6274	C00135	C1=C(
27	HMDB0000001	1-Methylhistidine	HMDB0000001	92105	C01152	CN1C=
28	HMDB0000479	3-Methylhistidine	HMDB0000479	64969	C01152	CN1C=
29	HMDB0032055	N-Acetylhistidine	HMDB0032055	273260	C02997	CC(=O
30	HMDB0002320	Imidazolelactic acid	HMDB0002320	459122	C05132	C1=CN
31	HMDB0000033	Carnosine	HMDB0000033	439224	C00386	C1=C(
32	HMDB0000194	Anserine	HMDB0000194	112072	C01262	CN1C=
33	HMDB0000870	Histamine	HMDB0000870	774	C00388	C1=C(
34	HMDB0002820	Methylimidazoleacetic acid	HMDB0002820	75810	C05828	CN1C=
35	HMDB04988	Pi-Methylimidazoleacetic acid	HMDB0004988	6451814		CN1C=
36	HMDB0002331	Imidazoleacetic acid riboside	HMDB0002331	440569	C05131	C1=C(
37	HMDB0002024	Imidazoleacetic acid	HMDB0002024	96215	C02835	C1=C(
38	HMDB0003405	D-Lysine	HMDB0003405	866	C00739	C(CCN
39	HMDB0000206	N6-Acetyl-L-lysine	HMDB0000206	92832	C02727	CC(=O
40	HMDB0002038	N(6)-Methyllysine	HMDB0002038	164795	C02728	CNCCC
41	HMDB0013287	Ne,Ne dimethyllysine	HMDB0013287	4478779	C05545	CN(C)
42	HMDB0001325	N6,N6,N6-Trimethyl-L-lysine	HMDB0001325	440120	C03793	C[N+](
43	HMDB0034879	1-[(5-Amino-5-carboxypentyl)amino]-1-deoxyfructose	HMDB0034879	9839580	C16488	NC(CC
44	HMDB0000510	Aminoadipic acid	HMDB0000510	469	C00956	C(CC(
45	HMDB0000070	Pipecolic acid	HMDB0000070	849	C00408	C1CCN
46	HMDB0002284	N-Acetylcadaverine	HMDB0002284	189087		CC(=O
47	HMDB0003355	5-Aminopentanoic acid	HMDB0003355	138	C00431	C(CCN
48	HMDB0000159	L-Phenylalanine	HMDB0000159	6140	C00079	C1=CC
49	HMDB0000512	N-Acetyl-L-phenylalanine	HMDB0000512	74839	C03519	CC(=O
50	HMDB0000158	L-Tyrosine	HMDB0000158	6057	C00082	C1=CC
51	HMDB0000755	Hydroxyphenyllactic acid	HMDB0000755	9378	C03672	C1=CC
52	HMDB0060015	Phenyl hydrogen sulfate	HMDB0060015	74426	C02180	OS(=O
53	HMDB0240317	NA	NA	NA	NA	NA
54	HMDB0000929	L-Tryptophan	HMDB0000929	6305	C00078	C1=CC
55	HMDB0240296	NA	NA	NA	NA	NA
56	HMDB0061115	Lenticin	HMDB0061115	442106	C09213	C[N+](
57	HMDB01200	N'-Formylkynurenine	HMDB0001200	910	C02406	C1=CC
58	HMDB0000684	L-Kynurenine	HMDB0000684	161166	C00328	C1=CC
59	HMDB0000671	Indolelactic acid	HMDB0000671	92904	C02043	C1=CC
60	HMDB0000682	Indoxyl sulfate	HMDB0000682	10258		C1=CC
61	HMDB0000687	L-Leucine	HMDB0000687	6106	C00123	CC(C)
62	HMDB0000695	Ketoleucine	HMDB0000695	70	C00233	CC(C)
63	HMDB0000688	Isovalerylcarnitine	HMDB0000688	6426851		CC(C)
64	HMDB0000754	3-Hydroxyisovaleric acid	HMDB0000754	69362	C20827	CC(C)(
65	HMDB0000172	L-Isoleucine	HMDB0000172	6306	C00407	CC[C@
66	HMDB0061684	N-Acetylisoleucine	HMDB0061684	7036275		CC[C@
67	HMDB0000491	3-Methyl-2-oxovaleric acid	HMDB0000491	47	C00671	CCC(C
68	HMDB0000378	2-Methylbutyroylcarnitine	HMDB0000378	6426901		CCC(C
69	HMDB0002366	Tiglylcarnitine	HMDB0002366	91825636		C/C=C
70	HMDB0001844	Methylsuccinic acid	HMDB0001844	10349	C08645	CC(CC
71	HMDB0000883	L-Valine	HMDB0000883	6287	C00183	CC(C)[
72	HMDB0011757	N-Acetylvaline	HMDB0011757	227752		CC(C)
73	HMDB0000019	Alpha-ketoisovaleric acid	HMDB0000019	49	C00141	CC(C)
74	HMDB0000407	2-Hydroxy-3-methylbutyric acid	HMDB0000407	99823		CC(C)
75	HMDB0000736	Isobutyryl-L-carnitine	HMDB0000736	168379		CC(C)
76	HMDB0000696	L-Methionine	HMDB0000696	6137	C00073	CSCC[
77	HMDB0011745	N-Acetyl-L-methionine	HMDB0011745	6180	C02712	CC(=O
78	HMDB0001015	N-Formyl-L-methionine	HMDB0001015	6995182	C03145	CSCC[
79	HMDB0062174	NA	NA	NA	NA	NA
80	HMDB0002005	Methionine sulfoxide	HMDB0002005	847	C02989	CS(=O
81	HMDB0000939	S-Adenosylhomocysteine	HMDB0000939	439155	C00021	C1=NC
82	HMDB0001087	5-Methylthioribose	HMDB0001087	439904	C03089	CSC[C
83	HMDB0240388	NA	NA	NA	NA	NA
84	HMDB0000742	Homocysteine	HMDB0000742	778	C00155	C(CS)C
85	HMDB0000574	L-Cysteine	HMDB0000574	5862	C00097	C([C@
86	HMDB0001890	Acetylcysteine	HMDB0001890	12035	C06809	CC(=O
87	HMDB0029432	(S)C(S)S-S-Methylcysteine sulfoxide	HMDB0029432	82142		CS(=O
88	HMDB0000996	3-Sulfinoalanine	HMDB0000996	439270	C00606	C([C@
89	HMDB0000965	Hypotaurine	HMDB0000965	107812	C00519	C(CS(
90	HMDB0000251	Taurine	HMDB0000251	1123	C00245	C(CS(
91	HMDB0240253	NA	NA	NA	NA	NA
92	HMDB0002757	Cysteic acid	HMDB0002757	25701	C00506	C(C(C(
93	HMDB0000517	L-Arginine	HMDB0000517	6322	C00062	C(C[C
94	HMDB0000294	Urea	HMDB0000294	2447	C00086	C(=O)(
95	HMDB0000214	Ornithine	HMDB0000214	6262	C00077	C(C[C
96	HMDB0000323	3-Amino-2-piperidone	HMDB0000323	5200225		C1CC(

97	HMDB0004225	2-Oxoarginine	HMDB0004225	558	C03771	C(CC(=
98	HMDB0000904	Citrulline	HMDB0000904	9750	C00327	C(C[C@
99	HMDB0000162	NA	NA	NA	NA	NA
100	HMDB0003411	D-Proline	HMDB0003411	8988	C00763	C1C[C@
101	HMDB0003334	NA	NA	NA	NA	NA
102	HMDB0001539	Asymmetric dimethylarginine	HMDB0001539	123831	C03626	CN(C)C
103	HMDB0004620	N-a-Acetyl-L-arginine	HMDB0004620	67427		CC(=O
104	HMDB0000725	4-Hydroxyproline	HMDB0000725	5810	C01157	C1[C@H
105	HMDB0094696	N-Methyl-proline	HMDB0094696	643474		CN1CC
106	HMDB0240365	NA	NA	NA	NA	NA
107	HMDB0000128	Guanidoacetic acid	HMDB0000128	763	C00581	C(C(=O
108	HMDB0000064	Creatine	HMDB0000064	586	C00300	CN(CC
109	HMDB0000562	Creatinine	HMDB0000562	588	C00791	CN1CC
110	HMDB0001511	Phosphocreatine	HMDB0001511	587	C02305	CN(CC
111	HMDB0002064	N-Acetylputrescine	HMDB0002064	122356	C02714	CC(=O
112	HMDB0001257	Spermidine	HMDB0001257	1102	C00315	C(CCN
113	HMDB0001276	NA	NA	NA	NA	NA
114	HMDB0002189	N8-Acetylspermidine	HMDB0002189	123689	C01029	CC(=O
115	HMDB0001256	Spermine	HMDB0001256	1103	C00750	C(CCN
116	HMDB0001186	N1-Acetylspermine	HMDB0001186	916	C02567	CC(=O
117	HMDB0001173	5'-Methylthioadenosine	HMDB0001173	439176	C00170	CSC[C@
118	HMDB0001522	Methylguanidine	HMDB0001522	10111	C02294	CN=C(
119	HMDB0003464	4-Guanidinobutanoic acid	HMDB0003464	500	C01035	C(CC(=
120	HMDB0000125	NA	NA	NA	NA	NA
121	HMDB0003337	Oxidized glutathione	HMDB0003337	975	C00127	C(CC(=
122	HMDB0000656	Cysteineglutathione disulfide	HMDB0000656	53477713		C(CC(=
123	HMDB0001066	S-Lactoylglutathione	HMDB0001066	440018	C03451	C[C@H
124	HMDB0000078	Cysteinylglycine	HMDB0000078	439498	C01419	C([C@@
125	HMDB0000267	Pyroglutamic acid	HMDB0000267	7405	C01879	C1CC(=
126	HMDB0000729	NA	NA	NA	NA	NA
127	HMDB00008	2-Hydroxybutyric acid	HMDB0000008	11266	C05984	CCC(C
128	HMDB0005765	Ophthalmic acid	HMDB0005765	7018721	C21016	CC[C@
129	HMDB0240578	NA	NA	NA	NA	NA
130	HMDB0001049	gamma-Glutamylcysteine	HMDB0001049	123938	C00669	C(CC(=
131	HMDB0011737	gamma-Glutamylglutamic acid	HMDB0011737	92865	C05282	C(CC(=
132	HMDB0011738	N2-gamma-Glutamylglutamine	HMDB0011738	150914	C05283	C(CC(=
133	HMDB0011170	gamma-Glutamylisoleucine	HMDB0011170	22885096		CC[C@
134	HMDB0011171	gamma-Glutamylleucine	HMDB0011171	4524287		CC(C)C
135	HMDB0029159	gamma-Glutamylthreonine	HMDB0029159	53861142		C[C@H
136	HMDB0011172	gamma-Glutamylvaline	HMDB0011172	7015683		CC(C)[
137	HMDB0028818	Glutamylglutamic acid	HMDB0028818	439500	C01425	C(CC(=
138	HMDB0028749	Aspartyl-Aspartate	HMDB0028749	471583		NC(CC
139	HMDB0000759	Glycylleucine	HMDB0000759	92843	C02155	CC(C)C
140	HMDB0000721	Glycylproline	HMDB0000721	79101		C1CC(N
141	HMDB0028854	Glycyl-Valine	HMDB0028854	97417		CC(C)C
142	HMDB0028988	Phenylalanyl-Alanine	HMDB0028988	5488196		CC(NC
143	HMDB0029010	Prolyl-Alanine	HMDB0029010	6347578		CC(NC
144	HMDB0011178	Prolylglycine	HMDB0011178	98206		C1CC(N
145	HMDB0011180	L-prolyl-L-proline	HMDB0011180	263469		C1CC(N
146	HMDB0029127	Valyl-Glycine	HMDB0029127	6993111		CC(C)C
147	HMDB0029131	Valyl-Leucine	HMDB0029131	6993118		CC(C)C
148	HMDB0006344	Alpha-N-Phenylacetyl-L-glutamine	HMDB0006344	92258	C04148	C1=CC
149	HMDB0000821	Phenylacetylglycine	HMDB0000821	68144	C05598	C1=CC
150	HMDB0000122	D-Glucose	HMDB0000122	5793	C00221	C([C@@
151	HMDB0001401	Glucose 6-phosphate	HMDB0001401	5958	C00092	C([C@@
152	HMDB0003514	NA	NA	NA	NA	NA
153	HMDB0001058	Fructose 1,6-bisphosphate	HMDB0001058	445557	C05378	C([C@@
154	HMDB0001294	2,3-Diphosphoglyceric acid	HMDB0001294	186004	C01159	C([C@H
155	HMDB0001473	Dihydroxyacetone phosphate	HMDB0001473	668	C00111	C(C(=O
156	HMDB0000362	2-Phosphoglyceric acid	HMDB0000362	59		C(C(C(
157	HMDB0000807	3-Phosphoglyceric acid	HMDB0000807	724	C00597	C(C(C(
158	HMDB0000263	Phosphoenolpyruvic acid	HMDB0000263	1005	C00074	C=C(C
159	HMDB0000243	Pyruvic acid	HMDB0000243	1060	C00022	CC(=O
160	HMDB0000190	L-Lactic acid	HMDB0000190	61503	C00186	C[C@@
161	HMDB0006372	NA	NA	NA	NA	NA
162	HMDB0000139	Glyceric acid	HMDB0000139	439194	C00258	C([C@H
163	HMDB0001316	6-Phosphogluconic acid	HMDB0001316	91493	C00345	C([C@H
164	HMDB0000280	Phosphoribosyl pyrophosphate	HMDB0000280	7339	C00119	C([C@@
165	HMDB0001068	D-Sedoheptulose 7-phosphate	HMDB0001068	22833559	C05382	C([C@@
166	HMDB0002917	NA	NA	NA	NA	NA
167	HMDB0000508	NA	NA	NA	NA	NA
168	HMDB0001851	NA	NA	NA	NA	NA
169	HMDB0000568	D-Arabitol	HMDB0000568	827	C01904	C(C(C(
170	HMDB0000867	Ribonic acid	HMDB0000867	5460677	C01685	C([C@H
171	HMDB0000621	NA	NA	NA	NA	NA
172	HMDB0000751	NA	NA	NA	NA	NA
173	HMDB0001644	NA	NA	NA	NA	NA
174	HMDB0003371	L-Ribulose	HMDB0003371	439204	C00508	C1[C@H
175	HMDB0000539	Arabinonic acid	HMDB0000539	122045	C00878	C([C@H
176	HMDB0060255	L-Lyxonate	HMDB0060255	644110	C05412	OC[C@
177	HMDB0000258	Sucrose	HMDB0000258	5988	C00089	C([C@@
178	HMDB0000660	D-Fructose	HMDB0000660	439709	C02336	C([C@@
179	HMDB0000247	NA	NA	NA	NA	NA
180	HMDB0000765	Mannitol	HMDB0000765	6251	C00392	C([C@H
181	HMDB0000169	D-Mannose	HMDB0000169	18950	C00936	C([C@@
182	HMDB0000565	Galactonic acid	HMDB0000565	128869	C00880	C([C@H
183	HMDB0000286	Uridine diphosphate glucose	HMDB0000286	53477679	C00029	C1=CN

184	HMDB0000302	Uridine diphosphategalactose	HMDB0000302	18068	C00052	C1=CN
185	HMDB0000935	Uridine diphosphate glucuronic acid	HMDB0000935	17473	C00167	C1=CN
186	HMDB0000304	NA	NA	NA	NA	NA
187	HMDB0000290	Uridine diphosphate-N-acetylglucosamine	HMDB0000290	9547196	C00043	CC(=O
188	HMDB0000230	N-Acetylneuraminic acid	HMDB0000230	445063	C19910	CC(=O
189	HMDB0000489	Aspartylglycosamine	HMDB0000489	123826	C04540	CC(=O
190	HMDB0000613	Erythronic acid	HMDB0000613	2781043		C([C@
191	HMDB0000212	NA	NA	NA	NA	NA
192	HMDB0000215	N-Acetyl-D-glucosamine	HMDB0000215	439174	C00140	CC(=O
193	HMDB0240347	NA	NA	NA	NA	NA
194	HMDB0000094	Citric acid	HMDB0000094	311	C00158	C(C(=
195	HMDB0000958	NA	NA	NA	NA	NA
196	HMDB000072	NA	NA	NA	NA	NA
197	HMDB0001874	D-threo-Isocitric acid	HMDB0001874	5318532	C00451	C([C@
198	HMDB0000208	Oxoglutaric acid	HMDB0000208	51	C00026	C(CC(
199	HMDB0061717	NA	NA	NA	NA	NA
200	HMDB0000254	Succinic acid	HMDB0000254	1110	C00042	C(CC(
201	HMDB0000134	Fumaric acid	HMDB0000134	444972	C00122	C(=C/
202	HMDB0000744	NA	NA	NA	NA	NA
203	HMDB0031518	NA	NA	NA	NA	NA
204	HMDB0000156	L-Malic acid	HMDB0000156	222656	C00149	C([C@
205	HMDB0001494	Acetylphosphate	HMDB0001494	186	C00227	CC(=O
206	HMDB0001429	Phosphate	HMDB0001429	57424078	C00009	[O-]P(=
207	HMDB0002095	Malonylcarnitine	HMDB0002095	22833583		C[N+](
208	HMDB0000691	Malonic acid	HMDB0000691	867	C04025	C(C(=
209	HMDB0000535	Caproic acid	HMDB0000535	8892	C01585	CCCC
210	HMDB0000826	Pentadecanoic acid	HMDB0000826	13849	C16537	CCCC
211	HMDB0000220	Palmitic acid	HMDB0000220	985	C00249	CCCC
212	HMDB0002259	Heptadecanoic acid	HMDB0002259	10465		CCCC
213	HMDB0000827	Stearic acid	HMDB0000827	5281	C01530	CCCC
214	HMDB0000772	Nonadecanoic acid	HMDB0000772	12591	C16535	CCCC
215	HMDB0002212	Arachidic acid	HMDB0002212	10467	C06425	CCCC
216	HMDB0002000	Myristoleic acid	HMDB0002000	5281119	C08322	CCCC/
217	HMDB0003229	Palmitoleic acid	HMDB0003229	5312427	C08362	CCCC
218	HMDB0060038	10Z-Heptadecenoic acid	HMDB0060038	5312435		CCCC
219	HMDB0000207	NA	NA	NA	NA	NA
220	HMDB0000573	NA	NA	NA	NA	NA
221	HMDB0003231	NA	NA	NA	NA	NA
222	HMDB0240219	cis-Vaccenic acid	HMDB0240219	5282761	C08367	CCCC
223	HMDB0013622	Nonadeca-10(Z)-enoic acid	HMDB0013622	5312513	C00174	CCCC
224	HMDB0002231	NA	NA	NA	NA	NA
225	HMDB0062436	9Z-Eicosenoic acid	HMDB0062436	5282767		[H]\C(
226	HMDB0002068	Erucic acid	HMDB0002068	5281116	C08316	CCCC
227	HMDB0000560	5,8-Tetradecadienoic acid	HMDB0000560	5312409		CCCC
228	HMDB0006547	Stearidonic acid	HMDB0006547	5312508	C16300	CC/C=
229	HMDB0001999	Eicosapentaenoic acid	HMDB0001999	446284	C06428	CC/C=
230	HMDB0001976	NA	NA	NA	NA	NA
231	HMDB0006528	Docosapentaenoic acid	HMDB0006528	5497182	C16513	CC/C=
232	HMDB0002183	Docosahexaenoic acid	HMDB0002183	445580	C06429	CC/C=
233	HMDB0002823	Docosatrienoic acid	HMDB0002823	5312557		CC/C=
234	HMDB0000477	7Z,10Z-Hexadecadienoic acid	HMDB0000477	13932172		CCCC
235	HMDB0000673	NA	NA	NA	NA	NA
236	HMDB0006270	Linoelaidic acid	HMDB0006270	5282457		CCCC
237	HMDB0001388	NA	NA	NA	NA	NA
238	HMDB0003073	Gamma-Linolenic acid	HMDB0003073	5280933	C06426	CCCC
239	HMDB0005060	Eicosadienoic acid	HMDB0005060	6439848	C16525	CCCC
240	HMDB0002925	8,11,14-Eicosatrienoic acid	HMDB0002925	5280581	C03242	CCCC
241	HMDB0001043	Arachidonic acid	HMDB0001043	444899	C00219	CCCC
242	HMDB0002226	Adrenic acid	HMDB0002226	5497181	C16527	CCCC
243	HMDB0001976	Docosapentaenoic acid (22n-6)	HMDB0001976	6441454		CCCC
244	HMDB0061714	Docosadienoate (22:2n6)	HMDB0061714	5282807	C16533	CCCC
245	HMDB0010378	5,8,11-Eicosatrienoic acid	HMDB0010378	5312531		CCCC
246	HMDB0061859	Methyl hexadecanoic acid	HMDB0061859	8181	C16995	CCCC
247	HMDB0037397	xi-17-Methyloctadecanoic acid	HMDB0037397	3083779		CC(C)
248	HMDB0000661	Glutaric acid	HMDB0000661	743	C00489	C(CC(
249	HMDB0059655	2-Hydroxyglutarate	HMDB0059655	43	C02630	OC(CC
250	HMDB0000176	Maleic acid	HMDB0000176	444972	C01384	C(=C\
251	HMDB0000623	Dodecanedioic acid	HMDB0000623	12736	C02678	C(CCC
252	HMDB0000824	Propionylcarnitine	HMDB0000824	107738	C03017	CCC(=
253	HMDB0013034	Palmitoylglycine	HMDB0013034	151008		CCCC
254	HMDB0000201	L-Acetylcarnitine	HMDB0000201	7045767	C02571	CC(=O
255	HMDB0000756	L-Hexanoylcarnitine	HMDB0000756	3246938		CCCC
256	HMDB0000791	L-Octanoylcarnitine	HMDB0000791	11953814	C02838	CCCC
257	HMDB0000651	Decanoylcarnitine	HMDB0000651	10245190		CCCC
258	HMDB000225	NA	NA	NA	NA	NA
259	HMDB0005066	Tetradecanoylcarnitine	HMDB0005066	53477791		CCCC
260	HMDB0062517	NA	NA	NA	NA	NA
261	HMDB0000222	L-Palmitoylcarnitine	HMDB0000222	11953816	C02990	CCCC
262	HMDB0006210	Heptadecanoyl carnitine	HMDB0006210	53477803		CCCC
263	HMDB0000848	Stearoylcarnitine	HMDB0000848	52922056		CCCC
264	HMDB0006460	Arachidyl carnitine	HMDB0006460	53477833		CCCC
265	HMDB0062468	NA	NA	NA	NA	NA
266	HMDB0240665	NA	NA	NA	NA	NA
267	HMDB0006347	Hexacosanoyl carnitine	HMDB0006347	53477828		CCCC
268	HMDB0013205	9-Decenoylcarnitine	HMDB0013205	53481651		C[N+](
269	HMDB13326	trans-2-Dodecenoylcarnitine	HMDB0013326	53481671		CCCC
270	HMDB0240588	NA	NA	NA	NA	NA

271	HMDB0013207	9-Hexadecenoylcarnitine	HMDB0013207	53481653		CCCCC
272	HMDB0005065	Oleoylcarnitine	HMDB0005065	46907933		CCCCC
273	HMDB0006509	Nervonyl carnitine	HMDB0006509	29385		CCC[N
274	HMDB0006469	Linoleyl carnitine	HMDB0006469	6450015		CCCCC
275	HMDB0006455	Arachidonyl carnitine	HMDB0006455	53477832		CCCCC
276	HMDB0013127	Hydroxybutyrylcarnitine	HMDB0013127	53481617		CC(CC
277	HMDB0061636	NA	NA	NA	NA	NA
278	HMDB0013336	3-Hydroxyhexadecanoylcarnitine	HMDB0013336	53481691		CCCCC
279	HMDB0001161	4-Trimethylammoniobutanoic acid	HMDB0001161	134	C01181	C[N+](
280	HMDB0000062	L-Carnitine	HMDB0000062	2724480	C00318	C[N+](
281	HMDB0000011	NA	NA	NA	NA	NA
282	HMDB0000357	NA	NA	NA	NA	NA
283	HMDB0000442	(S)-3-Hydroxybutyric acid	HMDB0000442	94318	C03197	C[C@@
284	HMDB0240592	NA	NA	NA	NA	NA
285	HMDB0240596	NA	NA	NA	NA	NA
286	HMDB0062549	2-Hydroxystearic acid	HMDB0062549	439887	C03045	CCCCC
287	HMDB0004667	NA	NA	NA	NA	NA
288	HMDB0004670	Alpha-dimorphecolic acid	HMDB0004670	5312830	C14767	CCCCC
289	HMDB0002453	4-Deoxythreonic acid	HMDB0002453	10964471		C[C@H
290	HMDB0000498	4-Deoxyerythronic acid	HMDB0000498	13120901		C[C@H
291	HMDB0000360	2,4-Dihydroxybutanoic acid	HMDB0000360	192742		C(CO)C
292	HMDB0002088	N-Oleoylethanolamine	HMDB0002088	5283454	C20792	CCCCC
293	HMDB0002100	Palmitoylethanolamide	HMDB0002100	4671	C16512	CCCCC
294	HMDB0004080	Anandamide	HMDB0004080	5281969	C11695	CCCCC
295	HMDB0012252	Linoleoyl ethanolamide	HMDB0012252	5283446		CCCCC
296	HMDB0000211	myo-Inositol	HMDB0000211		C00137	O[C@H
297	HMDB0000097	Choline	HMDB0000097	305	C00114	C[N+](
298	HMDB0001565	Phosphorylcholine	HMDB0001565	8691	C00588	C[N+](
299	HMDB0001413	Citicoline	HMDB0001413	13804	C00307	C[N+](
300	HMDB0000086	Glycerophosphocholine	HMDB0000086	71920	C00670	C[N+](
301	HMDB0000224	O-Phosphoethanolamine	HMDB0000224	1015	C00346	C(COP
302	HMDB0001564	CDP-Ethanolamine	HMDB0001564	123727	C00570	C1=CN
303	HMDB0000114	Glycerylphosphorylethanolamine	HMDB0000114	22833510	C01233	C(CO)N
304	HMDB0000925	Trimethylamine N-oxide	HMDB0000925	1145	C01104	C[N+](
305	HMDB0007869	PC(14:0/16:0)	HMDB0007869	129657	C00157	CCCCC
306	HMDB0007883	PC(14:0/20:4(5Z,8Z,11Z,14Z))	HMDB0007883	24778634	C00157	CCCCC
307	HMDB0007940	PC(15:0/18:2(9Z,12Z))	HMDB0007940	24778664	C00157	CCCCC
308	HMDB0007949	PC(15:0/20:4(5Z,8Z,11Z,14Z))	HMDB0007949	52922330	C00157	CCCCC
309	HMDB0007958	PC(15:0/22:6(4Z,7Z,10Z,13Z,16Z,19Z))	HMDB0007958	52922342	C00157	CCCCC
310	HMDB0007967	PC(16:0/15:0)	HMDB0007967	24778680	C00157	CCCCC
311	HMDB0000564	PC(16:0/16:0)	HMDB0000564	452110	C00157	CCCCC
312	HMDB0007969	PC(16:0/16:1(9Z))	HMDB0007969	6443788	C00157	CCCCC
313	HMDB0007970	PC(16:0/18:0)	HMDB0007970	24778686	C00157	CCCCC
314	HMDB0007972	PC(16:0/18:1(9Z))	HMDB0007972	5497103	C00157	CCCCC
315	HMDB0007973	PC(16:0/18:2(9Z,12Z))	HMDB0007973	5287971	C00157	CCCCC
316	HMDB0007982	PC(16:0/20:4(5Z,8Z,11Z,14Z))	HMDB0007982	10747814	C00157	CCCCC
317	HMDB0007984	PC(16:0/20:5(5Z,8Z,11Z,14Z,17Z))	HMDB0007984	24778723	C00157	CCCCC
318	HMDB0007991	PC(16:0/22:6(4Z,7Z,10Z,13Z,16Z,19Z))	HMDB0007991	6441886	C00157	CCCCC
319	HMDB0008006	PC(16:1(9Z)/18:2(9Z,12Z))	HMDB0008006	24778768	C00157	CCCCC
320	HMDB0008008	PC(16:1(9Z)/18:3(9Z,12Z,15Z))	HMDB0008008	52922452	C00157	CCCCC
321	HMDB0008036	PC(18:0/18:0)	HMDB0008036	94190	C00157	CCCCC
322	HMDB0008038	PC(18:0/18:1(9Z))	HMDB0008038	24778825	C00157	CCCCC
323	HMDB0008045	NA	NA	NA	NA	NA
324	HMDB0008143	PC(18:2(9Z,12Z)/20:0)	HMDB0008143	52922739	C00157	CCCCC
325	HMDB0008047	PC(18:0/20:3(8Z,11Z,14Z))	HMDB0008047	24778857	C00157	CCCCC
326	HMDB0008046	PC(18:0/20:3(5Z,8Z,11Z))	HMDB0008046	24778855	C00157	CCCCC
327	HMDB0008048	PC(18:0/20:4(5Z,8Z,11Z,14Z))	HMDB0008048	16219824	C00157	CCCCC
328	HMDB0008054	PC(18:0/22:4(7Z,10Z,13Z,16Z))	HMDB0008054	24778868	C00157	CCCCC
329	HMDB0008056	PC(18:0/22:5(7Z,10Z,13Z,16Z,19Z))	HMDB0008056	24778873	C00157	CCCCC
330	HMDB0008055	PC(18:0/22:5(4Z,7Z,10Z,13Z,16Z))	HMDB0008055	24778871	C00157	CCCCC
331	HMDB0008057	PC(18:0/22:6(4Z,7Z,10Z,13Z,16Z,19Z))	HMDB0008057	24778876	C00157	CCCCC
332	HMDB0008105	PC(18:1(9Z)/18:2(9Z,12Z))	HMDB0008105	24778939	C00157	CCCCC
333	HMDB0008123	PC(18:1(9Z)/22:6(4Z,7Z,10Z,13Z,16Z,19Z))	HMDB0008123	24778955	C00157	CCCCC
334	HMDB0008138	PC(18:2(9Z,12Z)/18:2(9Z,12Z))	HMDB0008138	5288075	C00157	CCCCC
335	HMDB0008141	PC(18:2(9Z,12Z)/18:3(9Z,12Z,15Z))	HMDB0008141	52922731	C00157	CCCCC
336	HMDB0008147	PC(18:2(9Z,12Z)/20:4(5Z,8Z,11Z,14Z))	HMDB0008147	24778979	C00157	CCCCC
337	HMDB0008279	PC(20:0/20:4(5Z,8Z,11Z,14Z))	HMDB0008279	24779048	C00157	CCCCC
338	HMDB0008923	PE(16:0/16:0)	HMDB0008923	445468	C00350	CCCCC
339	HMDB0008924	PE(16:0/16:1(9Z))	HMDB0008924	52924925	C00350	CCCCC
340	HMDB08925	PE(16:0/18:0)	HMDB0008925	5326793	C00350	CCCCC
341	HMDB0005320	NA	NA	NA	NA	NA
342	HMDB0005322	NA	NA	NA	NA	NA
343	HMDB0005323	NA	NA	NA	NA	NA
344	HMDB0008939	PE(16:0/20:5(5Z,8Z,11Z,14Z,17Z))	HMDB0008939	52924919	C00350	CCCCC
345	HMDB0008946	PE(16:0/22:6(4Z,7Z,10Z,13Z,16Z,19Z))	HMDB0008946	9546799	C00350	CCCCC
346	HMDB0008993	PE(18:0/18:1(9Z))	HMDB0008993	9546742	C00350	CCCCC
347	HMDB0009002	PE(18:0/20:3(8Z,11Z,14Z))	HMDB0009002	52924903	C00350	CCCCC
348	HMDB0009003	PE(18:0/20:4(5Z,8Z,11Z,14Z))	HMDB0009003	5289133	C00350	CCCCC
349	HMDB0009012	PE(18:0/22:6(4Z,7Z,10Z,13Z,16Z,19Z))	HMDB0009012	9546798	C00350	CCCCC
350	HMDB0009059	PE(18:1(9Z)/18:1(9Z))	HMDB0009059	9546757	C00350	CCCCC
351	HMDB0005349	NA	NA	NA	NA	NA
352	HMDB0009069	PE(18:1(9Z)/20:4(5Z,8Z,11Z,14Z))	HMDB0009069	52924897	C00350	CCCCC
353	HMDB0009078	PE(18:1(9Z)/22:6(4Z,7Z,10Z,13Z,16Z,19Z))	HMDB0009078	52922113	C00350	CCCCC
354	HMDB0009093	PE(18:2(9Z,12Z)/18:2(9Z,12Z))	HMDB0009093	9546812	C00350	CCCCC
355	HMDB0009102	PE(18:2(9Z,12Z)/20:4(5Z,8Z,11Z,14Z))	HMDB0009102	52924893	C00350	CCCCC
356	HMDB0010163	PS(18:0/18:1(9Z))	HMDB0010163	59720717	C02737	CCCCC
357	HMDB0012383	PS(18:0/20:4(5Z,8Z,11Z,14Z))	HMDB0012383	24779545		CCCCC

358	HMDB0010167		PS(18:0/22:6(4Z,7Z,10Z,13Z,16Z,19Z))	HMDB0010167	24779546	C02737	CCCCC
359	HMDB0010604		PG(18:0/18:1(9Z))	HMDB0010604	24779551		CCCCC
360	HMDB0009783		PI(16:0/18:1(9Z))	HMDB0009783		C00626	[H][C@
361	HMDB0009789		PI(16:0/20:4(5Z,8Z,11Z,14Z))	HMDB0009789		C00626	[H][C@
362	HMDB0240667		NA	NA	NA	NA	NA
363	HMDB0009809		PI(18:0/18:2(9Z,12Z))	HMDB0009809		C00626	[H][C@
364	HMDB0009815		PI(18:0/20:4(5Z,8Z,11Z,14Z))	HMDB0009815		C00626	[H][C@
365	HMDB0009814		PI(18:0/20:3(8Z,11Z,14Z))	HMDB0009814		C00626	[H][C@
366	HMDB0009821		PI(18:0/22:6(4Z,7Z,10Z,13Z,16Z,19Z))	HMDB0009821		C00626	[H][C@
367	HMDB0010382		LysoPC(16:0)	HMDB0010382	460602	C04230	CCCCC
368	HMDB0061702		NA	NA	NA	NA	NA
369	HMDB0010383		LysoPC(16:1(9Z))	HMDB0010383	24779461	C04230	CCCCC
370	HMDB0010384		LysoPC(18:0)	HMDB0010384	497299	C04230	CCCCC
371	HMDB0002815		LysoPC(18:1(9Z))	HMDB0002815	16081932	C04230	CCCCC
372	HMDB0010386		LysoPC(18:2(9Z,12Z))	HMDB0010386	11005824	C04230	CCCCC
373	HMDB0010390		LysoPC(20:0)	HMDB0010390	24779473	C04230	CCCCC
374	HMDB0010391		LysoPC(20:1(11Z))	HMDB0010391	52924051	C04230	CCCCC
375	HMDB0011503		LysoPE(16:0/0:0)	HMDB0011503	9547069		CCCCC
376	HMDB0011130		LysoPE(18:0/0:0)	HMDB0011130	9547068	C21484	CCCCC
377	HMDB0011129		LysoPE(0:0/18:0)	HMDB0011129	53480667		CCCCC
378	HMDB0011506		LysoPE(18:1(9Z)/0:0)	HMDB0011506	9547071		CCCCC
379	HMDB0011507		LysoPE(18:2(9Z,12Z)/0:0)	HMDB0011507	52925130		CCCCC
380	HMDB0011517		LysoPE(20:4(5Z,8Z,11Z,14Z)/0:0)	HMDB0011517	42607465		CCCCC
381	HMDB0011489		LysoPE(0:0/20:5(5Z,8Z,11Z,14Z,17Z))	HMDB0011489	53480938		CC/C=
382	HMDB11496		LysoPE(0:0/22:6(4Z,7Z,10Z,13Z,16Z,19Z))	HMDB0011496	53480945		CC/C=
383	HMDB0061698		NA	NA	NA	NA	NA
384	HMDB0061694		NA	NA	NA	NA	NA
385	HMDB0240600		NA	NA	NA	NA	NA
386	HMDB0061695		NA	NA	NA	NA	NA
387	HMDB0240261		LysoPI(18:0/0:0)	HMDB0240261			CCCCC
388	HMDB0061704		NA	NA	NA	NA	NA
389	HMDB0061693		NA	NA	NA	NA	NA
390	HMDB0240597		NA	NA	NA	NA	NA
391	HMDB0013405		PC(o-16:0/18:0)	HMDB0013405	11803170		CCCCC
392	HMDB0011151		PC(O-16:0/18:2(9Z,12Z))	HMDB0011151	6443157		CCCCC
393	HMDB0011342		PE(P-16:0/18:1(9Z))	HMDB0011342	52925128		CCCCC
394	HMDB0011343		PE(P-16:0/18:2(9Z,12Z))	HMDB0011343	52925127		CCCCC
395	HMDB0011206		PC(P-16:0/16:0)	HMDB0011206	11146967		CCCCC
396	HMDB0011207		PC(P-16:0/16:1(9Z))	HMDB0011207	52923882		CCCCC
397	HMDB0011352		PE(P-16:0/20:4(5Z,8Z,11Z,14Z))	HMDB0011352	52925126		CCCCC
398	HMDB0005780		PE(O-16:1(1Z)/22:6(4Z,7Z,10Z,13Z,16Z,19Z))	HMDB0005780	5283497	C00350	CCCCC
399	HMDB0007996		PC(16:0/P-18:1(11Z))	HMDB0007996	53478675	C00157	CCCCC
400	HMDB0011375		PE(P-18:0/18:1(9Z))	HMDB0011375	42607457		CCCCC
401	HMDB0011376		PE(P-18:0/18:2(9Z,12Z))	HMDB0011376	52925079		CCCCC
402	HMDB0011220		PC(P-16:0/20:4(5Z,8Z,11Z,14Z))	HMDB0011220	24779388		CCCCC
403	HMDB0011211		PC(P-16:0/18:2(9Z,12Z))	HMDB0011211	24779386		CCCCC
404	HMDB0005779		PE(O-18:1(1Z)/20:4(5Z,8Z,11Z,14Z))	HMDB0005779	9547058	C00350	CCCCC
405	HMDB0011262		PC(P-18:0/22:6(4Z,7Z,10Z,13Z,16Z,19Z))	HMDB0011262	42607430		CCCCC
406	HMDB0011394		PE(P-18:0/22:6(4Z,7Z,10Z,13Z,16Z,19Z))	HMDB0011394	42607458		CCCCC
407	HMDB0011149		LysoPC(O-18:0)	HMDB0011149	2733532	C04317	CCCCC
408	HMDB0011152		PE(P-16:0e/0:0)	HMDB0011152	42607469		CCCCC
409	HMDB0240598		NA	NA	NA	NA	NA
410	HMDB0000126		Glycerol 3-phosphate	HMDB0000126	439162	C00093	C([C@H
411	HMDB0240316		NA	NA	NA	NA	NA
412	HMDB0011561		MG(14:0/0:0/0:0)	HMDB0011561	10957631		CCCCC
413	HMDB0007102		DG(16:0/18:1(9Z)/0:0)	HMDB0007102	5282283	C13861	CCCCC
414	HMDB0007103		DG(16:0/18:2(9Z,12Z)/0:0)	HMDB0007103	9543695	C00165	CCCCC
415	HMDB0007112		DG(16:0/20:4(5Z,8Z,11Z,14Z)/0:0)	HMDB0007112	9543736	C00165	CCCCC
416	HMDB0007121		DG(16:0/22:6(4Z,7Z,10Z,13Z,16Z,19Z)/0:0)	HMDB0007121	9543827		CCCCC
417	HMDB0007218		DG(18:1(9Z)/18:1(9Z)/0:0)	HMDB0007218	9543716	C00165	CCCCC
418	HMDB0007219		DG(18:1(9Z)/18:2(9Z,12Z)/0:0)	HMDB0007219	9543722	C00165	CCCCC
419	HMDB0007248		DG(18:2(9Z,12Z)/18:2(9Z,12Z)/0:0)	HMDB0007248	9543729	C00165	CCCCC
420	HMDB0007170		DG(18:0/20:4(5Z,8Z,11Z,14Z)/0:0)	HMDB0007170	6438587	C00165	CCCCC
421	HMDB0007228		DG(18:1(9Z)/20:4(5Z,8Z,11Z,14Z)/0:0)	HMDB0007228	9543786	C00165	CCCCC
422	HMDB0007257		DG(18:2(9Z,12Z)/20:4(5Z,8Z,11Z,14Z)/0:0)	HMDB0007257	9543796	C00165	CCCCC
423	HMDB0006790		Galactosylglycerol	HMDB0006790	656504	C05401	C([C@
424	HMDB01480		3-Dehydrosphinganine	HMDB0001480	439853	C02934	CCCCC
425	HMDB0000269		Sphinganine	HMDB0000269	91486	C00836	CCCCC
426	HMDB0001383		Sphinganine 1-phosphate	HMDB0001383	644260	C01120	CCCCC
427	HMDB11760		Cer(d18:0/16:0)	HMDB0011760	5283572		CCCCC
428	HMDB0011761		Cer(d18:0/18:0)	HMDB0011761	5283573		CCCCC
429	HMDB0004949		Ceramide (d18:1/16:0)	HMDB0004949	5283564	C00195	CCCCC
430	HMDB0004950		Ceramide (d18:1/18:0)	HMDB0004950	5283565	C00195	CCCCC
431	HMDB0240686		NA	NA	NA	NA	NA
432	HMDB0011773		NA	NA	NA	NA	NA
433	HMDB0240681		NA	NA	NA	NA	NA
434	HMDB0240678		NA	NA	NA	NA	NA
435	HMDB0240683		NA	NA	NA	NA	NA
436	HMDB0240684		NA	NA	NA	NA	NA
437	HMDB0004951	HMDB0240682	NA	NA	NA	NA	NA
438	HMDB0240679		NA	NA	NA	NA	NA
439	HMDB0240680		NA	NA	NA	NA	NA
440	HMDB0006750		Lactosylceramide (d18:1/16:0)	HMDB0006750	53477895	C01290	CCCCC
441	HMDB0011594		Lactosylceramide (d18:1/22:0)	HMDB0011594	52921641	C01290	CCCCC
442	HMDB0004872		Lactosylceramide (d18:1/24:1(15Z))	HMDB0004872	20057309	C01290	CCCCC
443	HMDB0012085		SM(d18:0/14:0)	HMDB0012085	44260138	C00550	CCCCC
444	HMDB0010168		SM(d18:0/16:0)	HMDB0010168	5283591	C00550	CCCCC

445	HMDB0012091	SM(d18:0/22:0)	HMDB0012091	44260132	C00550	CCCCC
446	HMDB0012087	SM(d18:0/18:0)	HMDB0012087	44260130	C00550	CCCCC
447	HMDB0012090	SM(d18:0/20:0)	HMDB0012090	44260131	C00550	CCCCC
448	HMDB0010169	SM(d18:1/16:0)	HMDB0010169	5283590	C00550	CCCCC
449	HMDB0001348	SM(d18:1/18:0)	HMDB0001348	5283588	C00550	CCCCC
450	HMDB0012103	SM(d18:1/22:0)	HMDB0012103	44260125	C00550	CCCCC
451	HMDB0012105	SM(d18:1/23:0)	HMDB0012105	46891684	C00550	CCCCC
452	HMDB0011697	SM(d18:1/24:0)	HMDB0011697	5283595		CCCCC
453	HMDB0240668	NA	NA	NA	NA	NA
454	HMDB0240644	NA	NA	NA	NA	NA
455	HMDB0012097	SM(d18:1/14:0)	HMDB0012097	11433862		CCCCC
456	HMDB0240637	NA	NA	NA	NA	NA
457	HMDB0240612	NA	NA	NA	NA	NA
458	HMDB0240608	NA	NA	NA	NA	NA
459	HMDB0240617	NA	NA	NA	NA	NA
460	HMDB0240677	NA	NA	NA	NA	NA
461	HMDB0240613	NA	NA	NA	NA	NA
462	HMDB0240638	NA	NA	NA	NA	NA
463	HMDB0240609	NA	NA	NA	NA	NA
464	HMDB0240622	NA	NA	NA	NA	NA
465	HMDB0240620	NA	NA	NA	NA	NA
466	HMDB0012101	SM(d18:1/18:1(9Z))	HMDB0012101	6443882	C00550	CCCCC
467	HMDB0012102	SM(d18:1/20:0)	HMDB0012102	44260124	C00550	CCCCC
468	HMDB0240610	NA	NA	NA	NA	NA
469	HMDB0240632	NA	NA	NA	NA	NA
470	HMDB0240621	NA	NA	NA	NA	NA
471	HMDB0240611	NA	NA	NA	NA	NA
472	HMDB0240619	NA	NA	NA	NA	NA
473	HMDB0240676	NA	NA	NA	NA	NA
474	HMDB0012104	SM(d18:1/22:1(13Z))	HMDB0012104	52931203	C00550	CCCCC
475	HMDB0240672	NA	NA	NA	NA	NA
476	HMDB0240670	NA	NA	NA	NA	NA
477	HMDB0240669	NA	NA	NA	NA	NA
478	HMDB0240634	NA	NA	NA	NA	NA
479	HMDB0011696	NA	NA	NA	NA	NA
480	HMDB0240614	NA	NA	NA	NA	NA
481	HMDB0012107	SM(d18:1/24:1(15Z))	HMDB0012107	44260126	C00550	CCCCC
482	HMDB0240636	NA	NA	NA	NA	NA
483	HMDB0240615	NA	NA	NA	NA	NA
484	HMDB0240671	NA	NA	NA	NA	NA
485	HMDB0240675	NA	NA	NA	NA	NA
486	HMDB0240673	NA	NA	NA	NA	NA
487	HMDB0240674	NA	NA	NA	NA	NA
488	HMDB0000252	Sphingosine	HMDB0000252	5353955	C00319	CCCCC
489	HMDB0000277	Sphingosine 1-phosphate	HMDB0000277	5353956	C06124	CCCCC
490	HMDB0000355	3-Hydroxymethylglutaric acid	HMDB0000355	1662	C03761	CC(CC
491	HMDB0000067	Cholesterol	HMDB0000067	5997	C00187	C[C@H
492	HMDB00653	Cholesterol sulfate	HMDB0000653	65076	C18043	C[C@H
493	HMDB0000921	Cholestenone	HMDB0000921	91477	C00599	C[C@H
494	HMDB0002869	Campesterol	HMDB0002869	5283637	C01789	C[C@H
495	HMDB0006119	7b-Hydroxycholesterol	HMDB0006119	473141		C[C@H
496	HMDB0000619	Cholic acid	HMDB0000619	221493	C00695	C[C@H
497	HMDB0000138	Glycocholic acid	HMDB0000138	23617285	C01921	C[C@H
498	HMDB0000626	Deoxycholic acid	HMDB0000626	222528	C04483	C[C@H
499	HMDB00631	Deoxycholic acid glycine conjugate	HMDB0000631	3035026	C05464	C[C@H
500	HMDB0001517	AICAR	HMDB0001517	65110	C04677	C1=NC
501	HMDB0000175	Inosinic acid	HMDB0000175	8582	C00130	C1=NC
502	HMDB0000195	Inosine	HMDB0000195	6021	C00294	C1=NC
503	HMDB0000157	Hypoxanthine	HMDB0000157	790	C00262	C1=NC
504	HMDB0000292	Xanthine	HMDB0000292	1188	C00385	C1=NC
505	HMDB0000071	Deoxyinosine	HMDB0000071	65058	C05512	C1[C@
506	HMDB0000289	Uric acid	HMDB0000289	1175	C00366	C12=C
507	HMDB0000462	Allantoin	HMDB0000462	204	C01551	C1(C(=
508	HMDB0000538	Adenosine triphosphate	HMDB0000538	5957	C00002	C1=NC
509	HMDB0001341	ADP	HMDB0001341	6022	C00008	C1=NC
510	HMDB0000045	Adenosine monophosphate	HMDB0000045	6083	C00020	C1=NC
511	HMDB0000058	Cyclic AMP	HMDB0000058	6076	C00575	C1[C@
512	HMDB0000536	Adenylsuccinic acid	HMDB0000536	440122	C03794	C1=NC
513	HMDB0000050	Adenosine	HMDB0000050	60961	C00212	C1=NC
514	HMDB0000034	Adenine	HMDB0000034	190	C00147	C1=NC
515	HMDB0000905	Deoxyadenosine monophosphate	HMDB0000905	12599	C00360	C1[C@
516	HMDB0000912	Succinyladenosine	HMDB0000912	20849086		C1=NC
517	HMDB0001397	Guanosine monophosphate	HMDB0001397	6804	C00144	C1=NC
518	HMDB0000133	Guanosine	HMDB0000133	6802	C00387	C1=NC
519	HMDB0000132	Guanine	HMDB0000132	764	C00242	C1=NC
520	HMDB0000897	7-Methylguanine	HMDB0000897	11361	C02242	CN1C=
521	HMDB0000828	Ureidosuccinic acid	HMDB0000828	93072	C00438	C([C@
522	HMDB03349	L-Dihydroorotic acid	HMDB0003349	439216	C00337	C1[C@
523	HMDB0000226	Orotic acid	HMDB0000226	967	C00295	C1=C(
524	HMDB0000788	Orotidine	HMDB0000788	92751	C01103	C1=C(
525	HMDB0000285	Uridine triphosphate	HMDB0000285	6133	C00075	C1=CN
526	HMDB0000295	Uridine 5'-diphosphate	HMDB0000295	6031	C00015	C1=CN
527	HMDB0000288	Uridine 5'-monophosphate	HMDB0000288	6030	C00105	C1=CN
528	HMDB0000296	Uridine	HMDB0000296	6029	C00299	C1=CN
529	HMDB0000300	Uracil	HMDB0000300	1174	C00106	C1=CN
530	HMDB0000767	Pseudouridine	HMDB0000767	15047	C02067	C1=C(
531	HMDB0000884	Ribothymidine	HMDB0000884	445408		CC1=C

532	HMDB0000012	Deoxyuridine	HMDB0000012	13712	C00526	C1[C@
533	HMDB0000026	Ureidopropionic acid	HMDB0000026	111	C02642	C(CNC
534	HMDB0000056	Beta-Alanine	HMDB0000056	239	C00099	C(CN)(
535	HMDB0000095	Cytidine monophosphate	HMDB0000095	8117	C00055	C1=CN
536	HMDB0000089	Cytidine	HMDB0000089	6253	C00475	C1=CN
537	HMDB0000630	Cytosine	HMDB0000630	597	C00380	C1=C(
538	HMDB0001227	5-Thymidylic acid	HMDB0001227	9700	C00364	CC1=C
539	HMDB0000273	Thymidine	HMDB0000273	5789	C00214	CC1=C
540	HMDB0002166	(S)-b-aminoisobutyric acid	HMDB0002166	439434	C03284	C[C@@
541	HMDB61711	Methylphosphate	HMDB0061711	13130		COP(O
542	HMDB0001132	Nicotinic acid mononucleotide	HMDB0001132	53477721	C01185	C1C(C=
543	HMDB0001406	Niacinamide	HMDB0001406	936	C00153	C1=CC
544	HMDB0000229	Nicotinamide ribotide	HMDB0000229	14180	C00455	C1=CC
545	HMDB0000855	Nicotinamide riboside	HMDB0000855	439924	C03150	C1=CC
546	HMDB0001179	Nicotinic acid adenine dinucleotide	HMDB0001179	583440	C00857	C1=CC
547	HMDB0000699	1-Methylnicotinamide	HMDB0000699	457	C02918	C[N+]1
548	HMDB0000875	Trigonelline	HMDB0000875	5570	C01004	C[N+]1
549	HMDB0000210	Pantothenic acid	HMDB0000210	6613	C00864	CC(C)(
550	HMDB0240294	NA	NA	NA	NA	NA
551	HMDB0000943	NA	NA	NA	NA	NA
552	HMDB0062620	NA	NA	NA	NA	NA
553	HMDB0003290	Gulonic acid	HMDB0003290	152304	C00800	C([C@H
554	HMDB0000030	Biotin	HMDB0000030	171548	C00120	C1[C@H
555	HMDB0000121	Folic acid	HMDB0000121	6037	C00504	C1=CC
556	HMDB0003178	Heme	HMDB0003178	26945	C00032	CC1=C
557	HMDB0000235	Thiamine	HMDB0000235	1130	C00378	CC1=C
558	HMDB0002666	Thiamine monophosphate	HMDB0002666	3382778	C01081	CC1=C
559	HMDB0001372	Thiamine pyrophosphate	HMDB0001372	1132	C00068	CC1=C
560	HMDB0000239	Pyridoxine	HMDB0000239	1054	C00314	CC1=N
561	HMDB01319	Pyridoxine 5'-phosphate	HMDB0001319	1055	C00627	CC1=N
562	HMDB0001431	Pyridoxamine	HMDB0001431	1052	C00534	CC1=N
563	HMDB0001555	Pyridoxamine 5'-phosphate	HMDB0001555	1053	C00647	CC1=N
564	HMDB0001491	Pyridoxal 5'-phosphate	HMDB0001491	1051	C00018	CC1=N
565	HMDB0001545	Pyridoxal	HMDB0001545	1050	C00250	CC1=N
566	HMDB0000017	4-Pyridoxic acid	HMDB0000017	6723	C00847	CC1=N
567	HMDB0000714	Hippuric acid	HMDB0000714	464	C01586	C1=CC
568	HMDB0059724	NA	NA	NA	NA	NA
569	HMDB0240459	NA	NA	NA	NA	NA
570	HMDB0011635	p-Cresol sulfate	HMDB0011635	4615423		CC1=C
571	HMDB29737	1H-Indole-3-carboxaldehyde	HMDB0029737	10256	C08493	C1=CC
572	HMDB0029412	Betonicine	HMDB0029412	164642	C08269	C[N+]1
573	HMDB0000625	Gluconic acid	HMDB0000625	10690	C00257	C([C@H
574	HMDB0003045	Ergothioneine	HMDB0003045	5351619	C05570	C[N+](
575	HMDB0003072	Quinic acid	HMDB0003072	6508	C00296	OC1C[
576	HMDB0029422	L-Histidine trimethylbetaine	HMDB0029422	440727	C05575	C[N+](
577	HMDB0033433	(S)-Homostachydrine	HMDB0033433	441447	C08283	C[N+]1
578	HMDB0004827	Proline betaine	HMDB0004827	7016563	C10172	C[N+]1
579	HMDB0001353	2-Keto-3-deoxy-D-gluconic acid	HMDB0001353	194024	C01216	C([C@H
580	HMDB0029968	Ethyl beta-D-glucopyranoside	HMDB0029968	428040		CCOC1
581	HMDB0001859	Acetaminophen	HMDB0001859	1983	C06804	CC(=O
582	HMDB0059911	Paracetamol sulfate	HMDB0059911	83939		CC(=O
583	HMDB0010316	Acetaminophen glucuronide	HMDB0010316	83944		CC(=O
584	HMDB0062547	2-Hydroxyacetaminophen sulfate	HMDB0062547	86290013		CC(=O
585	HMDB0062550	NA	NA	NA	NA	NA
586	HMDB0240217	NA	NA	NA	NA	NA
587	HMDB0001925	Ibuprofen	HMDB0001925	3672	C01588	CC(C)C
588	HMDB0060920	NA	NA	NA	NA	NA
589	HMDB0060564	NA	NA	NA	NA	NA
590	HMDB0014952	Meloxicam	HMDB0014952	54677470	C08169	CC1=C
591	HMDB0014611	Quinine	HMDB0014611	3034034	C06526	COC1=
592	HMDB0042008	NA	NA	NA	NA	NA
593	HMDB0013676	2,6-Dihydroxybenzoic acid	HMDB0013676	9338	C21298	C1=CC
594	HMDB0029415	S-Carboxymethyl-L-cysteine	HMDB0029415	1080		C(C(C(
595	HMDB01448	Sulfate	HMDB0001448	1117	C00059	[O-]S(=
596	HMDB0002520	Beta-Glycerophosphoric acid	HMDB0002520	2526	C02979	C(C(CO
597	HMDB0062164	NA	NA	NA	NA	NA

4 Pathway Analysis

In this step, users are asked to select a pathway library, as well as specify the algorithms for pathway enrichment analysis and pathway topology analysis.

4.1 Pathway Library

There are 15 pathway libraries currently supported, with a total of 1173 pathways :

- Homo sapiens (human) [80]
- Mus musculus (mouse) [82]
- Rattus norvegicus (rat) [81]
- Bos taurus (cow) [81]
- Danio rerio (zebrafish) [81]
- Drosophila melanogaster (fruit fly) [79]
- Caenorhabditis elegans (nematode) [78]
- Saccharomyces cerevisiae (yeast) [65]
- Oryza sativa japonica (Japanese rice) [83]
- Arabidopsis thaliana (thale cress) [87]
- Escherichia coli K-12 MG1655 [87]
- Bacillus subtilis [80]
- Pseudomonas putida KT2440 [89]
- Staphylococcus aureus N315 (MRSA/VSSA)[73]
- Thermotoga maritima [57]

Your selected pathway library code is **hsa** (KEGG organisms abbreviation).

4.2 Over Representation Analysis

Over-representation analysis tests if a particular group of compounds is represented more than expected by chance within the user uploaded compound list. In the context of pathway analysis, we are testing if compounds involved in a particular pathway are enriched compared to random hits. MetPA offers two of the most commonly used methods for over-representation analysis:

- Fishers'Exact test
- Hypergeometric Test

Please note, MetPA uses one-tailed Fisher's exact test which will give essentially the same result as the result calculated by the hypergeometric test.

The selected over-representation analysis method is **Hypergeometric test**.

4.3 Pathway Topology Analysis

The structure of biological pathways represent our knowledge about the complex relationships among molecules within a cell or a living organism. However, most pathway analysis algorithms fail to take structural information into consideration when estimating which pathways are significantly changed under conditions of study. It is well-known that changes in more important positions of a network will trigger a more severe impact on the pathway than changes occurred in marginal or relatively isolated positions.

The pathway topology analysis uses two well-established node centrality measures to estimate node importance - **degree centrality** and **betweenness centrality**. Degree centrality is defined as the number of links occurred upon a node. For a directed graph there are two types of degree: in-degree for links come from other nodes, and out-degree for links initiated from the current node. Metabolic networks are directed graph. Here we only consider the out-degree for node importance measure. It is assumed that nodes upstream will have regulatory roles for the downstream nodes, not vice versa. The betweenness centrality measures the number of shortest paths going through the node. Since the metabolic network is directed, we use the relative betweenness centrality for a metabolite as the importance measure. The degree centrality measure focuses more on local connectivities, while the betweenness centrality measure focuses more on global network topology. For more detailed discussions on various graph-based methods for analyzing biological networks, please refer to the article by Tero Aittokallio, T. et al. [1]

Please note, for comparison among different pathways, the node importance values calculated from centrality measures are further normalized by the sum of the importance of the pathway. Therefore, the total/maximum importance of each pathway is 1; the importance measure of each metabolite node is actually the percentage w.r.t the total pathway importance, and the pathway impact value is the cumulative percentage from the matched metabolite nodes.

Your selected node importance measure for topological analysis is **relative betweenness centrality**.

5 Pathway Analysis Result

The results from pathway analysis are presented graphically as well as in a detailed table.

A Google-map style interactive visualization system was implemented to facilitate data exploration. The graphical output contains three levels of view: **metabolome view**, **pathway view**, and **compound view**. Only the metabolome view is shown below. Pathway views and compound views are generated dynamically based on your interactions with the visualization system. They are available in your downloaded files.

[1] Tero Aittokallio and Benno Schwikowski. *Graph-based methods for analyzing networks in cell biology*, Briefings in Bioinformatics 2006 7(3):243-255

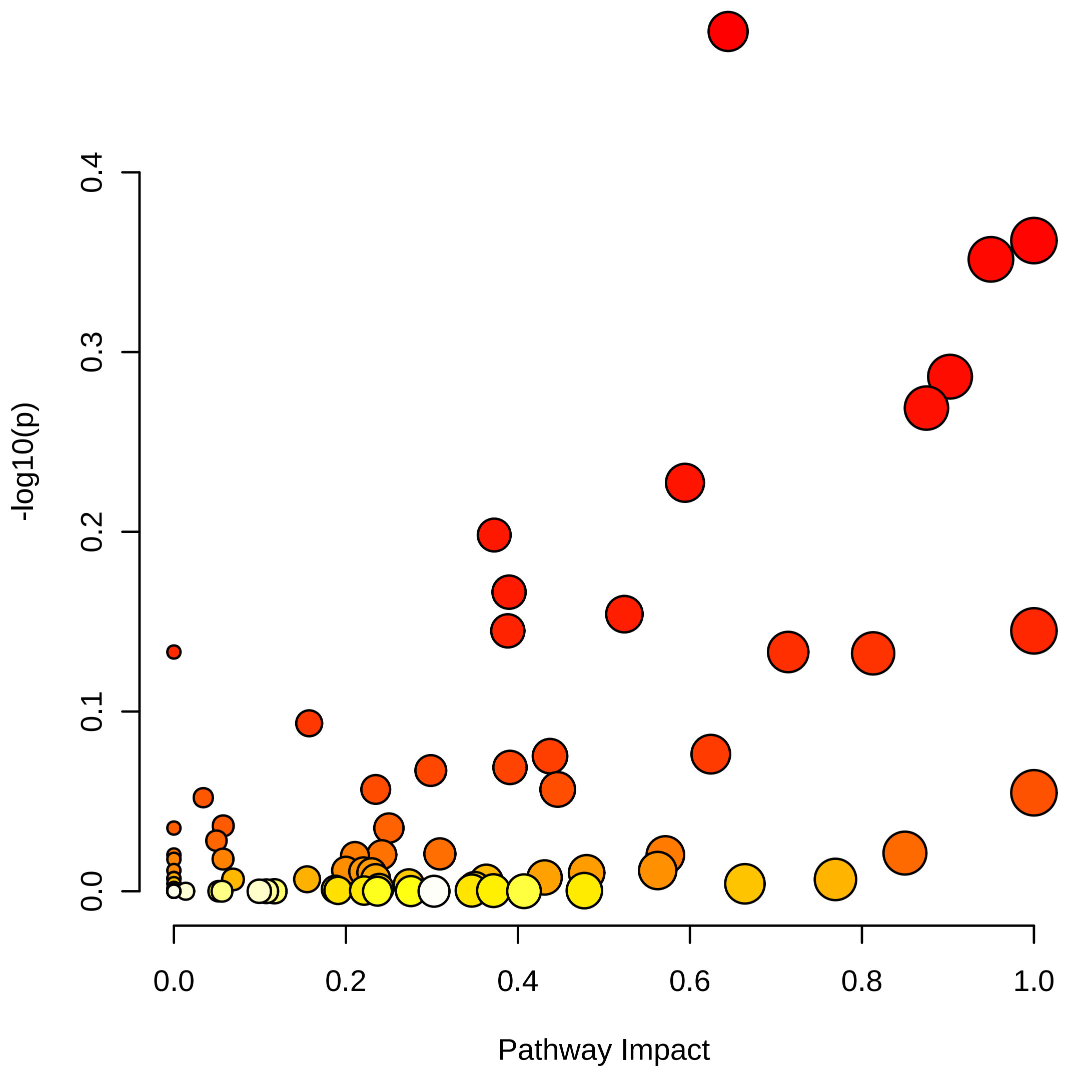

Figure 1: Summary of Pathway Analysis

The table below shows the detailed results from the pathway analysis. Since we are testing many pathways at the same time, the statistical p values from enrichment analysis are further adjusted for multiple testings. In particular, the **Total** is the total number of compounds in the pathway; the **Hits** is the actually matched number from the user uploaded data; the **Raw p** is the original p value calculated from the enrichment analysis; the **Holm p** is the p value adjusted by Holm-Bonferroni method; the **FDR p** is the p value adjusted using False Discovery Rate; the **Impact** is the pathway impact value calculated from pathway topology analysis.

Table 2: Result from Pathway Analysis

	Total	Expected	Hits	Raw p	-log10(p)	Holm adjust	FDR	Impact
Alpha Linolenic Acid and Linoleic Acid Metabolism	16	7.64	9	3.32E-01	4.78E-01	1.00E+00	1.00E+00	0.64
Phosphatidylethanolamine Biosynthesis	13	6.21	7	4.34E-01	3.62E-01	1.00E+00	1.00E+00	1.00
Thiamine Metabolism	9	4.30	5	4.45E-01	3.52E-01	1.00E+00	1.00E+00	0.95
Phosphatidylcholine Biosynthesis	18	8.60	9	5.17E-01	2.86E-01	1.00E+00	1.00E+00	0.90
Lactose Synthesis	14	6.69	7	5.38E-01	2.69E-01	1.00E+00	1.00E+00	0.88
Sphingolipid Metabolism	36	17.20	17	5.93E-01	2.27E-01	1.00E+00	1.00E+00	0.59
Vitamin B6 Metabolism	15	7.17	7	6.34E-01	1.98E-01	1.00E+00	1.00E+00	0.37
Glycolysis	20	9.55	9	6.82E-01	1.66E-01	1.00E+00	1.00E+00	0.39
Taurine and Hypotaurine Metabolism	9	4.30	4	7.01E-01	1.54E-01	1.00E+00	1.00E+00	0.52
Carnitine Synthesis	16	7.64	7	7.16E-01	1.45E-01	1.00E+00	1.00E+00	0.39
Nucleotide Sugars Metabolism	16	7.64	7	7.16E-01	1.45E-01	1.00E+00	1.00E+00	1.00
Homocysteine Degradation	7	3.34	3	7.36E-01	1.33E-01	1.00E+00	1.00E+00	0.00
Malate-Aspartate Shuttle	7	3.34	3	7.36E-01	1.33E-01	1.00E+00	1.00E+00	0.71
Phosphatidylinositol Phosphate Metabolism	14	6.69	6	7.37E-01	1.32E-01	1.00E+00	1.00E+00	0.81
Mitochondrial Electron Transport Chain	15	7.17	6	8.06E-01	9.34E-02	1.00E+00	1.00E+00	0.16
Phospholipid Biosynthesis	25	11.94	10	8.39E-01	7.63E-02	1.00E+00	1.00E+00	0.62
Betaine Metabolism	18	8.60	7	8.41E-01	7.52E-02	1.00E+00	1.00E+00	0.44
Urea Cycle	23	10.99	9	8.53E-01	6.89E-02	1.00E+00	1.00E+00	0.39
Cardiolipin Biosynthesis	11	5.25	4	8.57E-01	6.72E-02	1.00E+00	1.00E+00	0.30
Starch and Sucrose Metabolism	26	12.42	10	8.78E-01	5.66E-02	1.00E+00	1.00E+00	0.23
Beta-Alanine Metabolism	26	12.42	10	8.78E-01	5.66E-02	1.00E+00	1.00E+00	0.45
Alanine Metabolism	14	6.69	5	8.81E-01	5.48E-02	1.00E+00	1.00E+00	1.00
Galactose Metabolism	31	14.81	12	8.87E-01	5.21E-02	1.00E+00	1.00E+00	0.03
Ammonia Recycling	25	11.94	9	9.20E-01	3.64E-02	1.00E+00	1.00E+00	0.06
Pyruvaldehyde Degradation	7	3.34	2	9.22E-01	3.52E-02	1.00E+00	1.00E+00	0.00
Biotin Metabolism	7	3.34	2	9.22E-01	3.52E-02	1.00E+00	1.00E+00	0.25
Beta Oxidation of Very Long Chain Fatty Acids	13	6.21	4	9.37E-01	2.81E-02	1.00E+00	1.00E+00	0.05
Aspartate Metabolism	34	16.24	12	9.52E-01	2.12E-02	1.00E+00	1.00E+00	0.85
Glutathione Metabolism	19	9.08	6	9.53E-01	2.08E-02	1.00E+00	1.00E+00	0.31
Methionine Metabolism	39	18.63	14	9.54E-01	2.03E-02	1.00E+00	1.00E+00	0.24
Phenylacetate Metabolism	8	3.82	2	9.55E-01	2.02E-02	1.00E+00	1.00E+00	0.00
Glycerol Phosphate Shuttle	8	3.82	2	9.55E-01	2.02E-02	1.00E+00	1.00E+00	0.57
Trehalose Degradation	11	5.25	3	9.56E-01	1.96E-02	1.00E+00	1.00E+00	0.21
Spermidine and Spermine Biosynthesis	14	6.69	4	9.60E-01	1.79E-02	1.00E+00	1.00E+00	0.06
Oxidation of Branched Chain Fatty Acids	22	10.51	7	9.60E-01	1.78E-02	1.00E+00	1.00E+00	0.00
Lactose Degradation	9	4.30	2	9.74E-01	1.15E-02	1.00E+00	1.00E+00	0.00
De Novo Triacylglycerol Biosynthesis	9	4.30	2	9.74E-01	1.15E-02	1.00E+00	1.00E+00	0.20
Glucose-Alanine Cycle	9	4.30	2	9.74E-01	1.15E-02	1.00E+00	1.00E+00	0.56
Amino Sugar Metabolism	31	14.81	10	9.75E-01	1.10E-02	1.00E+00	1.00E+00	0.22
Citric Acid Cycle	26	12.42	8	9.76E-01	1.04E-02	1.00E+00	1.00E+00	0.23
Transfer of Acetyl Groups into Mitochondria	18	8.60	5	9.77E-01	1.03E-02	1.00E+00	1.00E+00	0.48
Nicotinate and Nicotinamide Metabolism	32	15.29	10	9.82E-01	7.68E-03	1.00E+00	1.00E+00	0.43
Threonine and 2-Oxobutanoate Degradation	13	6.21	3	9.84E-01	7.13E-03	1.00E+00	1.00E+00	0.00
Pentose Phosphate Pathway	27	12.90	8	9.84E-01	7.07E-03	1.00E+00	1.00E+00	0.23
Plasmalogen Synthesis	16	7.64	4	9.84E-01	7.02E-03	1.00E+00	1.00E+00	0.00
Glycine and Serine Metabolism	50	23.89	17	9.85E-01	6.66E-03	1.00E+00	1.00E+00	0.15
Histidine Metabolism	35	16.72	11	9.85E-01	6.58E-03	1.00E+00	1.00E+00	0.77
Pantothenate and CoA Biosynthesis	19	9.08	5	9.85E-01	6.58E-03	1.00E+00	1.00E+00	0.07
Arginine and Proline Metabolism	48	22.93	16	9.87E-01	5.75E-03	1.00E+00	1.00E+00	0.36
Riboflavin Metabolism	14	6.69	3	9.90E-01	4.23E-03	1.00E+00	1.00E+00	0.00
Pyrimidine Metabolism	54	25.80	18	9.91E-01	4.14E-03	1.00E+00	1.00E+00	0.66
Glycerolipid Metabolism	23	10.99	6	9.91E-01	3.88E-03	1.00E+00	1.00E+00	0.27
Warburg Effect	49	23.41	15	9.96E-01	1.76E-03	1.00E+00	1.00E+00	0.35
Phytanic Acid Peroxisomal Oxidation	19	9.08	4	9.96E-01	1.60E-03	1.00E+00	1.00E+00	0.00
Sulfate/Sulfite Metabolism	19	9.08	4	9.96E-01	1.60E-03	1.00E+00	1.00E+00	0.24
Glutamate Metabolism	45	21.50	13	9.97E-01	1.13E-03	1.00E+00	1.00E+00	0.19
Catecholamine Biosynthesis	14	6.69	2	9.99E-01	6.35E-04	1.00E+00	1.00E+00	0.00
Purine Metabolism	63	30.09	19	9.99E-01	4.72E-04	1.00E+00	1.00E+00	0.19
Fructose and Mannose Degradation	28	13.38	6	9.99E-01	3.83E-04	1.00E+00	1.00E+00	0.35
Phenylalanine and Tyrosine Metabolism	25	11.94	5	9.99E-01	3.71E-04	1.00E+00	1.00E+00	0.22
Ethanol Degradation	15	7.17	2	9.99E-01	3.50E-04	1.00E+00	1.00E+00	0.48
Inositol Phosphate Metabolism	22	10.51	4	9.99E-01	3.38E-04	1.00E+00	1.00E+00	0.37
Butyrate Metabolism	16	7.64	2	1.00E+00	1.92E-04	1.00E+00	1.00E+00	0.00
Ketone Body Metabolism	12	5.73	1	1.00E+00	1.69E-04	1.00E+00	1.00E+00	0.00
Estrone Metabolism	20	9.55	3	1.00E+00	1.58E-04	1.00E+00	1.00E+00	0.00
Mitochondrial Beta-Oxidation of Long Chain Saturated Fatty Acids	24	11.46	4	1.00E+00	1.16E-04	1.00E+00	1.00E+00	0.00

Mitochondrial Beta-Oxidation of Short Chain Saturated Fatty Acids	17	8.12	2	1.00E+00	1.05E-04	1.00E+00	1.00E+00	0.00
Pyruvate Metabolism	37	17.68	8	1.00E+00	9.09E-05	1.00E+00	1.00E+00	0.28
Lysine Degradation	20	9.55	2	1.00E+00	1.66E-05	1.00E+00	1.00E+00	0.24
Folate Metabolism	24	11.46	3	1.00E+00	1.57E-05	1.00E+00	1.00E+00	0.00
Selenoamino Acid Metabolism	28	13.38	4	1.00E+00	1.27E-05	1.00E+00	1.00E+00	0.00
Inositol Metabolism	28	13.38	4	1.00E+00	1.27E-05	1.00E+00	1.00E+00	0.41
Valine, Leucine and Isoleucine Degradation	51	24.36	11	1.00E+00	8.14E-06	1.00E+00	1.00E+00	0.05
Ubiquinone Biosynthesis	18	8.60	1	1.00E+00	3.16E-06	1.00E+00	1.00E+00	0.00
Androstenedione Metabolism	23	10.99	2	1.00E+00	2.56E-06	1.00E+00	1.00E+00	0.00
Porphyrin Metabolism	36	17.20	5	1.00E+00	1.01E-06	1.00E+00	1.00E+00	0.12
Androgen and Estrogen Metabolism	29	13.85	3	1.00E+00	7.98E-07	1.00E+00	1.00E+00	0.00
Fatty Acid Biosynthesis	33	15.76	4	1.00E+00	7.17E-07	1.00E+00	1.00E+00	0.06
Mitochondrial Beta-Oxidation of Medium Chain Saturated Fatty Acids	22	10.51	1	1.00E+00	2.18E-07	1.00E+00	1.00E+00	0.00
Bile Acid Biosynthesis	59	28.18	10	1.00E+00	3.19E-08	1.00E+00	1.00E+00	0.11
Fatty Acid Metabolism	40	19.11	4	1.00E+00	1.11E-08	1.00E+00	1.00E+00	0.00
Retinol Metabolism	30	14.33	1	1.00E+00	9.98E-10	1.00E+00	1.00E+00	0.00
Fatty Acid Elongation In Mitochondria	33	15.76	1	1.00E+00	1.30E-10	1.00E+00	1.00E+00	0.00
Tryptophan Metabolism	55	26.27	6	1.00E+00	1.30E-10	1.00E+00	1.00E+00	0.10
Steroid Biosynthesis	43	20.54	3	1.00E+00	1.28E-10	1.00E+00	1.00E+00	0.01
Tyrosine Metabolism	55	26.27	5	1.00E+00	1.24E-11	1.00E+00	1.00E+00	0.00
Steroidogenesis	42	20.06	1	1.00E+00	2.74E-13	1.00E+00	1.00E+00	0.00
Arachidonic Acid Metabolism	65	31.05	3	1.00E+00	9.64E-17	1.00E+00	1.00E+00	0.30

6 Appendix: R Command History

```
 [1] "mSet<-InitDataObjects(\"conc\", \"pathora\", FALSE)"
 [2] "cmpd.vec<-c(\"HMDB0000123\",\"HMDB0000532\",\"HMDB0000271\",\"HMDB0000092\",\"HMDB0000043\",\"H
 [3] "mSet<-Setup.MapData(mSet, cmpd.vec);"
 [4] "mSet<-CrossReferencing(mSet, \"hmdb\");"
 [5] "mSet<-CreateMappingResultTable(mSet)"
 [6] "mSet<-SetKEGG.PathLib(mSet, \"pfa\", \"current\")"
 [7] "mSet<-SetMetabolomeFilter(mSet, F);"
 [8] "mSet<-CalculateOraScore(mSet, \"rbc\", \"hyperg\")"
 [9] "mSet<-PlotPathSummary(mSet, F, \"path_view_2_\", \"png\", 72, width=NA, NA, NA )"
[10] "mSet<-PlotKEGGPath(mSet, \"Alanine, aspartate and glutamate metabolism\",576, 480, \"png\", NUL
[11] "mSet<-RerenderMetPAGraph(mSet, \"zoom1657122547702.png\",576.0, 480.0, 100.0)"
[12] "mSet<-PlotKEGGPath(mSet, \"Alanine, aspartate and glutamate metabolism\",576, 480, \"png\", NUL
[13] "mSet<-PlotKEGGPath(mSet, \"Vitamin B6 metabolism\",576, 480, \"png\", NULL)"
[14] "mSet<-CreateMappingResultTable(mSet)"
[15] "mSet<-PerformDetailMatch(mSet, \"HMDB0001344\");"
[16] "mSet<-GetCandidateList(mSet);"
[17] "mSet<-CreateMappingResultTable(mSet)"
[18] "mSet<-SetSMPDB.PathLib(mSet, \"hsa\")"
[19] "mSet<-SetOrganism(mSet, \"hsa\")"
[20] "mSet<-SetMetabolomeFilter(mSet, F);"
[21] "mSet<-CalculateOraScore(mSet, \"rbc\", \"hyperg\")"
[22] "mSet<-PlotPathSummary(mSet, F, \"path_view_3_\", \"png\", 72, width=NA, NA, NA )"
[23] "mSet<-SetSMPDB.PathLib(mSet, \"hsa\")"
[24] "mSet<-SetOrganism(mSet, \"hsa\")"
[25] "mSet<-SetMetabolomeFilter(mSet, F);"
[26] "mSet<-CalculateOraScore(mSet, \"rbc\", \"hyperg\")"
[27] "mSet<-PlotPathSummary(mSet, F, \"path_view_4_\", \"png\", 72, width=NA, NA, NA )"
[28] "mSet<-SetSMPDB.PathLib(mSet, \"hsa\")"
[29] "mSet<-SetOrganism(mSet, \"hsa\")"
[30] "mSet<-SetMetabolomeFilter(mSet, F);"
[31] "mSet<-CalculateOraScore(mSet, \"rbc\", \"hyperg\")"
[32] "mSet<-PlotPathSummary(mSet, F, \"path_view_5_\", \"png\", 72, width=NA, NA, NA )"
[33] "mSet<-SaveTransformedData(mSet)"
[34] "mSet<-PreparePDFReport(mSet, \"guest5859144621155800605\")\n"
```

The report was generated on Wed Jul 6 11:53:09 2022 with R version 4.1.3 (2022-03-10), OS system: Linux, version: 30 20.04.1-Ubuntu SMP Tue Apr 26 03:01:25 UTC 2022 .

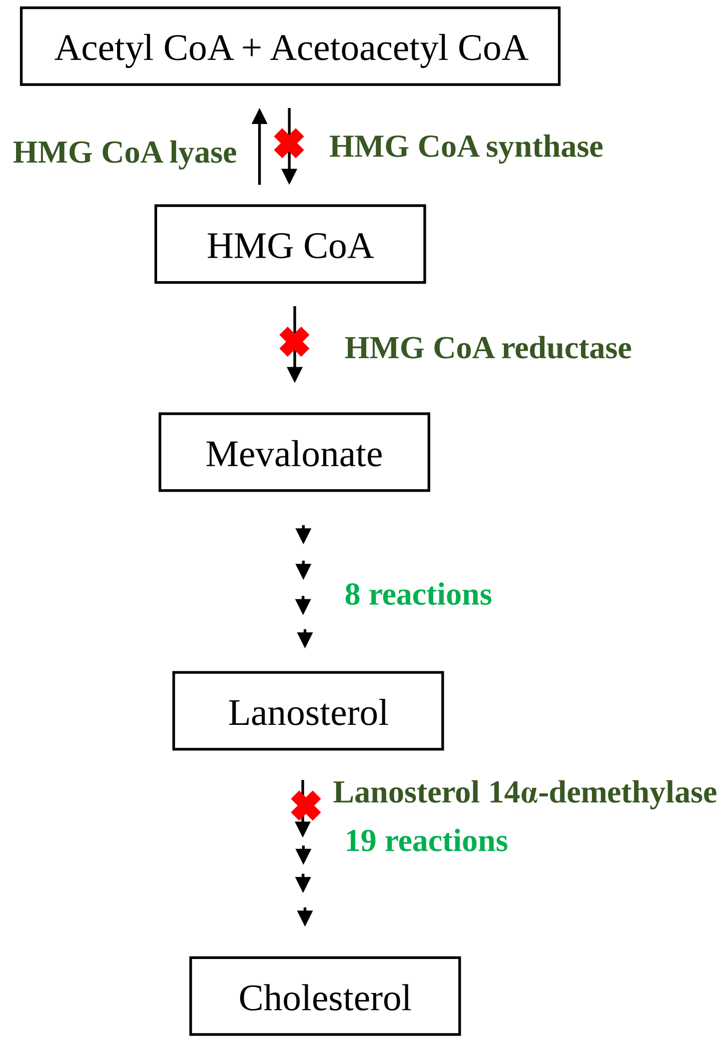

Figure S1: *In silico* analysis of key enzymes in cholesterol biosynthesis shows absence of these enzymes in *B. divergens*. The main enzymes are in dark green. The number of reactions linking the metabolites are mentioned in lime green. A red cross signifies that these enzymes were not found in our BLASTp analysis in *B. divergens*.

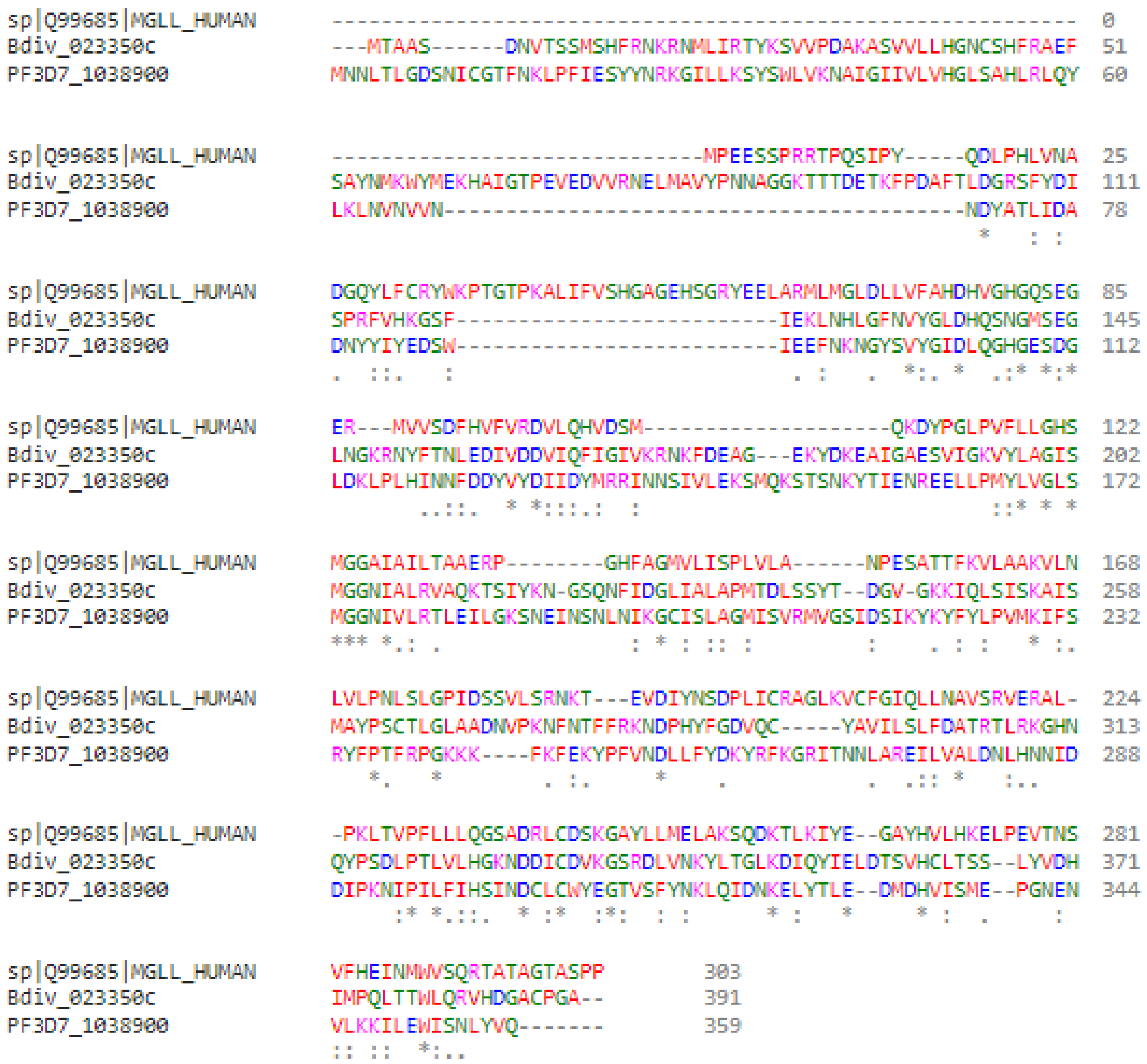

Figure S2: Multiple sequence analysis of MAGL from human, *P. falciparum* and *B. divergens*. Sequence analysis of Human monoacylglycerol lipase (MGLL_HUMAN-UniProt), lipase from *P. falciparum* (PF3D7_1038900) which is a target for orlistat, and its homolog in *B. divergens* (Bdiv_023350c). Two "G-X-S-X-G" motifs are highlighted in black rectangles. Residues of the catalytic triad (serine "S", histidine "H", and aspartate "D") are marked in blue rectangles while the cysteine "C" residue adjacent to the active site and required for ligand recognition is marked in a red rectangle.
